# The N-Ank protein Ankrd24 shapes neurons and couples to syndapin and the F-actin promoter Cobl-like

**DOI:** 10.1083/jcb.202509074

**Published:** 2026-10-21

**Authors:** Astha Jain, Maryam Izadi-Seitz, Yuanyuan Ji, Eric Seemann, Britta Qualmann, Michael M. Kessels

**Affiliations:** 1 Institute of Biochemistry I, Jena University Hospital - Friedrich Schiller University Jena, Jena, Germany

## Abstract

Proper neuromorphogenesis is pivotal for neuronal network formation and brain function. The underlying cell biological tasks are complex, as they include both remodeling of membrane topologies and of cytoskeletal structures. Yet, mechanisms coordinating these two different cell biological functions are largely unknown. Here, we molecularly and functionally characterize ankyrin repeat domain-containing protein 24 (Ankrd24) and demonstrate that this ankyrin-repeat protein is able to interact with and bend membranes and mechanistically and functionally belongs to the recently proposed N-Ank superfamily. Mutational analyses revealed that the N-Ank functions of Ankrd24 are indispensable for Ankrd24’s role in neuromorphogenesis. Ankrd24 furthermore physically cooperated with the F-BAR domain protein syndapin I (PACSIN1) and with Cobl-like (Cobll1), a protein promoting actin filament formation, by ternary complex formation. Both partners were critical for Ankrd24-mediated dendritic arborization. This identified Ankrd24 as critical part of a larger, coordinated effort in inducing new dendritic branches. Our work thereby sheds light on the molecular mechanisms underlying the complex task of proper neuronal arbor development.

## Teaser

Proper neuromorphogenesis is pivotal for neuronal network formation and brain function. Jain, Izadi-Seitz and Ji et al. identify mechanisms and cellular functions of the N-Ank protein Ankrd24 in shaping nerve cells by coordinating remodeling of membrane topologies and of cytoskeletal structures and thereby shed light on proper neuronal arbor development.

## Introduction

Changes of cell shape are key in the differentiation, function, and plastic rearrangement of the specialized cells of multicellular organisms. Membrane-sculpting proteins thus are in the spotlight of current research. Predominantly based on molecular/mechanistic examinations of ankycorbin (RAI14), a new superfamily of membrane shaping proteins, termed N-Ank proteins, has been proposed (Wolf et al., 2019). According to this hypothesis, N-Ank proteins use short ankyrin repeat arrays in combination with an N-terminal amphipathic helix for sensing and inducing membrane curvature by membrane association and partial insertion (Wolf et al., 2019).

The founding member of the N-Ank family, ankycorbin, critically relies on its N-Ank module, self-association and the syndapin (PACSIN) subfamily of F-BAR proteins (Itoh et al., 2005; Qualmann et al., 2011; Carman and Dominguez, 2018; Simunovic et al., 2019) for its role in early neuronal development (Wolf et al., 2019; Izadi et al., 2023). The importance of an intact N-Ank module was furthermore demonstrated for another superfamily member, ANKRD26. Mutations leading to an N-terminally truncated ANKRD26 were associated with acute myeloid leukemia (Marconi et al., 2017). Molecular and functional characterizations of such a mutant revealed disruptions of N-Ank functionalities and their critical role in cell differentiation (Englisch et al., 2024). Functional analyses of all other suggested N-Ank proteins with regard to putative membrane binding and membrane shaping are still lacking.

Putative N-Ank modules can be identified in at least 18 genes in humans. Ankyrin repeat domain-containing protein 24 (Ankrd24) was suggested to be a member of the first subfamily of N-Ank proteins comprising ankycorbin, uveal autoantigen with coiled-coil domains and ankyrin repeats (UACA), Ankrd35 and Ankrd24 (Wolf et al., 2019). Knowledge of Ankrd24 properties and functions is still sparse. A splice variant of Ankrd24 was discovered in the cochlea. It localized to and organized the rootlets of stereocilia of inner hair cells by associating with particular TRIOBP splice variants, TRIOBP-1 and TRIOBP-5 (Krey et al., 2022). TRIOBP-1 is an F-actin–binding protein and also called Tara (Seipel et al., 2001). TRIOBP-4 and -5 deficiencies led to hearing loss (Kitajiri et al., 2010; Katsuno et al., 2019). In line, deficiency of Ankrd24 was also associated with premature hearing loss (Krey et al., 2022; Kazemi et al., 2025). In SH-SY-5Y cells, ANKRD24 was upregulated upon 3-hydroxy-3-methylglutaryl-CoA synthase 2 (HMGCS2) overexpression. In U251MG glioblastoma cells, ANKRD24 played some role in autophagy processes, such as that induced by HMGCS2 overexpression, and siRNA-mediated knockdown of ANKRD24 correspondingly led to the accumulation of phosphoTau in this cell line (Hu et al., 2023). PhosphoTau accumulation in neurons is a hallmark of Alzheimer’s disease (Grundke-Iqbal et al., 1986; Iqbal et al., 2008). This may thus imply some role of ANKRD24 in Alzheimer’s disease. Yet, Ankrd24 functions in neurons remained unknown.

We here show that Ankrd24 promotes the induction of dendritic branches and thereby plays a critical role in dendritic arbor formation and extension. Molecular mechanistic and functional analyses furthermore revealed that Ankrd24 indeed is an N-Ank protein, as Ankrd24 used a combination of amphipathic helix and ankyrin repeat array for membrane binding and curvature induction, and these molecular mechanisms were critical in dendritic arbor development.

Interestingly, Ankrd24’s functions in addition required a physical and functional coupling to syndapin I (Qualmann et al., 1999; Koch et al., 2011; Koch et al., 2020). This suggests cooperative functions of different classes of membrane shapers in neuronal development. Ankrd24-mediated dendritic arborization furthermore turned out to critically depend on the actin filament formation–promoting protein Cobl-like (Izadi et al., 2018). Cobl-like shares some similarities with the actin nucleator Cobl (Ahuja et al., 2007) and cooperates with Cobl in dendritic arbor formation (Izadi et al., 2021). Furthermore, Cobl-like is implicated in cancer and obesity (Mancina et al., 2013; Takayama et al., 2018; Glunk et al., 2023). Ankrd24 formed ternary complexes with Cobl-like using syndapin I as a bridge.

Together, our molecular and functional analyses provide insights into the mechanisms and cellular functions of the N-Ank protein Ankrd24 in shaping nerve cells and thereby shed light on the complex task of proper neuronal network formation.

## Results

### Ankrd24 is a syndapin-binding partner

**Figure 1.**
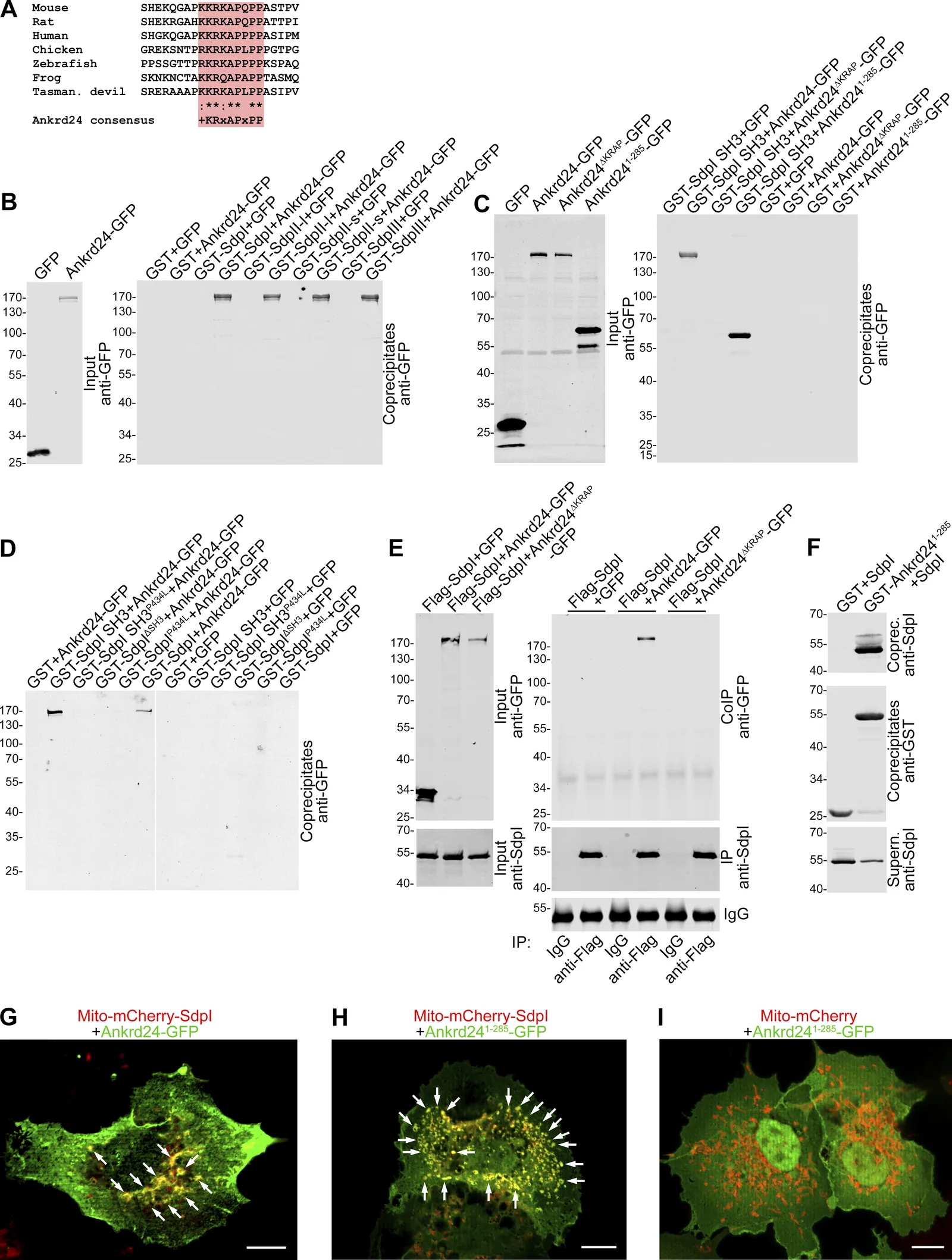
**Ankrd24 binds to syndapin proteins via a distinct, evolutionary highly conserved motif. (A)** Alignment showing the predicted syndapin-binding site of Ankrd24 (red box) and its high conservation throughout evolution. Mouse (*M. musculus*) Ankrd24 (UniProt Q9UPS8; NCBI: XP_006514159.1), KRAP motif, aa 274–282; rat (*R. norvegicus*) Ankrd24 (NCBI: XM_039078672.1), KRAP motif, aa 274–282; human (*Homo sapiens*) ANKRD24 (UniProt: Q8TF21-1), KRAP motif, aa 303–311; chicken (*Gallus gallus*) Ankrd24 (UniProt: A0A8V1AAK0), KRAP motif, aa 286–294; zebrafish (*Danio rerio*) Ankrd24, (UniProt: A0A8N7UVY2), KRAP motif, aa 276–284; frog (*Xenopus tropicalis*) Ankrd24 (UniProt: A0A6I8SDW8), KRAP motif, aa 269–277; tasmanian devil (*Sarcophilus harrisii*) Ankrd24 (UniProt: G3W900), KRAP motif, aa 269–277. **(B)** Anti-GFP immunoblotting analyses of coprecipitation experiments with lysates of HEK293 cells overexpressing Ankrd24-GFP or GFP and immobilized GST-tagged full-length syndapin I (GST-SdpI), syndapin II-long (GST-SdpII-l), syndapin II-short (GST-SdpII-s), syndapin III (GST-SdpIII), or GST, respectively. Ankrd24-GFP specifically coprecipitated with GST-SdpI, GST-SdpII-l, GST-SdpII-s, and GST-SdpIII. For supernatant analyses, see Fig. S1 A. **(C)** Anti-GFP immunoblotting analyses of coprecipitation experiments with lysates of L929 cells overexpressing GFP, Ankrd24-GFP, Ankrd24^ΔKRAP^-GFP, or Ankrd24^1-285^-GFP and immobilized GST-tagged syndapin I SH3 domain (GST-SdpI SH3) and GST, respectively. For supernatant analyses, see Fig. S1 B. **(D)** Anti-GFP immunoblottings of coprecipitation experiments with lysates of L929 cells overexpressing either GFP or Ankrd24-GFP and immobilized GST as well as GST-tagged syndapin I SH3 domain (GST-SdpI SH3), the P434L mutant thereof (GST-SdpI SH3^P434L^), syndapin I^ΔSH3 ^(GST-SdpI^^ΔSH3^^), full-length syndapin I carrying the P434L point mutation (GST-SdpI^P434L^), and wild-type syndapin I (GST-SdpI), respectively. Note that syndapin I associates with Ankrd24 via the syndapin I SH3 domain in a specific manner and that P434L mutation in both the SH3 domain and in the full-length context of syndapin I disrupts the association with Ankrd24-GFP. White line indicates omitted lanes of the blot. For supernatant analyses, see Fig. S1 C. **(E)** Coimmunoprecipitation analyses of Flag-syndapin I (Flag-SdpI) and Ankrd24-GFP complexes. Note that syndapin I specifically coimmunoprecipitated Ankrd24-GFP, but not the mutant Ankrd24^ΔKRAP^-GFP (Ankrd24^Δ273-286^–GFP) lacking the proposed syndapin I interaction motif. For supernatant analyses, see Fig. S1 D. **(F)** Immunoblotting analyses of reconstitutions of complex formation between immobilized GST-Ankrd24^1-285^ but not GST with purified recombinant syndapin I proving a direct interaction. **(G–I)** Maximum intensity projections (MIPs) of subcellular recruitments of Ankrd24-GFP (G) and Ankrd24^1-285^-GFP (H) to mitochondrial membranes of COS-7 cells decorated with syndapin I (mito-mCherry-SdpI) as well as MIPs of corresponding control experiments with mito-mCherry (I). Arrows mark examples of clear Ankrd24 and Ankrd24^1-285^-GFP recruitment to the syndapin-enriched sites. Bars, 10 µm. For individual fluorescence channels and for intensity line scans, see Fig. S1, E–G. Source data are available for this figure: SourceData F1.

Our in silico analyses suggested that two N-Ank protein superfamily members (Wolf et al., 2019) are distinguished from all other members of this superfamily known to date: ankycorbin, which was recently demonstrated to physically and functionally interact with F-BAR proteins of the syndapin subfamily (also called PACSINs) via a short, strictly conserved sequence composed of the aa KRKAPPPP (Izadi et al., 2023), and Ankrd24, which shows a related motif following the consensus +KRxAPxPP (KRAP motif) (Fig. 1 A).

**Figure S1.**
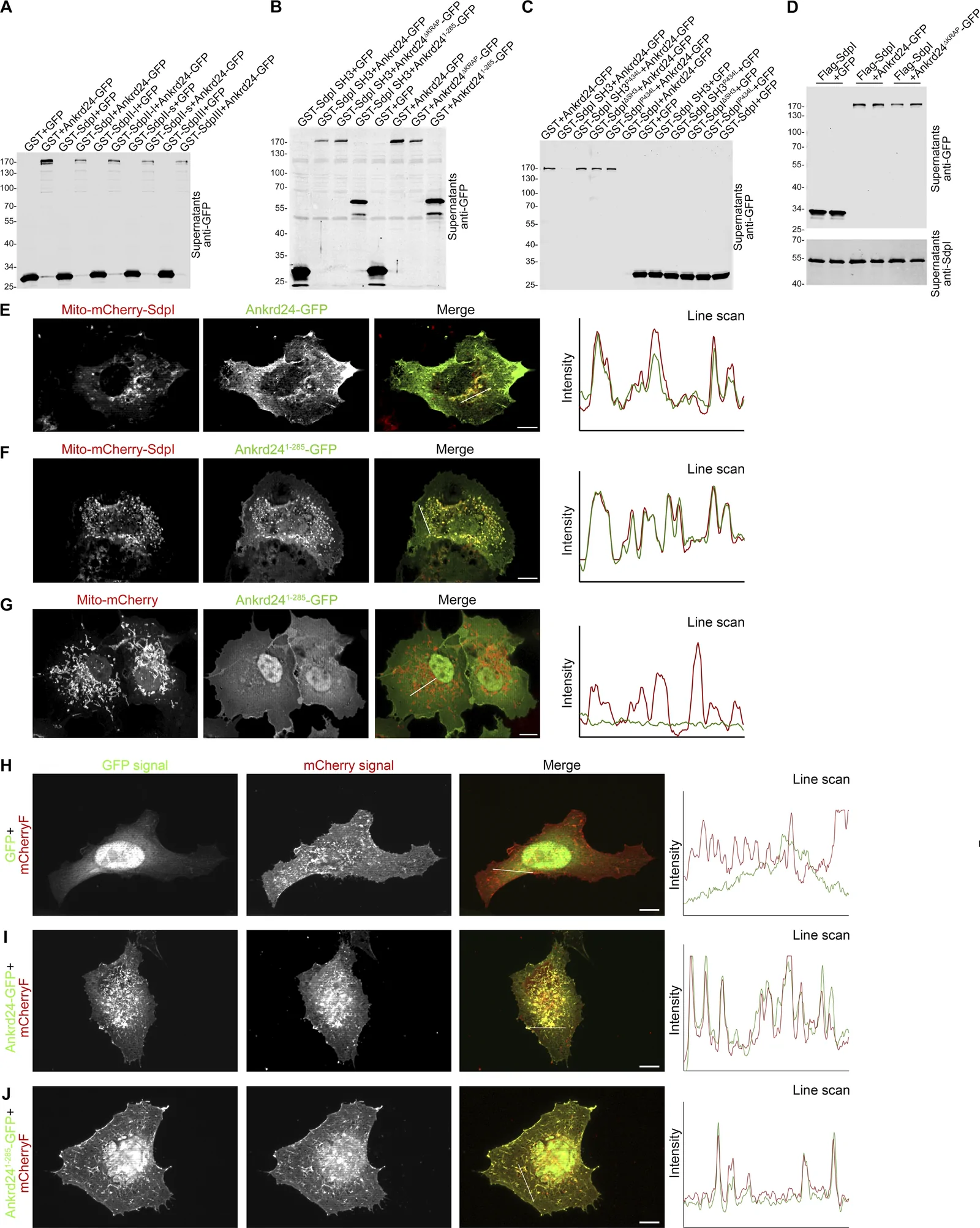
**Ankrd24 interacts with syndapin I and localizes to the plasma membrane. (A)** Supplementary supernatant data related to the coprecipitation experiments with lysates of HEK293 cells overexpressing GFP and Ankrd24-GFP and immobilized GST, GST-syndapin I (GST-SdpI), GST-syndapin II (GST-SdpII-s and GST-SdpII-l), and syndapin III (GST-SdpIII, respectively, shown in Fig. 1 B. **(B)** Supplementary supernatant data related to the coprecipitation experiments with lysates of L929 cells overexpressing GFP, Ankrd24-GFP, Ankrd24^ΔKRAP^-GFP and Ankrd24^1-285^-GFP, respectively, and immobilized GST-tagged syndapin I SH3 domain (GST-SdpI SH3) and GST, respectively, shown in Fig. 1 C. **(C)** Anti-GFP immunoblottings of supernatants corresponding to the coprecipitation experiments with lysates of L929 cells overexpressing either GFP or Ankrd24-GFP and immobilized GST, GST-tagged syndapin I SH3 domain (GST-SdpI SH3), the P434L mutant thereof (GST-SdpI SH3^P434L^), GST-syndapin I with a SH3 deletion (GST-SdpI^ΔSH3^), full-length syndapin I carrying a P434L point mutation (GST-SdpI^P434L^), and wild-type syndapin I (GST-SdpI), respectively, shown in Fig. 1 D. **(D)** Supplementary data related to the coimmunoprecipitation analyses of Flag-syndapin I (Flag-SdpI) with Ankrd24-GFP, Ankrd24^ΔKRAP^-GFP, and GFP, respectively (Fig. 1 E), showing the anti-GFP and anti–syndapin I (anti-SdpI) immunoblotting of supernatants. **(E–G)** Individual immunofluorescence channels of the subcellular recruitment assays in COS-7 cells shown in Fig. 1, G–I (merges are repeated for comparison) and lines scans (right panels) quantitatively assessing colocalizations (position indicated in the merges by white lines). The MIPs show a substantial spatial overlap of Ankrd24-GFP (E; green in merge) and Ankrd24^1-285^-GFP (F; green in merge) with mito-mCherry-SdpI (E and F; red in merges). Ankrd24^1-285^-GFP did not colocalize with mito-mCherry (G). Bars, 10 µm. **(H–J)** Ankrd24 localizes to the plasma membrane, as seen from individual fluorescence channels and merges of MIPs of GFP (H), Ankrd24-GFP (I) and Ankrd24^1-285^-GFP (J) (each shown in green in merge) in HeLa cells cotransfected with mCherryF (shown in red in merge) corresponding to Fig. 2, I–K and intensity line plots along the white lines depicted in the merged images. Ankrd24-GFP and Ankrd24^1-285^-GFP but not GFP exhibit a good spatial overlap at the plasma membrane with mCherryF. Bars, 10 µm. MIP, maximum intensity projection. Source data are available for this figure: SourceData FS1.

From cDNA prepared from brain of 8-wk-old mice, we cloned full-length *Mus musculus* Ankrd24 (NCBI Reference Sequence: XP_006514159.1; Ankrd24x5; 1041 aa), which represents one of the longest of the many splice variants of Ankrd24 predicted in mice in the NCBI database. In this form of Ankrd24, the +KRxAPxPP motif (KKRKAPQPP in mouse; aa 274–282) is located between the predicted N-terminal ankyrin repeats and the proposed C-terminal coiled domain of Ankrd24. Coprecipitation analyses showed that full-length Ankrd24 was indeed specifically coprecipitated with immobilized syndapin I (Qualmann et al., 1999) (Fig. 1 B). Ankrd24 also bound to immobilized muscle-enriched syndapin III (Seemann et al., 2017) and the two different splice isoforms of the more ubiquitously expressed syndapin isoform, syndapin II (Qualmann and Kelly 2000) (Fig. 1 B). The interaction was therefore conserved among all members of the syndapin family (Fig. 1 B and Fig. S1 A).

Further analyses showed that the interaction occurred with the N-terminal part of Ankrd24 and indeed critically required the identified KKRKAPQPP (KRAP) motif (Fig. 1 C and Fig. S1 B). P434L mutation of the syndapin I SH3 domain (Qualmann et al., 1999) confirmed that the interaction is SH3 domain mediated and based on a canonical SH3/PxxP interaction (Fig. 1 D and Fig. S1 C).

To further address the relevance of the identified syndapin I interaction with Ankrd24, we next conducted coimmunoprecipitations. Flag-tagged syndapin I specifically coimmunoprecipitated Ankrd24-GFP (Fig. 1 E and Fig. S1 D). Additional coimmunoprecipitation experiments with an Ankrd24 mutant lacking the KRAP motif (Ankrd24^ΔKRAP^; Ankrd24^Δ273-286^) demonstrated that the KRAP motif is required for Ankrd24/syndapin interactions (Fig. 1 E and Fig. S1 D).

Reconstitutions with purified proteins proved that the identified Ankrd24/syndapin I interaction was direct, as demonstrated by the specific detection of purified syndapin I in the eluates from immobilized GST-Ankrd24^1-285^ (Fig. 1 F).

Subcellular recruitment assays with mitochondrially anchored syndapin I (Kessels and Qualmann, 2002) proved that syndapin I/Ankrd24 complexes are formed in living cells. Both Ankrd24-GFP and Ankrd24^1-285^-GFP were specifically recruited to syndapin I–decorated mitochondria (Fig. 1, G and H; and Fig. S1, E and F). Control experiments demonstrated that the Ankrd24 recruitment was dependent on the presence of syndapin I at the mitochondrial surface (Fig. 1 I and Fig. S1 G).

### Ankrd24 is a membrane-binding N-Ank protein

**Figure 2.**
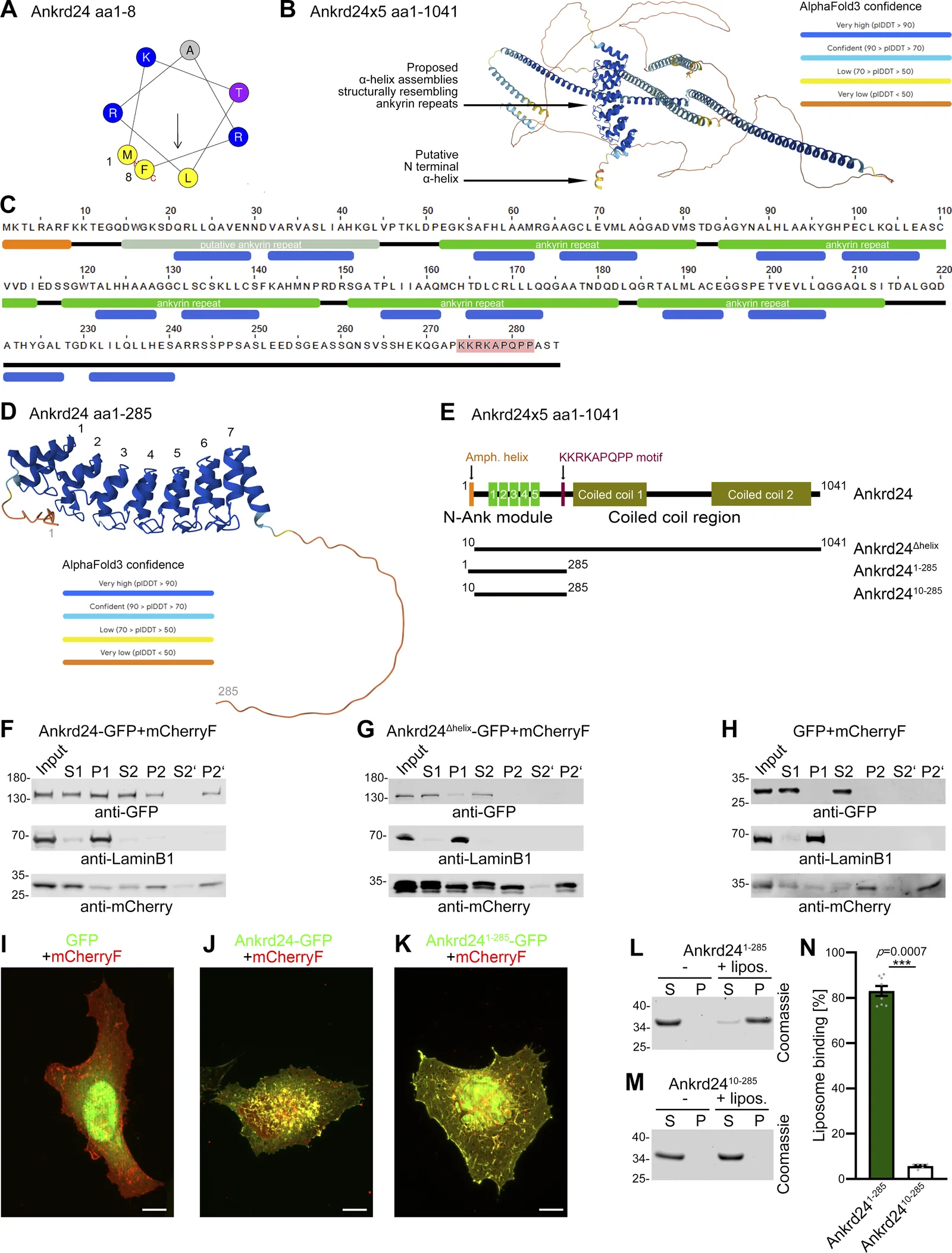
**Ankrd24 is a membrane-binding N-Ank protein. (A)** Helical wheel representation of the N terminus of mouse Ankrd24 highlighting its amphipathic nature (strongly hydrophobic residues, yellow; positively charged, very hydrophilic residues, blue; threonine [aa #3] in purple and alanine [aa #6] in gray). **(B)** AlphaFold3 representation of mouse full-length Ankrd24x5 (UniProt Q9UPS8; NCBI: XP_006514159.1). Note that the positions of the long helices in the C-terminal part of Ankrd24 are positioned differently from run to run, i.e., are in random orientation. The selected view is thus not representative for the overall structure of Ankrd24. **(C)** N-terminal aa sequence of mouse Ankrd24 with its N-terminal amphipathic helix (marked in orange), its five ankyrin repeats according to UniProt (green bars), the putative N-terminal ankyrin repeat with a low similarity to published ankyrin repeat consensi (light green-gray bar), the assemblies of each two short antiparallel α-helices proposed by AlphaFold3 (blue bars), which mostly overlap with the proposed ankyrin repeats, and the KRAP motif important for syndapin I binding (highlighted in rosé). **(D)** AlphaFold3 representation of Ankrd24^1-285^. Note that in the deletion context, the α-helix at the Ankrd24 N terminus was not predicted—an inconsistency in AlphaFold3 structure predictions when compared with full-length Ankrd24 (B). **(E)** Scheme of the domain structure of Ankrd24x5. N-Ank domain with N-terminal amphipathic helix (orange), 5 suggested ankyrin repeats (according to UniProt; Q9UPS8) (green), KKRKAPQPP motif (red), and two large separated regions predicted to represent coiled-coil domains (olive; prediction according to COILS; tool no longer available via Expasy). **(F–H)** Subcellular fractionations of lysates of Ankrd24-GFP, Ankrd24^10-1041^-GFP (Ankrd24^Δhelix^-GFP), and GFP-transfected HEK293 cells coexpressing mCherryF. mCherryF represents plasma membrane–integrated proteins; endogenous laminB1 represents nuclear proteins. Ankrd24-GFP (F) cofractionates with mCherryF in both P2 and P2′, whereas both Ankrd24^Δhelix^-GFP (G) and GFP (H) did not and remained in the supernatant fractions S1 and S2. **(I–K)** MIPs of GFP (I), Ankrd24-GFP (J), and Ankrd24^1-285^-GFP (K) (each shown in green) in HeLa cells cotransfected with mCherryF (shown in red). See Fig. S1 for individual fluorescence channels and for intensity line scans. Bars, 10 µm. **(L–N)** Representative example images of Coomassie-stained SDS-PAGE analyses of liposome coprecipitation studies (S, supernatant; P, pellet) addressing membrane binding of the recombinant, purified, untagged N-Ank module of Ankrd24 (Ankrd24^1-285^) (L) in comparison with a corresponding deletion mutant lacking the proposed N-terminal amphipathic helix (Ankrd24^10-285^) (M) and quantitative analysis thereof (N). Data, mean ± SEM (bar/dot plots with individual data points). *n* = 8 (Ankrd24^1-285^) and *n* = 6 (Ankrd24^10-285^) experiments each condition using three and four independent liposome preparations, respectively; Mann–Whitney. ***p < 0.001. The exact *p* value is reported directly in the figure. MIP, maximum intensity projection. For numerical source data, see Data S1. Source data are available for this figure: SourceData F2.

We proposed that Ankrd24 belongs to the recently identified N-Ank family of membrane-binding and -shaping proteins, as secondary structure predictions by Jpred4 and (PS)^2^-v2 suggested a helix N-terminal to the ankyrin repeats (Wolf et al., 2019). Helical wheel representations suggested that this N-terminal α-helix is amphipathic (Fig. 2 A). In line, structural predictions of full-length Ankrd24 by the AlphaFold3 server (Abramson et al., 2024) consistently but with low confidence also suggested an N-terminal α-helix (Fig. 2 B). AlphaFold3 furthermore predicted seven stacked pairs of antiparallel α-helices that resemble ankyrin repeats (Fig. 2 B). Only five of these are recognizable as ankyrin repeats from their sequence and thus listed in UniProt (Fig. 2 C, highlighted in green). The α-helix assembly located N-terminal of the 5 ankyrin repeats according to UniProt at least partially follows the Mosavi consensus for ankyrin repeats (Mosavi et al., 2002; Mosavi et al., 2004; Sedgwick and Smerdon, 1999) and may therefore be viewed as an additional putative ankyrin repeat (Fig. 2 C, light green-gray). In contrast, the additional C-terminal antiparallel α-helix structure predicted by AlphaFold3 fails to follow any key aspects of the Mosavi consensus or the Sedgwick and Smerdon consensus (Mosavi et al., 2004; Sedgwick and Smerdon, 1999). It may therefore rather represent an ankyrin array cap structure (Li et al., 2006) (Fig. 2 C).

AlphaFold3 furthermore predicts sets of rather extended α-helices that represent the coiled-coil regions proposed for N-Ank proteins (Wolf et al., 2019). They were predicted in random orientations (3 out of 3 runs) and are spaced apart by apparently disordered areas of low spatial confidence (Fig. 2 B).

When Ankrd24^1-285^ was analyzed, the single N-terminal α-helix spaced apart from the ankyrin repeat array was lacking (Fig. 2 D). It remained unclear why, as the proposed N terminus was in no contact with any other part in either the Ankrd24^1-285^ (Fig. 2 D) or the Ankrd24 full-length context (Fig. 2 B). AlphaFold3 in its current form is unable to consider membrane interfaces (Abramson et al., 2024), the membrane binding of Ankrd24, and its putative insertion into the membrane by the amphipathic helix could only be clarified by experimental work using Ankrd24’s N-Ank domain and deletion mutants lacking the putative amphipathic helix (Fig. 2 E). Subcellular fractionation studies of Ankrd24-GFP–expressing cells showed a presence of full-length Ankrd24-GFP in the membrane fractions P2 and P2’. Ankrd24 hereby behaved similar to the plasma membrane marker farnesylated monomeric Cherry (mCherryF) (Fig. 2 F), whereas an Ankrd24 mutant lacking the putative amphipathic helix (Ankrd24^Δhelix^-GFP), i.e., a mutation not affecting the N-terminal cap structure of the ankyrin repeat array (aa 21–41; Fig. 2 C), remained mostly in the fractions S1 and S2 and thereby behaved similar to GFP (Fig. 2, G and H).

Also in intact cells, Ankrd24-GFP specifically localized to mCherryF-marked plasma membrane folds, whereas GFP did not (Fig. 2, I and J; and Fig. S1, H and I). In line with the N-Ank hypothesis, the N-Ank module of Ankrd24 was sufficient for this observed membrane association (Fig. 2 K and Fig. S1 J).

Importantly, purified, recombinant Ankrd24^1-285^ liberated from the tag used for purification largely coprecipitated with liposomes in *in vitro* reconstitutions (Fig. 2 L). This finding demonstrated that the N-terminal domain of Ankrd24 (aa 1–285) was sufficient for membrane binding. Ankrd24 hereby required the N-terminal amphipathic helix because the truncated N-Ank module (Ankrd24^10-285^) showed almost no coprecipitation with liposomes (Fig. 2, L–N).

Thus, taken together, Ankrd24 is a plasma membrane–binding protein, and it associates with membranes directly, as proven by *in vitro* reconstitution using purified components. This identified molecular function critically required Ankrd24’s N terminus. The experimental evidence both in cells and *in vitro* establishes Ankrd24 as N-Ank superfamily member.

### In combination with the amphipathic helix, Ankrd24’s ankyrin repeats shape membranes

**Figure 3.**
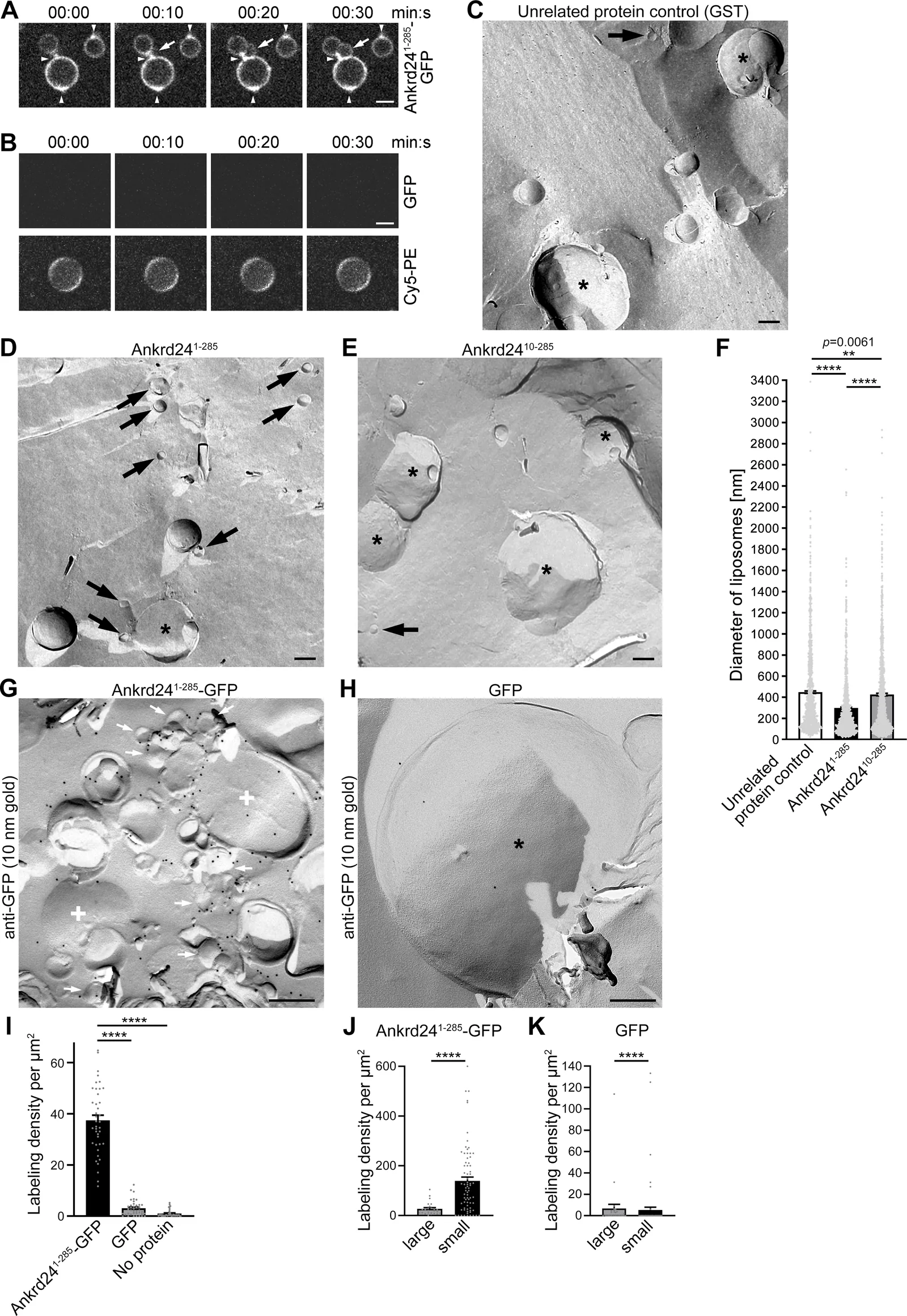
**Ankrd24’s N-Ank module shapes membrane topology. (A and B)** 3D live-spinning disc microscopy analyses of LUVs and GUVs incubated with purified, recombinant Ankrd24^1-285^-GFP (A) and GFP (B; dotation of the Folch lipids with Cy5-phosphatidylethanolamine [Cy5-PE] was used to label liposomes). Arrows mark Ankrd24^1-285^-GFP accumulations at a newly appearing liposome during the time window of analysis; arrowheads mark discrete areas of Ankrd24^1-285^-GFP accumulation at liposome surfaces, which, however, are too small to be resolved by light microscopy. Bars, 4 µm. See also Video 1. **(C–F)** Representative TEM images of freeze-fractured liposomes incubated with GST as unrelated protein control (C), Ankrd24^1-285^ (D), or Ankrd24^10-285^ (E) as well as quantitative analyses of mean liposome diameters (F). Note that Ankrd24^1-285^ shapes liposomes into smaller structures (arrows), whereas Ankrd24^10-285^ and unrelated protein failed to do so. Asterisks mark liposomes with large diameters (>500 nm). **(G and H)** TEM images of anti-GFP–labeled freeze-fracture replica of liposomes incubated with either purified, recombinant Ankrd24^1-285^-GFP (G) or GFP (H). **(I)** Quantitative assessments of anti-GFP immunogold labeling specificity. **(J and K)** Quantitative determinations of immunogold labeling densities at small (diameter ≤500 nm; arrows mark some examples) versus large liposomes (diameter >500 nm) after incubation with either Ankrd24^1-285^-GFP (J) or with GFP control protein (K). Note the high labeling density of Ankrd24^1-285^-GFP at small liposomes (J). Bars in C–E, G and H, 200 nm. Data, mean ± SEM and individual data point shown as bar/dot plots. F, *n* = 1,261 (unrelated protein control [GST]), *n* = 1,342 (Ankrd24^1-285^) and *n* = 1,328 (Ankrd24^10-285^) liposomes. I, *n* = 40 images each condition. J, *n* = 25 large and 75 small liposomes; K, *n* = 29 large and 72 small liposomes. Individual liposomes in I–K were from three independent experiments and liposome preparations each. Kruskal–Wallis/Dunn’s (F and I) and Mann–Whitney (J and K), respectively. **p < 0.01; ****p < 0.0001. For individual *p* value ≥0.0001, see figure. For numerical source data, see Data S1.

We next addressed Ankrd24’s putative membrane shaping ability by different *in vitro* reconstitutions (Fig. 3).

**Video 1.** **Movie related to the collection of MIPs of the 3D time-lapse imaging frames shown in Fig. 3 A.** Liposomes decorated with purified, recombinant Ankrd24^1-285^-GFP. For further details, please see Fig. 3 A. Shown are 8 frames (MIPs of 3D image stacks recorded every 10 s). The movie is shown with a speed of 2 frames/s. MIP, maximum intensity projection.

*In vitro* reconstitutions with giant or large unilamellar vesicles (GUVs and LUVs) and purified Ankrd24^1-285^-GFP directly visualized a specific accumulation of the N-Ank module of Ankrd24 at the surface of curved lipid surfaces in 3D live-imaging experiments using spinning disc microscopy (Fig. 3 A). Ankrd24^1-285^-GFP hereby localized to larger and highly curved liposomes as well as to liposome structures that were smaller (Fig. 3 A). The Ankrd24^1-285^-GFP association with liposomes was specific, as GFP was not recruited to liposomes (Fig. 3 B).

Transmission electron microscopy (TEM) studies of cryopreserved, freeze-fractured liposomes at ultrahigh resolution were used to address the hypothesis that Ankrd24’s membrane binding may shape liposomes by forcing membranes into stronger convex topologies, i.e., to smaller diameters. Strikingly, the diameters of liposomes incubated with Ankrd24^1-285^ were much smaller than those of controls incubated with an unrelated protein (Fig. 3, C and D). In contrast, such small liposomes were largely absent in incubations with the deletion mutant of the Ankrd24 N-Ank module lacking the amphipathic helix, Ankrd24^10-285^ (Fig. 3 E). Quantitative determinations demonstrated that the N-Ank module, Ankrd24^1-285^, shaped liposomes by highly significantly reducing the average size of liposomes when compared with GST or Ankrd24^10-285^ (Fig. 3 F).

We next visualized Ankrd24^1-285^-GFP at the surface of freeze-fractured liposomes by anti-GFP immunogold labeling (Fig. 3, G–I). The anti-GFP labeling was sensitive and highly specific (Fig. 3 I). In line with Ankrd24 promoting convex membrane surfaces, the labeling densities at small, i.e., strongly curved liposomes, were almost an order of magnitude higher than at large, i.e., more shallowly curved liposomes (139/µm^2^ vs. 27/µm^2^) (Fig. 3 J). This was not the case for the few immunogold labels obtained after incubation of liposome with GFP control protein, which showed a labeling density of only 5.3/µm^2^ at small liposomes and of 6.6/µm^2^ at large liposomes (Fig. 3 K). The Ankrd24^1-285^-GFP preference for strongly curved surfaces thus specifically reflected a property of Ankrd24^1-285^ (Fig. 3, G–K).

Overall, the investigation for the N-Ank properties of the N-Ank module of Ankrd24 revealed that it binds and shapes membranes.

### Ankrd24 gain-of-function promotes dendritic arborization in developing neurons

**Figure 4.**
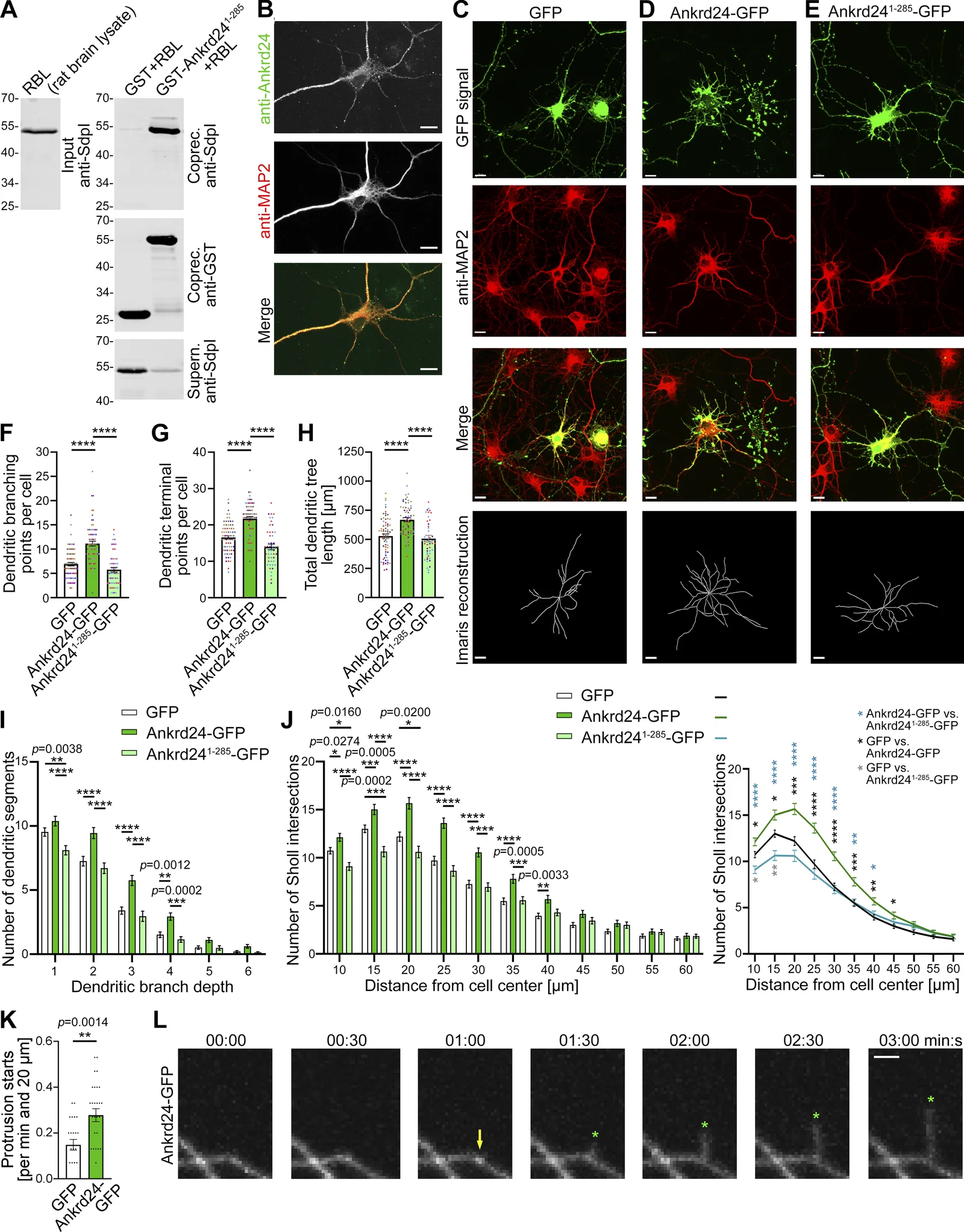
**Ankrd24 gain-of-function promotes the formation of the dendritic arbor in developing neurons. (A)** Immunoblot analyses of coprecipitation experiments with immobilized GST-Ankrd24^1-285^ and GST used as negative control with rat brain lysate (RBL) demonstrate that the KRAP motif–containing N terminus of Ankrd24 specifically coprecipitates endogenous syndapin I. **(B)** MIP of a primary hippocampal neuron at DIV6 immunostained for endogenous Ankrd24 and the dendritic marker MAP2. Bar, 10 µm. **(C–E)** Representative MIPs of primary rat hippocampal neurons that had been transfected at DIV4 with GFP (C), Ankrd24-GFP (D), and Ankrd24^1-285^-GFP (E), respectively, and were fixed about 40 h later. Lower panels show Imaris reconstructions of dendritic morphology (axon exclusion was validated by additional experiments, including anti-tau counterstainings; please note the resulting consistent quantitative functional data Fig. S2, A–E). Bars, 10 μm. **(F–J)** Quantitative determinations of dendritic branching points (F), of dendritic terminal points (G), of total dendritic tree length (H), and dendritic branch depth analyses (I), as well as Sholl analyses in both bar plots (including statistical significance calculations for each Sholl circle) and line plots (J). **(K)** Quantitation of the frequencies of dendritic protrusion starts in Ankrd24-GFP–expressing hippocampal neurons and GFP-expressing control cells observed in 3D time-lapse recordings. **(L)** 3D time-lapse imaging of a developing primary hippocampal neuron transfected with Ankrd24-GFP at DIV6 and imaged 16 h later using a spinning disc microscope. Green asterisks mark the tip of a forming dendritic branch. Yellow arrow marks an Ankrd24-GFP appearance prior to dendritic protrusion emergence. Bar, 2 µm. See also Videos 2 and 3. Data, mean ± SEM shown as bar plots (I and J left panel), line plot (J right panel), or bar/dot plots overlaid with individual data points (K) and with data points in different colors representing different sources of primary cultures (F–H; see Fig. S2, F–H for alternative presentations as SuperPlots). **(F–J)** GFP, *n* = 70; Ankrd24-GFP, *n* = 69; Ankrd24^1-285^-GFP, *n* = 48 neurons from four independent coverslips each condition and assay and from 5 (GFP, Ankrd24-GFP) and 3 (Ankrd24^1-285^-GFP) independent neuronal preparations used as source for the dissociated cell cultures transfected at DIV4. **(K)** GFP, *n* = 23; Ankrd24-GFP, *n* = 28 neurons (from 4 to 8 independent coverslips, respectively, and three independent neuronal preparations). Kruskal–Wallis/Dunn’s (F–H), two-way ANOVA/Sidak’s (I and J left panel), two-way ANOVA mixed-effects model (REML)/Tukey’s (J right panel), and Mann–Whitney (K). *p < 0.05; **p < 0.01; ***p < 0.001; ****p < 0.0001. For exact *p* values, please see Supplementary source data (Data S1) and/or figure; note that *p* values <0.0001 are not reported by Prism software. MIP, maximum intensity projection. For numerical source data, see Data S1. Source data are available for this figure: SourceData F4.

Our analyses have identified Ankrd24 as new binding partner for syndapin proteins (Fig. 1) and have revealed Ankrd24 to bind and shape membranes (Fig. 2 and Fig. 3). The N-Ank superfamily member ankycorbin was demonstrated to functionally co-operate with the highly neuronally expressed isoform syndapin I in early neuromorphogenesis (Izadi et al., 2023). To investigate whether such a function represents a specialty of ankycorbin, we analyzed in detail Ankrd24’s putative role in early neuronal development. In line with a putative function of Ankrd24 in the brain, immobilized recombinant GST-Ankrd24^1-285^ specifically coprecipitated endogenous syndapin I from rat brain lysate (Fig. 4 A).

**Video 2.** **Example movie related to the quantitative evaluation of dendritic arbor development in primary hippocampal neurons transfected with GFP shown in Fig. 4 K.** Shown is a 20 µm segment of a dendrite that undergoes dendritic branch induction during the time of observation shown (6 min). The movie is composed of 36 frames (MIPs of 3D image stacks recorded every 10 s) and shown with a speed of 5 frames/s. Note that one dendritic branch induction is occurring (1 in 6 min per 20 µm dendrite = 0.167/min per 20 µm dendrite). These data very well reflect the data shown in Fig. 4 K. MIP, maximum intensity projection.

**Video 3.** **Example movie related to the quantitative evaluation of dendritic arbor development in primary hippocampal neurons transfected with Ankrd24-GFP shown in Fig. 4 K.** Shown is a 20 µm segment of a dendrite that undergoes dendritic branch induction at two sites during the time of observation shown (6 min). The movie is composed of 36 frames (MIPs of 3D image stacks recorded every 10 s) and shown with a speed of 5 frames/s. Note that two dendritic branch inductions are occurring (2 in 6 min per 20 µm dendrite = 0.333/min per 20 µm dendrite). These data very well reflect the data shown in Fig. 4 K. Also note that the dendritic branch inductions in Ankrd24-GFP neurons are about twice as frequent than in corresponding control neurons (GFP-transfected; Video 2) (see also Fig. 4 K for quantitative data. MIP, maximum intensity projection.

**Figure S2.**
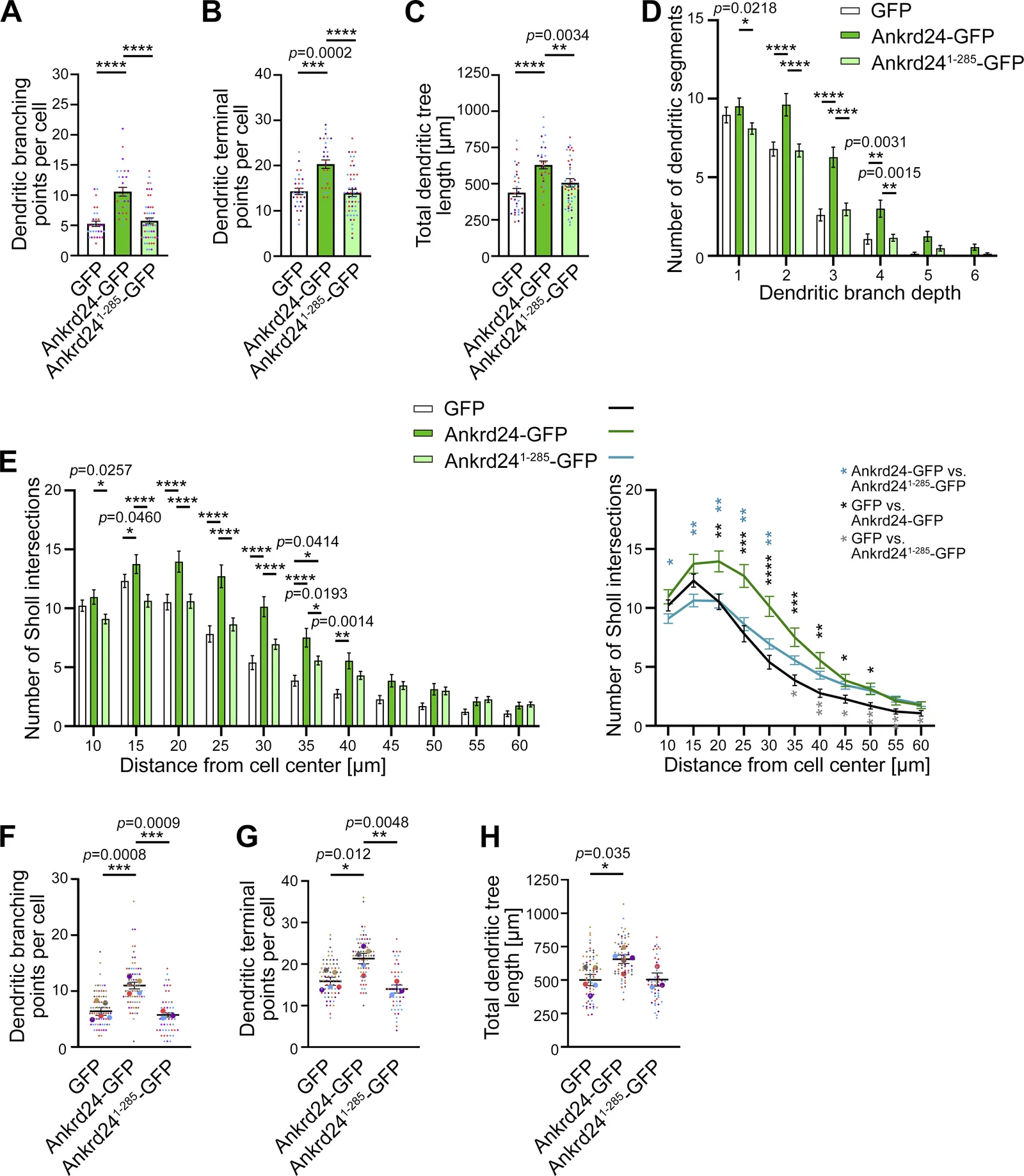
**Further validations of the Ankrd24 gain-of-function phenotypes promoting the formation of the dendritic arbor in developing neurons. (A–E)** Quantitative determinations of dendritic branching points (A), dendritic terminal points (B) and the total length of the dendritic arbor (C) as well as branch depth (D) and Sholl analyses (E) of dissociated E18 hippocampal rat neurons, transfected at DIV4 and analyzed 2 days later. The data shown are a subset of dendritic data shown in Fig. 4, F–J, for which axon exclusions were additionally validated by additional anti-tau counterstainings. **(F–H)** SuperPlots of the data shown in Fig. 4, F–H. Small dots, individual cellular data (color-coding refers to the different sources of primary cultures (assays); large dots, respective means of the different assays; black lines, means of these already averaged data sets. Data are mean ± SEM shown as bar plot (D and E left panel), line plots (E right panel), or bar/dot plots overlaid with the individual data points in different colors representing different sources of primary cultures (A–C) or SuperPlots (F–H). **(A–E)** GFP, *n* = 30; Ankrd24-GFP, *n* = 29; Ankrd24^1-285^, *n* = 48 neurons from four independent coverslips each condition and assay and from three independent neuronal preparations used as source for the dissociated cell cultures (assay). **(F–H)** Neuronal data as in Fig. 4, F–H (GFP, *n* = 70; Ankrd24-GFP, *n* = 69; Ankrd24^1-285^, *n* = 48 neurons from four independent coverslips each condition and assay) but number of independent neuronal preparations used as sources for the dissociated cell cultures considered as *n*. *n* = 5 (GFP, Ankrd24-GFP) and 3 (Ankrd24^1-285^-GFP). Kruskal–Wallis/Dunn’s (A–C), two-way ANOVA/Tukey’s (D and E left panel), two-way ANOVA mixed-effects model (REML)/Tukey’s (E right panel), and one-way ANOVA/Tukey’s (F–H), respectively. *p < 0.05; **p < 0.01; ***p < 0.001; ****p < 0.0001. For exact *p* values please see Supplementary Source data (Data S1) and/or figure; note that *p* values <0.0001 are not reported by Prism software. For numerical source data, see Data S1.

In primary hippocampal neurons, the dendritic compartment showed immunoreactivity for Ankrd24, as demonstrated by colocalization with the dendritic marker MAP2 (Fig. 4 B). Gain-of-function analyses showed that Ankrd24 is able to elicit dendritic arbor changes in neurons (Fig. 4, C–J, Fig. S2, A–E, and Fig. S2, F–H). Imaris software–based 3D reconstructions of neuronal morphologies at DIV6 showed a surplus of dendritic branching points and dendritic terminal points of Ankrd24-GFP–expressing primary hippocampal neurons when compared with GFP-expressing control neurons (Fig. 4, C, D, F, and G; and Fig. S2, A, B, F, and G). In addition, the total dendritic tree length was significantly increased (Fig. 4 H; and Fig. S2, C and H). Branch depth analyses demonstrated that Ankrd24 gain-of-function caused significant effects at branch depth levels 2, 3, and 4 when compared with developing GFP-expressing control neurons (Fig. 4 I and Fig. S2 D). Consistently, Sholl analyses also pointed out an increased complexity of the dendritic arbor of Ankrd24-overexpressing neurons (Fig. 4 J and Fig. S2 E). None of these Ankrd24 gain-of-function phenotypes were observed upon Ankrd24^1-285^-GFP overexpression, suggesting that multiple domains critically contribute to Ankrd24 functions (Fig. 4, E–J).

Quantitative analyses of 3D live-microscopy of developing neurons uncovered that Ankrd24 is responsible for the initiation of dendritic arborization. Ankrd24-GFP–expressing neurons showed about twice as many dendritic protrusion starts as control neurons (Fig. 4 K). In line with this function, Ankrd24 was present at nascent dendritic protrusion induction sites (Fig. 4 L).

### Ankrd24 deficiency leads to impaired dendritic arbor formation

**Figure 5.**
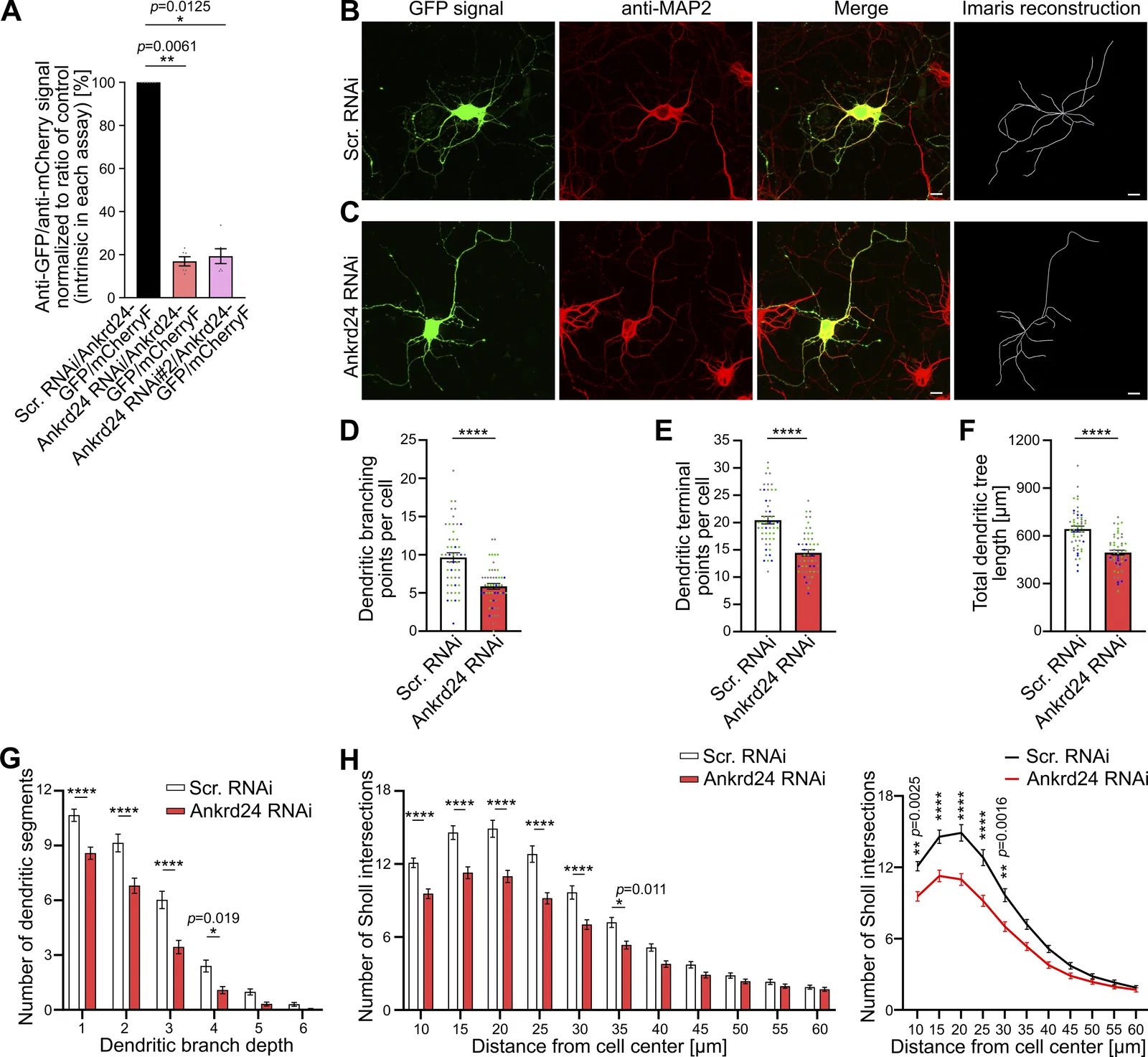
**Ankrd24 is required for proper dendritic arborization. (A)** Quantitation of fluorescence-based Western blot analyses of lysates of HEK293 cells transfected with plasmids driving the coexpression of two different shRNAi against rat/mouse Ankrd24 (Ankrd24 RNAi and Ankrd24 RNAi#2, respectively) or scr. RNAi together with both Ankrd24-GFP (knockdown target) and PM-mCherry, as independent reporter. The Ankrd24-GFP/PM-mCherry immunoblot signal ratios were determined and normalized to the Ankrd24-GFP/PM-mCherry ratio of the scr. RNAi condition. Both Ankrd24 RNAi and Ankrd24 RNAi#2 resulted in a significant reduction of Ankrd24-GFP expression. **(B and C)** Representative MIPs of primary rat hippocampal neurons transfected (at DIV4; fixed about 40 h later) with scr. RNAi coexpressing GFP (B) and Ankrd24 RNAi coexpressing GFP (C). Shown are anti-MAP2 immunostainings, GFP signals, merged signals, and 3D-reconstructions of neuronal morphologies by Imaris. Bars, 10 μm. **(D–H)** Quantitative determinations of specific defects in dendritic arborization by addressing dendritic branching points (D), dendritic terminal points (E), and the total length of the dendritic arbor (F) and by branch depth (G) and Sholl analyses as bar and line plots (H) (axon exclusion was additionally validated by anti-tau counterstainings, see Fig. S3, A–E). Data are mean ± SEM shown as bar plots (G and H left panel), line plots (H right panel), or bar/dot plots overlaid with individual data points (A) and with dots of different colors representing different sources of primary cultures (D–F; see Fig. S3, F–H for alternative presentations as SuperPlots). **(A)***n* = 6 biological replicates; (D–H) scr. RNAi, *n* = 50; Ankrd24 RNAi *n* = 50 neurons of  2–4 independent coverslips of each condition per assay and three independent neuronal preparations. Kruskal–Wallis/Dunn’s (A), unpaired, two-tailed Student’s *t* test (D–F), two-way ANOVA/Sidak’s (G), two-way ANOVA/Tukey’s (H left panel), and two-way ANOVA mixed-effects model (REML)/Sidak’s (H right panel). *p < 0.05; **p < 0.01; ****p < 0.0001; exact *p* values are provided directly in the figure panels; note that *p* values <0.0001 are not reported by Prism software. MIP, maximum intensity projection. For numerical source data, see Data S1.

To be able to address whether Ankrd24 is not only able to promote dendritic arborization in developing neurons but may also be critical for this biological process, we next established Ankrd24 RNAi. Quantitative, fluorescence-based western blot analyses demonstrated that 2 different Ankrd24 RNAi sequences even successfully suppressed the expression of Ankrd24 in a heterologous expression system, in which Ankrd24 was placed under a strong viral promoter (Fig. 5 A).

**Figure S3.**
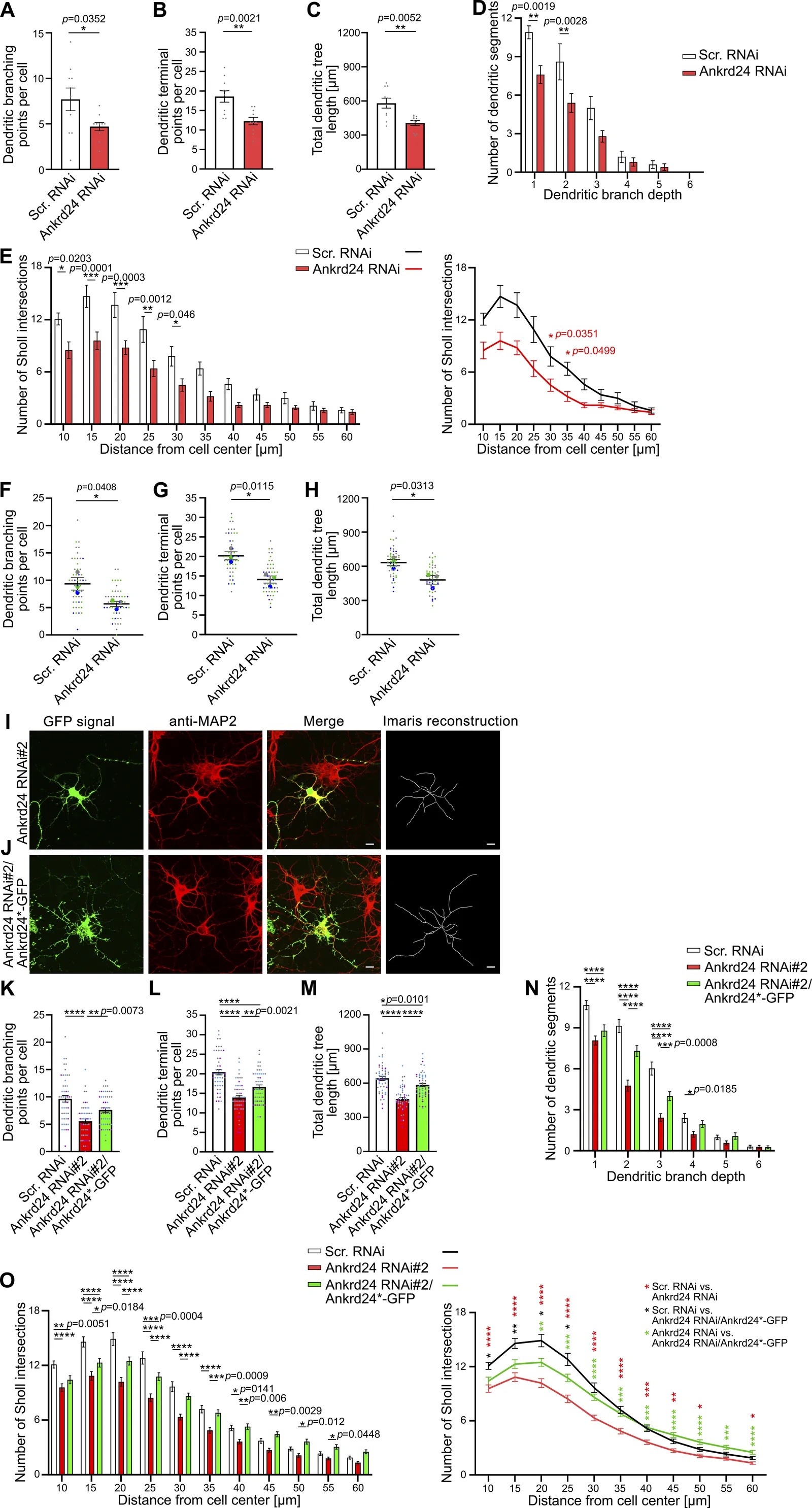
**Further validations of the identified Ankrd24 loss-of-function phenotypes on dendritic arborization by alternative analyses and employment of a second, independent RNAi. (A–E)** Quantitative determinations of dendritic branching points (A), dendritic terminal points (B) and the total length of the dendritic arbor (C) as well as branch depth (D) and Sholl analyses (E) of dissociated E18 hippocampal rat neurons, transfected at DIV4 and analyzed 2 days later. The data shown are a subset of data shown in Fig. 5, D–H, for which the axon exclusion was additionally validated by anti-tau counterstainings. **(F–H)** SuperPlots of the data shown in Fig. 5, D–F. Small dots, individual cellular data (color-coding refers to the different sources of primary cultures (assays); large dots, respective means of the different assays; black lines, means of these already averaged data sets. **(I–O)** Validations of the identified Ankrd24 loss-of-function phenotypes using a second, independent RNAi-targeting site and rescues by Ankrd24*-GFP reexpression in developing neurons. **(I and J)** Representative MIPs of primary rat hippocampal neurons transfected (at DIV4; fixed 40 h later) with scr. RNAi coexpressing GFP and Ankrd24 RNAi#2 coexpressing GFP (I) and Ankrd24 RNAi#2 coexpressing a silently mutated, Ankrd24 RNAi–insensitive, GFP-tagged Ankrd24 (Ankrd24 RNAi#2/Ankrd24*-GFP) (J). Shown are anti-MAP2 immunostainings, GFP signals, merged signals, and 3D-reconstructions of neuronal morphologies by Imaris. Bars, 10 μm. **(K–O)** Quantitative determinations of specific defects in dendritic arborization upon RNAi-mediated Ankrd24 deficiency and rescue effects by addressing dendritic branching points (K), dendritic terminal points (L), and the total length of the dendritic arbor (M), and by branch depth (N) and Sholl analyses (O). Data are mean ± SEM shown as bar plot (D and E left panel, N and O left panel), as line plots (E right panel, O right panel), as bar/dot plots overlaid with the individual data points in different colors representing different sources of primary cultures (A–C and K–M), or as SuperPlots (F–H). **(A–E)** Scr. RNAi and Ankrd24 RNAi#1, *n* = 10 neurons each (from four independent coverslips per each condition per assay [1]). **(F–H)** Neuronal data as in Fig. 5, D–F (scr. RNAi, *n* = 50; Ankrd24 RNAi *n* = 50 neurons of at least two independent coverslips of each condition per assay) but three independent neuronal preparations used as sources for the dissociated cell cultures considered as *n* (three each). **(K–O)** Scr. RNAi, *n* = 50 (corresponding to scr. RNAi data in Fig. 5, as conducted in joined multi-condition experiments); Ankrd24 RNAi#2, *n* = 52 and Ankrd24 RNAi#2/Ankrd24*-GFP, *n* = 52 neurons of  2–3 independent coverslips per each condition and assay and  three independent neuronal preparations. Unpaired, two-tailed Student’s *t* test (A, B, and F–H), Mann–Whitney (C), Kruskal–Wallis/Dunn’s (K), one-way ANOVA/Holm–Sidak (L and M), two-way ANOVA/Sidak’s (D and E left panel), two-way ANOVA/Tukey’s (N and O left panel), two-way RM ANOVA/Sidak’s (E right panel), and two-way ANOVA mixed effects model (REML)/Tukey’s (O right panel), respectively. *p < 0.05; **p < 0.01; ***p < 0.001; ****p < 0.0001. For exact *p* values please see Supplementary Source data (Data S1) and/or figure; note that *p* values <0.0001 are not reported by Prism software). MIP, maximum intensity projection. For numerical source data, see Data S1.

Rat primary hippocampal neurons transfected with Ankrd24 RNAi were marked by impaired dendritic arborization when compared with neurons expressing scrambled RNAi (scr. RNAi) (Fig. 5, B and C). Quantitative analyses of the dendritic arbor using 3D reconstructions by Imaris revealed that a lack of Ankrd24 led to significantly reduced numbers of dendritic branching points, dendritic terminal points, and also to a reduced total length of the dendritic tree (Fig. 5, D–F, Fig. S3, A–E, and Fig. S3, F–H). Dendritic branch depth (Fig. 5 G) and Sholl analyses (Fig. 5 H) demonstrated that the Ankrd24 loss-of-function effects identified occurred inside the entire dendritic arbor.

Ankrd24 loss-of-function phenotypes in dendritic arbor formation were also observed with a second, independent Ankrd24 RNAi tool, Ankrd24 RNAi#2, and thus were consistent between Ankrd24 RNAi and RNAi#2 (Fig. S3, I–O).

Importantly, the identified Ankrd24 loss-of-function phenotypes were opposite to the observed effects of Ankrd24 gain-of-function phenotypes (Fig. 4), i.e., both types of functional analyses were in line and pointed out that Ankrd24 plays a role in the dendritic arborization of developing neurons.

### Ankrd24 functions in dendritic arborization rely on Ankrd24’s syndapin-associating KRAP motif, N-terminal N-Ank module functionality and coiled-coil domain

**Figure 6.**
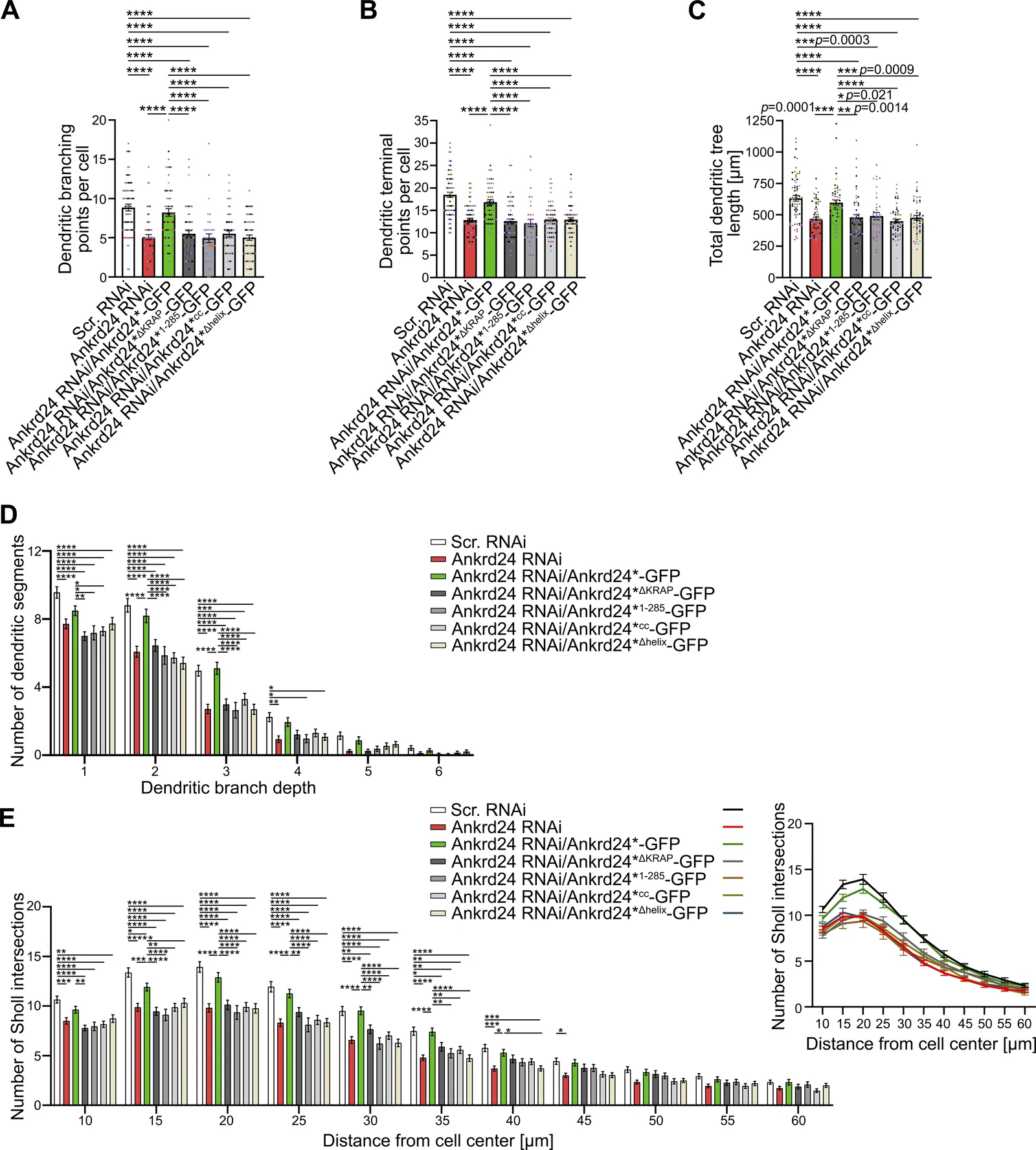
**Ankrd24 functions in dendritic arborization rely on Ankrd24’s syndapin-associating KRAP motif, N-terminal N-Ank module functionality, and coiled-coil domain. (A–E)** Quantitative determinations of the dendritic parameters dendritic branching points (A), dendritic terminal points (B), total dendritic tree length (C), number of dendritic segments per branch depth (D), and Sholl intersections (E) in the rescue attempts of Ankrd24 loss-of-function in primary hippocampal neurons with a variety of Ankrd24 mutants (axon exclusion was additionally validated by anti-tau counterstainings; see Fig. S5, A–E). Note that, in contrast to Ankrd24*-GFP, reexpression of Ankrd24*^ΔKRAP^-GFP, Ankrd24*^1-285^, Ankrd24*^cc^-GFP, and Ankrd24*^Δhelix^-GFP all failed to rescue the Ankrd24 loss-of-function phenotypes. Data are mean ± SEM shown as bar plots (D and E left panel), line plots (E right panel) and bar/dot plots overlaid with the individual data points in different colors representing different sources of primary cultures (A–C; see Fig. S5, F–H for alternative presentations as SuperPlots). **(A–E)** Scr. RNAi, *n* = 66; Ankrd24 RNAi, *n* = 64; Ankrd24 RNAi/Ankrd24*-GFP, *n* = 57; Ankrd24 RNAi/Ankrd24*^ΔKRAP^-GFP, *n* = 56; Ankrd24 RNAi/Ankrd24*^1-285^-GFP, *n* = 37, Ankrd24 RNAi/Ankrd24*^cc^-GFP, *n* = 55; and Ankrd24 RNAi/Ankrd24*^Δhelix^-GFP, *n* = 56 transfected neurons from 1 to 4 independent coverslips each condition and assay as well as 5 (scr. RNAi; Ankrd24 RNAi; Ankrd24 RNAi/Ankrd24*-GFP), 4 (Ankrd24 RNAi/Ankrd24*^ΔKRAP^-GFP; Ankrd24 RNAi/Ankrd24*^cc^-GFP; Ankrd24 RNAi/Ankrd24*^Δhelix^-GFP), and 3 (Ankrd24 RNAi/Ankrd24*^1-285^-GFP) independent neuronal preparations. One-way ANOVA/Tukey’s (A–C), two-way ANOVA/Tukey’s (D and E left panel), and two-way ANOVA mixed-effects model (REML)/Tukey’s (E right panel), respectively. *p < 0.05; **p < 0.01; ***p < 0.001; ****p < 0.0001 (for the multiple comparisons in E right panel, see Supplementary Source data, Data S1). For exact *p* values please see Supplementary Source data (Data S1) and/or figure. Note that *p* values <0.0001 are not reported by Prism software. For numerical source data, see Data S1.

**Figure S4.**
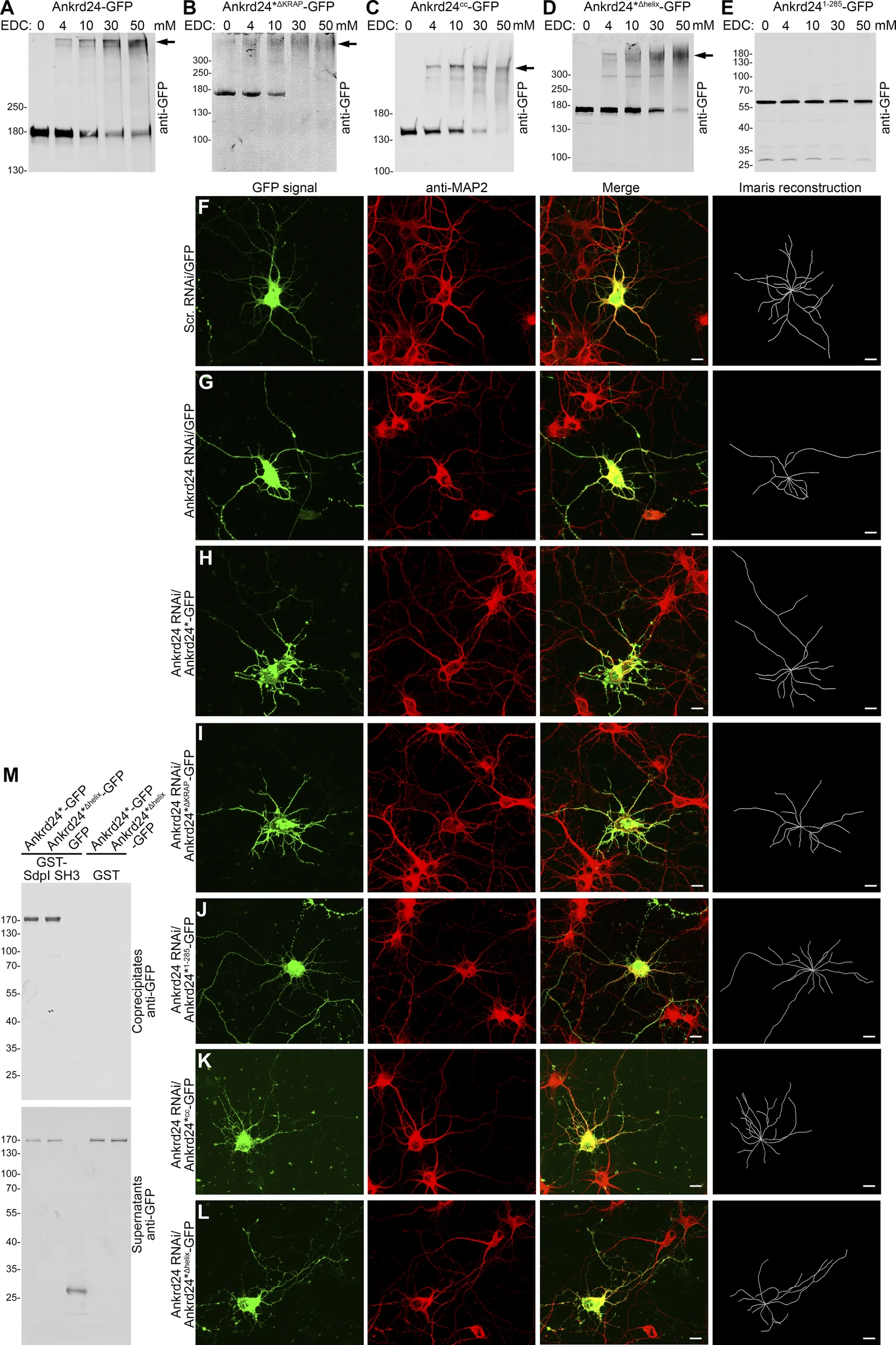
**Ankrd24 functions in dendritic arborization rely on Ankrd24’s syndapin-associating KRAP motif, N-terminal N-Ank module functionality, and its coiled-coil domain. (A–E)** Immunoblots of cross-linking experiments with increasing concentrations of the cross-linker EDC incubated with lysates of HEK293 cells overexpressing Ankrd24-GFP and mutants thereof. In the presence of the cross-linker, EDC, Ankrd24-GFP (A), Ankrd24*^ΔKRAP^-GFP (B), the predicted coiled region, Ankrd24^cc^-GFP (C), and Ankrd24*^Δhelix^-GFP (D) formed higher molecular weight complexes most likely representing dimers (arrows), whereas for Ankrd24^1-285^ (E) only a band of a molecular weight representing monomeric protein was observed. **(F–L)** Representative MIPs of primary rat hippocampal neurons transfected (at DIV4; fixed 40 h later) with scr. RNAi coexpressing GFP (F), Ankrd24 RNAi coexpressing GFP (G), Ankrd24 RNAi coexpressing silently mutated, Ankrd24 RNAi–insensitive Ankrd24-GFP (Ankrd24 RNAi/Ankrd24*-GFP; H), Ankrd24 RNAi coexpressing Ankrd24*^ΔKRAP^-GFP (Ankrd24 RNAi/Ankrd24*^ΔKRAP^-GFP; I), Ankrd24 RNAi coexpressing Ankrd24*^1-285^-GFP (Ankrd24 RNAi/Ankrd24*^1-285^-GFP; J), Ankrd24 RNAi coexpressing Ankrd24*^cc^-GFP (Ankrd24 RNAi/Ankrd24*^cc^-GFP; K), and Ankrd24 RNAi coexpressing Ankrd24*^Δhelix^-GFP (Ankrd24 RNAi/Ankrd24*^Δhelix^-GFP; L). Shown are GFP signals, anti-MAP2 immunostainings, and 3D reconstructions of neuronal morphologies by Imaris. Bars, 10 μm. **(M)** Anti-GFP immunoblotting analyses of coprecipitation analyses with immobilized GST and GST-syndapin I SH3 domain (GST-SdpI SH3) and lysates of L929 cells expressing Ankrd24*-GFP, Ankrd24*^Δhelix^-GFP, and GFP, respectively. MIP, maximum intensity projection; EDC, 1-ethyl-3-[dimethylaminopropyl]carbodiimide. Source data are available for this figure: SourceData FS4. For numerical source data, see Data S1.

**Figure S5.**
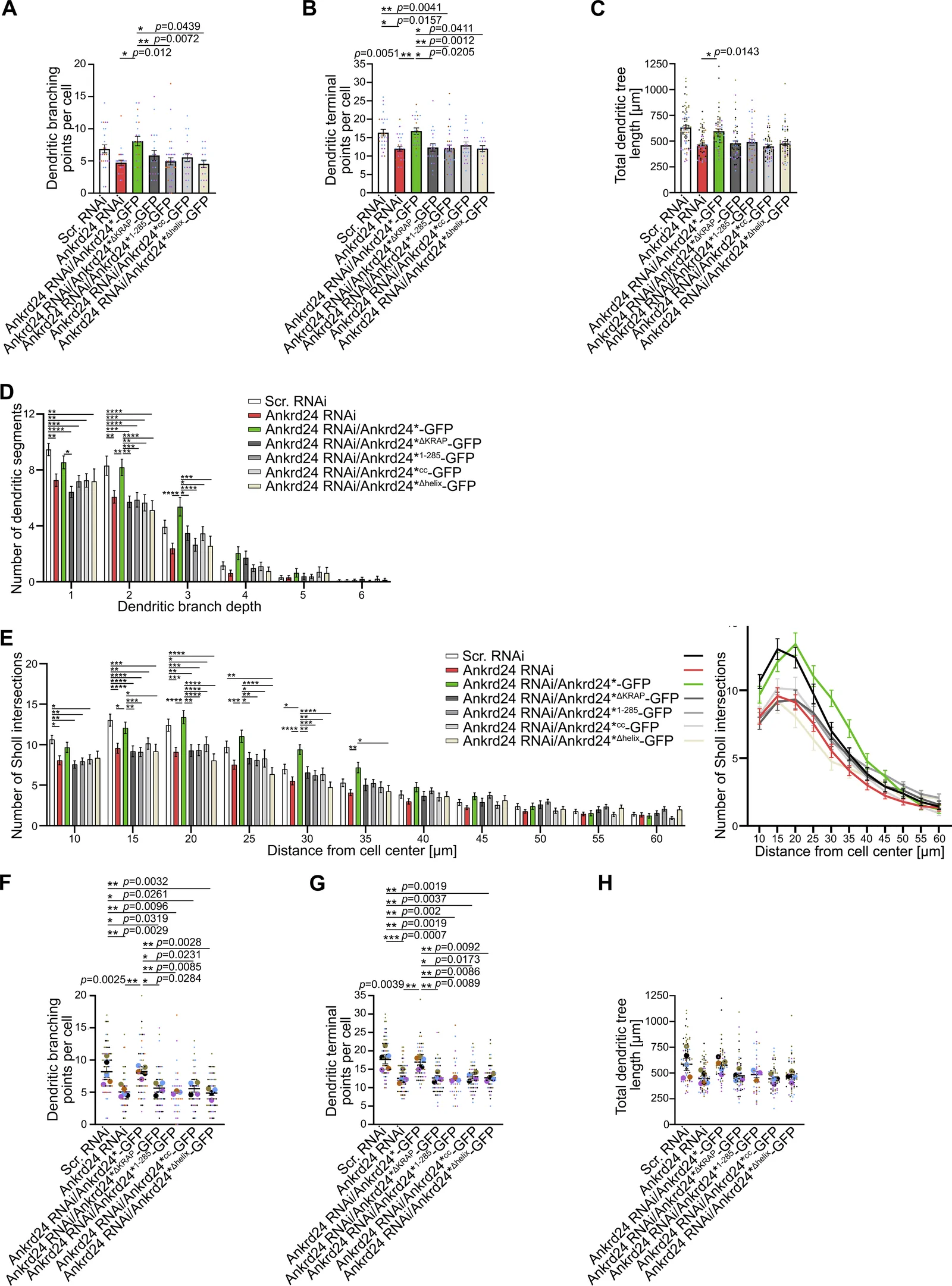
**Ankrd24 functions in dendritic arborization rely on Ankrd24’s syndapin-associating KRAP motif, N-terminal N-Ank module functionality, and coiled-coil domain (further validations and alternative visualizations of identified phenotypes by SuperPlots). (A–E)** Quantitative determinations of dendritic branching points (A), dendritic terminal points (B), the total length of the dendritic arbor (C), and the branch depth (D) as well as Sholl analyses (E) of dissociated E18 hippocampal rat neurons, transfected at DIV4 and analyzed 2 days later. The data shown are a subset of data in Fig. 6, A–E, for which the axon exclusion was additionally validated by anti-tau counterstainings. **(F–H)** SuperPlots of the data shown in Fig. 6, A–C. Small dots, individual cellular data (color-coding refers to the different sources of primary cultures (assays); large dots, respective means of the different assays; black lines, means of these already averaged data sets. Data are mean ± SEM shown as bar plot (D and E left panel) or as bar/dot plots overlaid with the individual data points in different colors representing different sources of primary cultures (A–C), line plots (E right panel), or as SuperPlots (F–H). **(A–E)** Scr. RNAi, *n* = 26; Ankrd24 RNAi, *n* = 26; Ankrd24 RNAi/Ankrd24*-GFP, *n* = 21; Ankrd24 RNAi/Ankrd24*^ΔKRAP^-GFP, *n* = 20; Ankrd24 RNAi/Ankrd24*^1-285^-GFP, *n* = 37; Ankrd24 RNAi/Ankrd24*^cc^-GFP, *n* = 19; and Ankrd24 RNAi/Ankrd24*^Δhelix^-GFP, *n* = 16 transfected neurons from 1 to 4 independent coverslips each per condition and assay as well as three independent neuronal preparations used as sources for the dissociated cell cultures used. **(F–H)** Data as in Fig. 6, A–C, (Scr. RNAi, *n* = 66; Ankrd24 RNAi, *n* = 64; Ankrd24 RNAi/Ankrd24*-GFP, *n* = 57; Ankrd24 RNAi/Ankrd24*^ΔKRAP^-GFP, *n* = 56; Ankrd24 RNAi/Ankrd24*^1-285^-GFP, *n* = 37; Ankrd24 RNAi/Ankrd24*^cc^-GFP, *n* = 55; and Ankrd24 RNAi/Ankrd24*^Δhelix^-GFP, *n* = 56 transfected neurons from 1 to 4 independent coverslips each per condition and assay) but the independent neuronal preparations used as sources for the dissociated cell cultures used considered as *n*. *n* = 5 (scr. RNAi; Ankrd24 RNAi; Ankrd24 RNAi/Ankrd24*-GFP), *n* = 4 (Ankrd24 RNAi/Ankrd24*^ΔKRAP^-GFP; Ankrd24 RNAi/Ankrd24*^cc^-GFP; Ankrd24 RNAi/Ankrd24*^Δhelix^-GFP), and *n* = 3 (Ankrd24 RNAi/Ankrd24*^1-285^-GFP). Kruskal–Wallis/Dunn’s (A–C), two-way ANOVA/Tukey’s (D and E left panel), two-way ANOVA mixed effects model (REML)/Tukey’s (E right panel), and one-way ANOVA/Tukey’s (F–H), respectively. *p < 0.05; **p < 0.01; ***p < 0.001; ****p < 0.0001 (for the multiple comparisons in E right panel, see Supplementary Source data, Data S1). For exact *p* values please see Supplementary Source data (Data S1) and/or figure; note that *p* values <0.0001 are not reported by Prism software. For numerical source data, see Data S1.

Rescue experiments with RNAi-resistant Ankrd24-GFP (Ankrd24*-GFP) proved the specificity of the identified Ankrd24 loss-of-function phenotypes in dendritic branching (Fig. S3, I–O) but also opened a research avenue towards vigorously addressing the putative importance of the different molecular mechanisms identified thus far (Fig. 6, A–E, Fig. S4, A–M, and Fig. S5, A–H). Resupplying Ankrd24-deficient neurons with RNAi-insensitive Ankrd24 (Ankrd24*-GFP) led to a rescue of the Ankrd24 RNAi and Ankrd24 RNAi#2-mediated loss-of-function phenotypes in dendritic branch points, dendritic terminal points, total dendritic tree length, as well as in dendritic branch depth 2 and 3 and in 15–40 µm intersection numbers of Sholl analyses (Fig. 6, A–E and Fig. S3, I–O), as the values of all of these parameters were statistically different from Ankrd24 RNAi– and Ankrd24 RNAi#2–expressing neurons, respectively.

Our studies have uncovered an interaction of Ankrd24 with syndapin proteins (Fig. 1) and furthermore identified Ankrd24 to be a membrane-binding and –shaping protein (Fig. 2 and Fig. 3). Both functions were mediated by the N-terminal part of Ankrd24. In line, a deletion mutant lacking this part and solely comprising the predicted coiled-coil regions mediating self-association (Fig. S4, A–E; Krey et al., 2022) (Ankrd24*^cc^-GFP) completely failed to rescue the Ankrd24 loss-of-function phenotypes in dendritic arbor formation. The dendritic branching points, the terminal points, and the total dendritic tree length of neurons reexpressing this mutant were indistinguishable from the values determined for Ankrd24-deficient neurons and significantly lower than those of control neurons expressing scr. RNAi (Fig. 6, A–E).

Dissecting the critical involvement of the identified syndapin binding to the KRAP motif of Ankrd24 by the use of an Ankrd24*^ΔKRAP^-GFP mutant and of the membrane binding and shaping via deletion of the critical amphipathic helix of the N-Ank modules of Ankrd24 (Ankrd24*^Δhelix^-GFP) revealed that both molecular mechanisms are essential for Ankrd24’s functions in dendritic arborization. In sharp contrast to the wild-type RNAi-resistant Ankrd24*-GFP, the dendritic parameters for neurons reexpressing the syndapin binding–incompetent mutant Ankrd24*^ΔKRAP^-GFP remained statistically indistinguishable from those of Ankrd24 RNAi neurons, i.e., showed no rescue (Fig. 6, A–C). Branch depth and Sholl analyses (Fig. 6, D and E) consistently demonstrated that Ankrd24*^ΔKRAP^-GFP was completely unable to rescue Ankrd24 loss-of-function phenotypes in dendritic arborization. The syndapin-binding site was thus critical for proper dendritic arbor formation.

Likewise, the membrane-binding- and -shaping-incompetent Ankrd24*^Δhelix^-GFP mutant also failed to support dendritic arborization. Deletion of solely the N-terminal 9 residues of Ankrd24 resulted in a complete failure to rescue the Ankrd24 loss-of-function phenotypes in dendritic arbor formation. The dendritic branching points, the terminal points, and the total dendritic tree length of neurons reexpressing these two mutants were indistinguishable from the values determined for Ankrd24-deficient neurons and significantly lower compared with those of control neurons expressing scr. RNAi (Fig. 6, A–E).

Interestingly, reexpression of a minimal membrane-shaping– and syndapin I-binding–competent portion of Ankrd24, Ankrd24*^1-285^-GFP, was also unable to rescue Ankrd24 loss-of-function phenotypes in dendritic branching (Fig. 6, A–E). This highlighted that, in addition to the membrane-shaping and syndapin I–binding portions of Ankrd24, also the coiled coil part is required for Ankrd24’s functions in dendritic arbor formation.

The inability of the mutants to rescue the Ankrd24 loss-of-function phenotypes hereby reflected dysfunctionalities, as all the mutants were expressed at levels reaching those of wild-type Ankrd24*-GFP (Fig. S4, H–L), were intact, as demonstrated by Western blot analyses (Fig. 1 C, Fig. 2 G, and Fig. S4, A–E) and furthermore remained functional for the non-targeted properties, such as membrane binding (Ankrd24^1-285^; Fig. 2), syndapin I binding (Ankrd24^1-285^, Ankrd24*^Δhelix^; Fig. 1 C and Fig. S4 M), and/or self-association (Ankrd24*^ΔKRAP^, Ankrd24*^cc^, and Ankrd24*^Δhelix^; Fig. S4, B–D).

Thus, besides the syndapin I–binding KRAP motif of Ankrd24, also Ankrd24’s membrane-binding and -shaping capabilities as well as the coiled-coil region of Ankrd24 are critical for Ankrd24 functions in dendritic arborization.

### Ankrd24 functions in neuromorphogenesis depend on syndapin I

**Figure 7.**
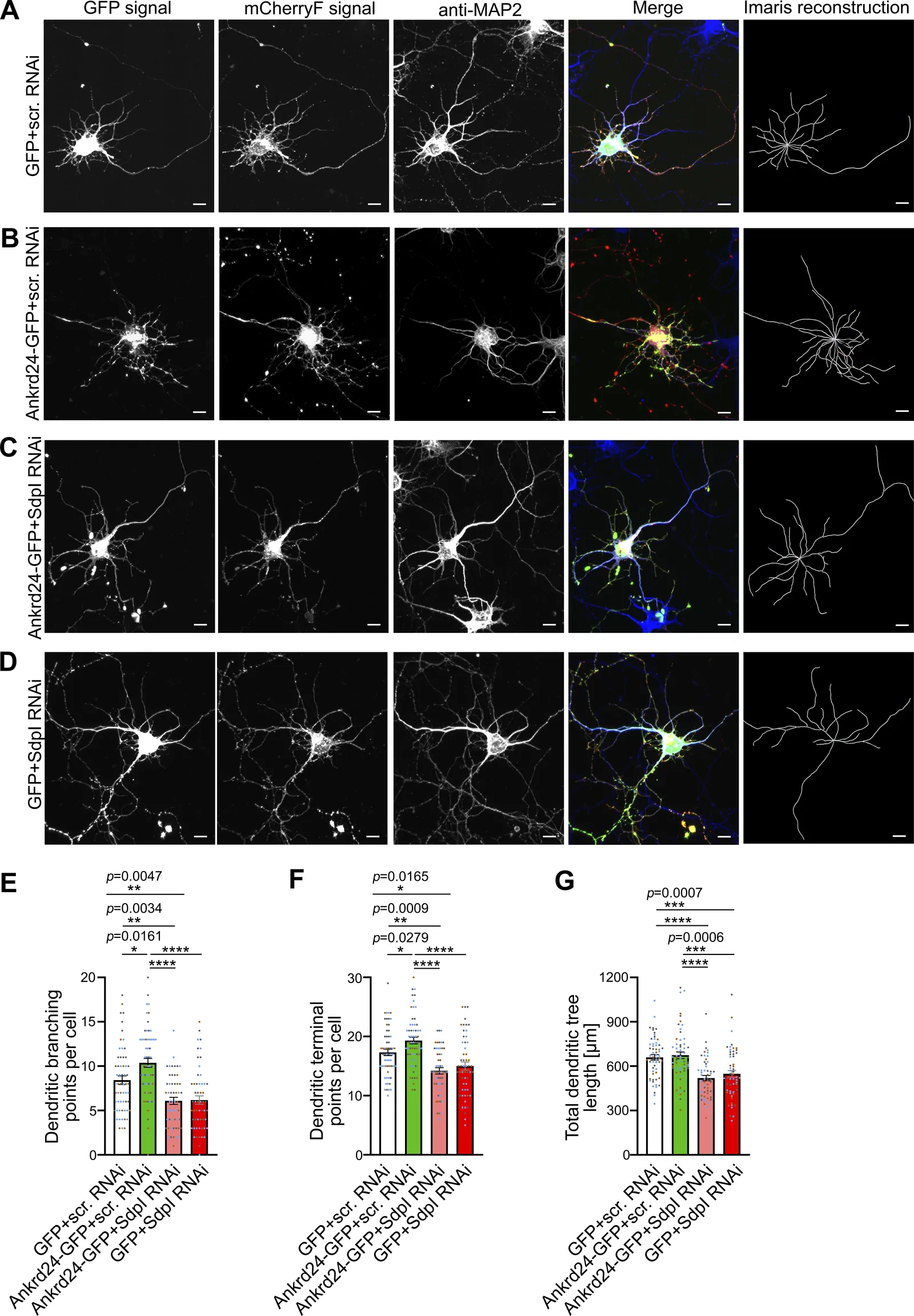
**Ankrd24’s functions in dendritic arbor formation critically rely on syndapin I. (A–D)** MIPs of anti-MAP2–labeled neurons that had been transfected with combinations of GFP and scr. RNAi (GFP+scr. RNAi; A), Ankrd24-GFP and scr. RNAi (Ankrd24-GFP+scr. RNAi; B), Ankrd24-GFP and syndapin I RNAi (Ankrd24-GFP+SdpI RNAi; C), as well as with GFP and syndapin I RNAi (GFP+SdpI RNAi; D) at DIV4 and fixed 2 d later and 3D reconstructions of neuronal morphology by Imaris software. The RNAi plasmids were reported by mCherryF coexpression. Scale bars, 10 μm. **(E–G)** Quantitative determinations of the key dendritic arborization parameters dendritic branching points (E), dendritic terminal points (F), and total length of the dendritic tree (axon exclusion was additionally validated by anti-tau counterstainings) (G). Data, mean ± SEM shown as bar/dot plots showing additionally the individual data points in different colors representing different sources of primary cultures. GFP+scr. RNAi, *n* = 59; Ankrd24-GFP+scr. RNAi, *n* = 59; Ankrd24-GFP+SdpI RNAi, *n* = 53; GFP+SdpI RNAi, *n* = 55 neurons of 2–4 independent coverslips each per condition and assay and three independent neuronal preparations. One-way ANOVA/Tukey’s (E), one-way ANOVA/Holm-Sidák's (F) and Kruskal–Wallis/Dunn’s (G). *p < 0.05; **p < 0.01; ***p < 0.001; ****p < 0.0001 (exact *p* values are provided directly in the figure panels; note that *p* values <0.0001 are not reported by Prism software). MIP, maximum intensity projection. For numerical source data, see Data S1.

Since the KRAP motif was critical for Ankrd24’s functions in dendritic arborization, we explicitly addressed whether this represents a putative requirement of syndapin I for Ankrd24’s functions (Fig. 7, A–G). Syndapin I is the major syndapin isoform in the nervous system (Qualmann et al., 1999). Despite usually weaker phenotypes in coexpression paradigms and in line with the single transfections of Ankrd24 described above (Fig. 4), also primary hippocampal neurons double transfected with Ankrd24-GFP and (mCherryF reported) scr. RNAi plasmids showed significant increases in dendritic arborization compared with GFP-transfected control neurons (Fig. 7 A). Strikingly, RNAi-mediated knockdown of syndapin I expression completely disrupted Ankrd24-mediated dendritic arborization, as all quantitative dendritic parameters of Ankrd24-GFP and syndapin I RNAi–coexpressing neurons were strongly diminished to values even below control levels (Fig. 7, C–G). In fact, Ankrd24-GFP and syndapin I RNAi–coexpressing neurons displayed values of all parameters that were similar to those determined for GFP and syndapin I RNAi–expressing neurons exhibiting a loss-of-function phenotype on dendritic arborization (Fig. 7, C–G). Therefore, the values represented a full suppression of the dendritic branch point and terminal point increases observed upon Ankrd24 gain-of-function by syndapin I deficiency. These data, therefore, allow the firm conclusion that Ankrd24-mediated dendritic branching was fully dependent on Ankrd24’s binding partner syndapin I.

### Ankrd24 and syndapin I interact at liposome membrane surfaces

**Figure 8.**
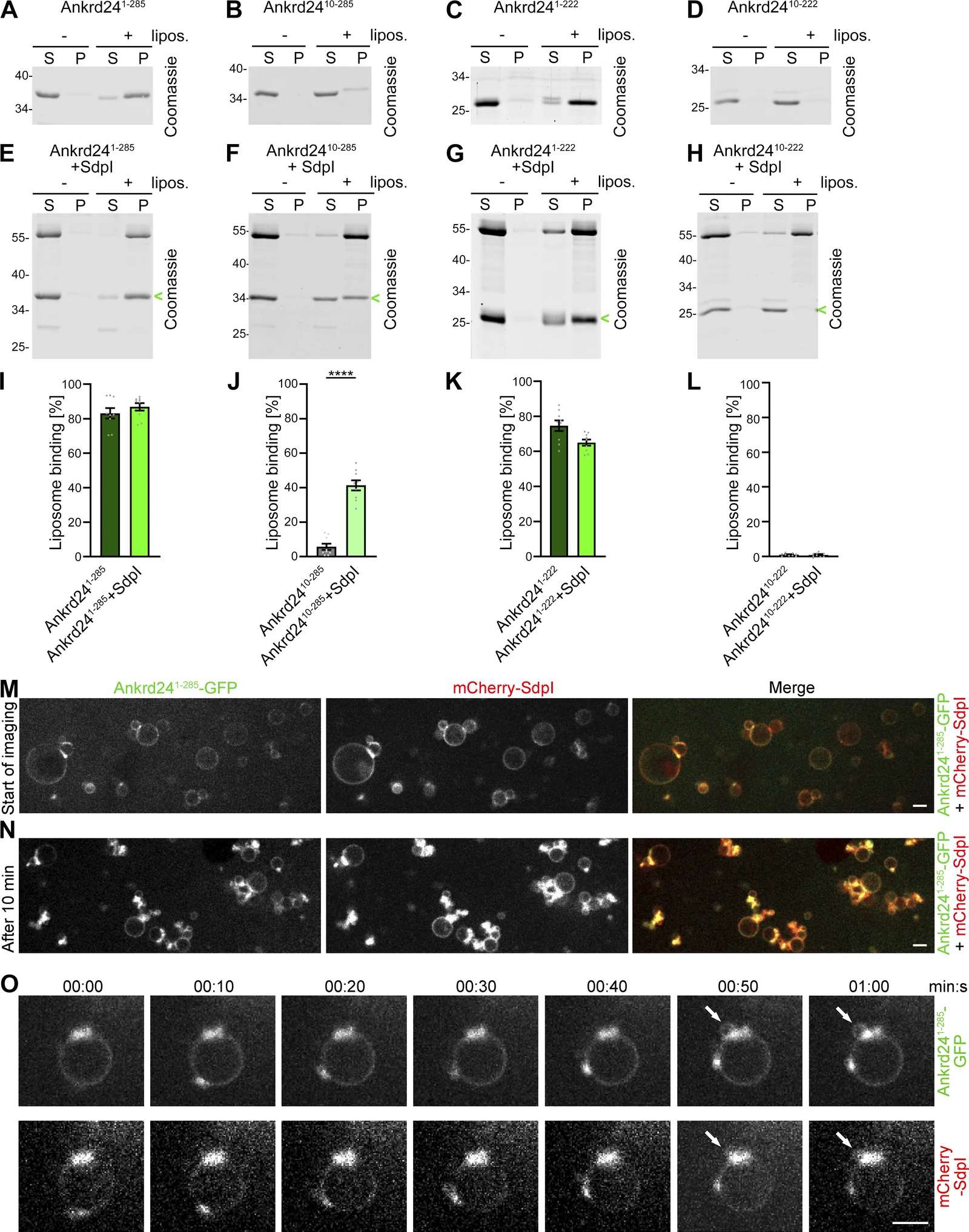
**Ankrd24 and syndapin I associate at liposome membrane surfaces. (A–L)** Liposome binding assays of recombinant purified Ankrd24^1-285^ (A and E), Ankrd24^10-285^ (B and F), Ankrd24^1-222^ (C and G), and Ankrd24^10-222^ (D and H) individually (A–D) or in combination with purified syndapin I (E–H) evaluated by Coomassie staining of SDS-PAGE gels (A–H) and quantitative analyses thereof (I–L). S, supernatant; P, pellet. Note that whereas Ankrd24^10-285^ and Ankrd24^10-222^ were unable to bind to liposomes on their own (B and D), syndapin I (55 kD band in E–H) recruited Ankrd24^10-285^ but not Ankrd24^10-222^ to membranes (F and H). Green < (E–H) mark the different Ankrd24 proteins examined. **(M and N)** MIPs of liposomes incubated with Ankrd24^1-285^-GFP and syndapin I-mCherry at the start of imaging (M) and about 10 min later (N). **(O)** MIPs of 3D live-spinning disc microscopy analyses of reconstitutions of liposomes incubated with purified, recombinant Ankrd24^1-285^-GFP and mCherry-syndapin I. Arrows highlight a small liposomal structure emerging from a site of a larger liposome that is marked by Ankrd24^1-285^-GFP and mCherry-syndapin I coaccumulation. Bars (M–O), 5 µm. Data, mean ± SEM (bar/dot plots with individual data points). Samples with Ankrd24^1-285^ and Ankrd24^10-285^, *n* = 9 independent reconstitution experiments using four independent liposome preparations; samples with Ankrd24^1-222^ or Ankrd24^10-222^, *n* = 9 independent reconstitution experiments using three independent liposome preparations. Mann–Whitney (I and J) and unpaired, two-tailed Student’s *t* test (K and L), respectively. ****p < 0.0001 (note that exact *p* values <0.0001 are not reported by Prism software). MIP, maximum intensity projection. Source data are available for this figure: SourceData F8. For numerical source data, see Data S1.

Our data showed that the function of Ankrd24 in neuromorphogenesis critically relies not only on the presence of the syndapin I–binding interface, the KRAP motif (Fig. 6), but specifically on the presence of syndapin I (Fig. 7). Besides syndapins (Itoh et al., 2005; Dharmalingam et al., 2009; Wang et al., 2009; Rao et al., 2010; Bai et al., 2012; Izadi et al., 2023), we observed that also the N-Ank protein Ankrd24 exhibits membrane binding and shaping capabilities (Fig. 2 and Fig. 3). To obtain molecular mechanistic insights into a putative functional cooperation of the two proteins, we first addressed in liposome-binding assays whether syndapin I would contribute to or compete with membrane association of Ankrd24 (Fig. 8, A–L). Quantitative analyses demonstrated that the membrane association of Ankrd24 was not negatively influenced by its binding partner syndapin I. Purified Ankrd24^1-285^ and syndapin I associated efficiently with liposomes in combinatory experiments (Fig. 8, A, E, and I).

Deletion of the N-terminal helix critical for membrane binding demonstrated that membrane-bound syndapin I can recruit Ankrd24. Ankrd24^10-285^, which did not show considerable membrane binding on its own, was observed to coprecipitate with liposomes to a highly significantly increased amount when syndapin I was present (Fig. 8, B, F, and J).

Additional experiments confirmed that this does not represent indirect effects but that the observed recruitment indeed required direct association of Ankrd24 with syndapin I. The increase of membrane-binding mediated by syndapin presence observed for Ankrd24^10-285^ was not detectable when a C-terminally truncated version lacking the syndapin I–binding site, i.e., the KRAP motif at aa 274–282, (Ankrd24^10-222^) was used. Ankrd24^10-222^ did not coprecipitate with liposomes irrespective of whether syndapin I was absent or present (Fig. 8, C, D, G, H, K, and L).

Together, these experiments showed that syndapin I and Ankrd24 interact at membrane surfaces and that syndapin I is able to recruit Ankrd24 in a KRAP motif–mediated manner to membrane surfaces.

Consistently, Ankrd24 and syndapin I coaccumulated at liposome surfaces in visual inspections. Purified Ankrd24^1-285^-GFP and mCherry-syndapin I added to liposomes showed that both proteins largely colocalized at liposome surfaces and showed no transient spatial segregation to individual liposomes or distinct liposome subareas but instead largely colocalized (Fig. 8, M and N).

Strikingly, the intensity of both proteins was highest at small liposomal structures. These small Ankrd24^1-285^-GFP– and mCherry-syndapin I–positive structures also appeared to be more abundant during later times in the incubations (Fig. 8, M and N).

3D time-lapse analyses using spinning disc microscopy demonstrated that Ankrd24^1-285^-GFP and mCherry-syndapin I indeed often coenrich at particular sites on the liposome surface (Fig. 8 O).

### In the presence of syndapin I, Ankrd24^1-285^-mediated membrane shaping requires coordination by Ankrd24^1-285^/syndapin I complex formation

**Figure 9.**
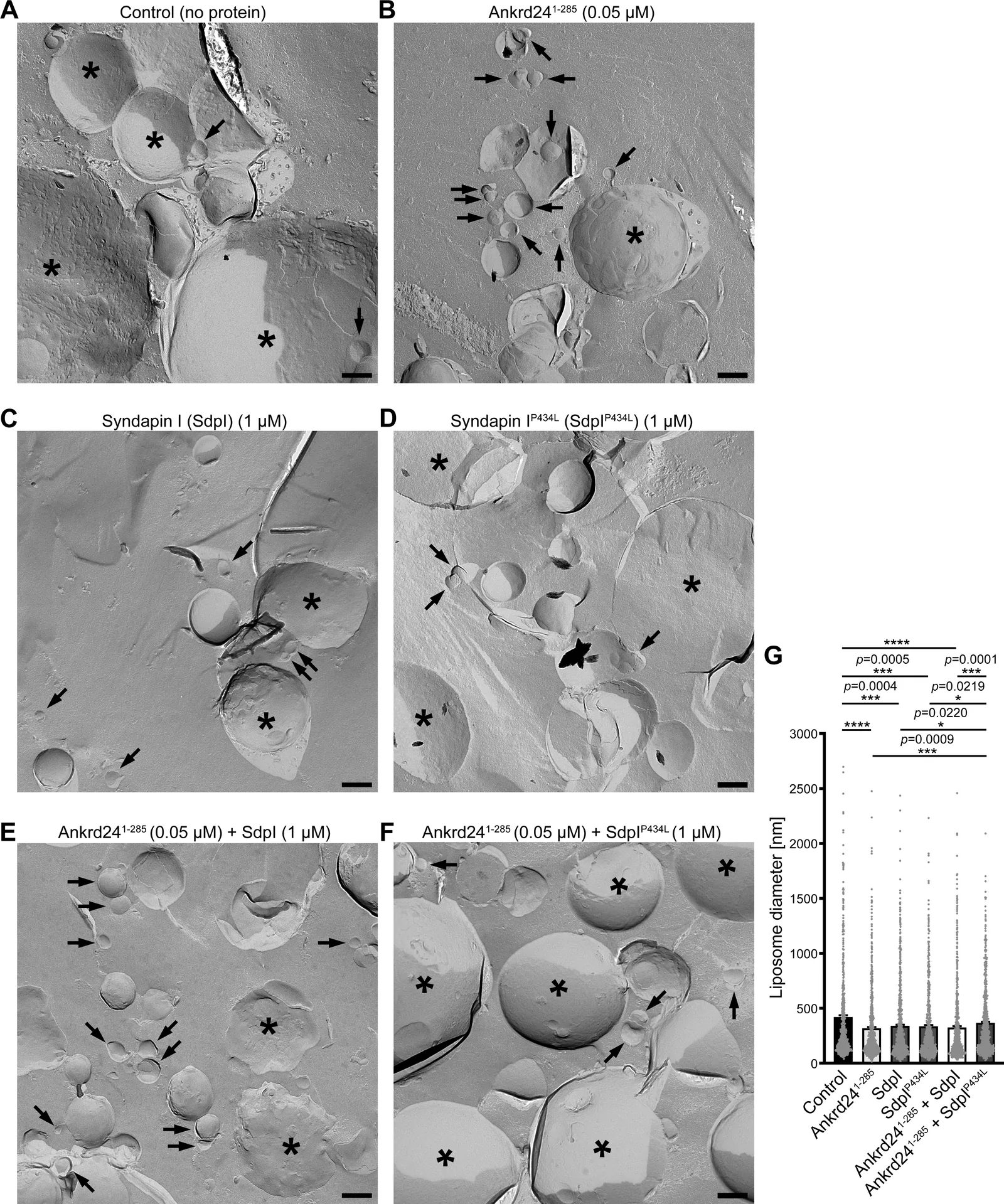
**In the presence of syndapin I, Ankrd24** ^**1-285**^ **-mediated membrane shaping requires coordination by Ankrd24** ^**1-285**^ **/syndapin I complex formation. (A–G)** TEM images of freeze-fractured, platinum-, and carbon-shadowed liposomes that were incubated with the indicated proteins and protein combinations or with merely buffer (control) for 30 min (A–F) and quantitative determinations of resulting liposome diameters (G). Large liposomes (diameter above mean of control; *) are marked along with examples for (very) small liposomes (diameter ≤200 nm; arrows). Bars, 200 nm. Data, mean ± SEM, and individual data points. **(G)** Control, *n* = 629 liposomes; Ankrd24^1-285^, *n* = 838 liposomes; SdpI, *n* = 842 liposomes; SdpI^P434L^, *n* = 801 liposomes; Ankrd24^1-285^ + SdpI, 813 liposomes; Ankrd24^1-285^ + SdpI^P434L^, *n* = 847 liposomes (from 42 images and 3 independent assays each). Statistical analysis, Kruskal–Wallis/Dunn’s. *p < 0.05; ***p < 0.001 with exact *p* values reported in the figure. ****p < 0.0001 (note that exact *p* values <0.0001 are not reported by Prism software). For numerical source data, see Data S1.

Analyses of putative cooperative, coordinated or competing effects of complex formation between syndapin I and Ankrd24 on their respective membrane-shaping functions required high-resolution studies by electron microscopy and quantitative analyses (Fig. 9, A–G). In line with our previous observations (Fig. 3) Ankrd24^1-285^—even when used in relatively low concentrations—led to smaller liposomes than control incubations (Fig. 9, A, B, and G). In line with previous observations (Izadi et al., 2023), incubations with syndapin I also gave rise to highly statistically significantly smaller liposomes when compared with control (buffer) incubations (Fig. 9, A, C, and G). The same was observed upon incubation with a syndapin I mutant harboring a point mutant impairing SH3 domain interactions (SdpI^P434L^; Qualmann et al., 1999) (Fig. 9, A, D, and G). Addition of wild-type syndapin I did not disturb the membrane shaping by Ankrd24^1-285^ (Fig. 9, E and G).

Strikingly, this was only true as long as Ankrd24/syndapin I complex formation was possible. When the ability to form complexes with Ankrd24 was disrupted by introducing a point mutation into syndapin I’s SH3 domain, this lack of coordinated action led to significantly diminished membrane shaping (Fig. 9, F and G; combination Ankrd24^1-285^+SdpI^P434L^ vs. Ankrd24^1-285^+SdpI, ***p = 0.0001). The impairments of coordinated Ankrd24+syndapin I action were so severe that the liposomes resulting from incubations with Ankrd24^1-285^+syndapin I^P434L^ were statistically indistinguishable from control liposomes (Fig. 9 G).

Together with the functional data highlighting the requirement of both the syndapin-binding KRAP motif of Ankrd24 (Fig. 6) and of syndapin I specifically (Fig. 7) in the identified role of Ankrd24 in dendritic arbor development, our data suggest a cooperative mechanism of Ankrd24 and syndapin I in membrane topology modulations and demonstrate that these functions rely on Ankrd24/syndapin I complex formation by the syndapin I SH3 domain.

### Ankrd24 functions in dendritic arborization critically rely on the syndapin I–binding partner Cobl-like

**Figure 10.**
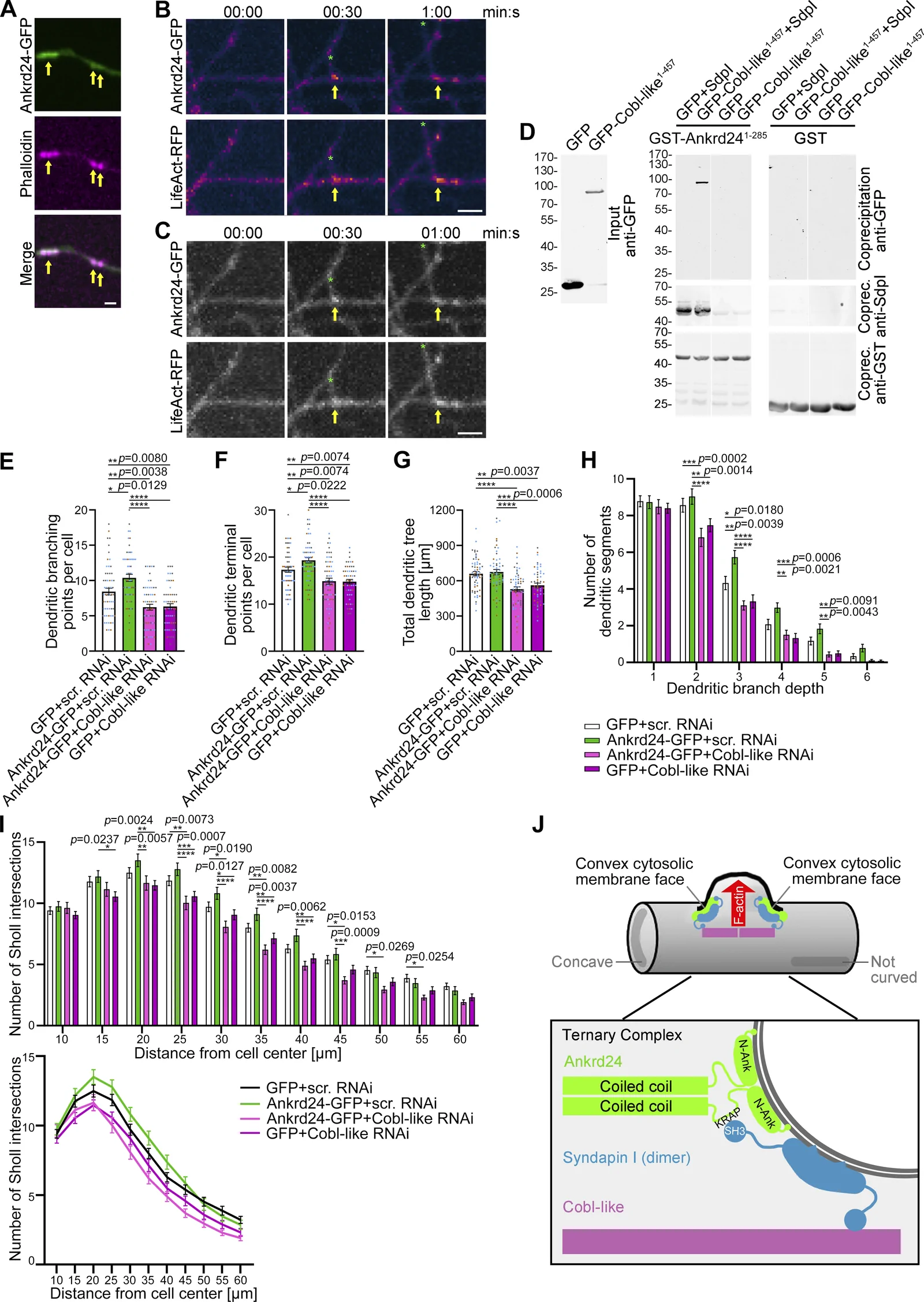
**Ankrd24 functions in dendritic arbor formation rely on the syndapin I–binding partner Cobl-like. (A)** MIP of a dendritic segment of a primary hippocampal neuron transfected with Ankrd24-GFP at DIV6, fixed 16 h later, and costained with fluorescently labeled phalloidin. Yellow arrows mark sites of Ankrd24-GFP and F-actin colocalization. Bar, 2 µm. **(B and C)** MIPs of 3D time-lapse imaging of a developing neuron transfected with Ankrd24-GFP and LifeAct-RFP at DIV6 and imaged 16 h later using a spinning disc microscope. False color representation of intensities (gem) (B) and original data (C). Yellow arrows mark sites of Ankrd24-GFP and F-actin colocalization at dendritic protrusion induction sites. The tip of the newly growing dendritic protrusion is marked by green asterisks for better visibility. Bar, 2 µm. **(D)** Immunoblotting analyses of cell lysates (input) and eluates (coprecipitation) of coprecipitation experiments uncovering specific and syndapin I–dependent formation of complexes composed of immobilized GST-Ankrd24^1-285^, syndapin I, and GFP-Cobl-like^1-457^ and corresponding control experiments with immobilized GST or replacing lysates of HEK293 cells expressing GFP-Cobl-like^1-457^ with those expressing merely GFP. **(E–I)** Quantitative determinations of dendritic branching points (E), of dendritic terminal points (F), of total dendritic length (G) and dendritic branch depth (H) as well as Sholl analyses (I) of primary hippocampal neurons that were cotransfected with the following combinations of plasmids, GFP+scr. RNAi, Ankrd24-GFP+scr. RNAi, Ankrd24-GFP+Cobl-like RNAi, and GFP+Cobl-like RNAi, respectively, at DIV4 and fixed 40 h thereafter (axon exclusion was additionally validated by anti-tau counterstainings). **(J)** Model depicting how ternary complexes composed of Ankrd24, syndapin I, and Cobl-like at dendritic branch induction sites and local force generation by F-actin formation (red arrow) may spatially steer and power the process of dendritic branch induction. Note that the cytosolic face of the plasma membrane has different membrane topologies (pointed out in scheme) in dendrites (concave and not curved [depending on orientation]) and at dendritic branch induction sites, which, at their base, require convex membrane surfaces for their protrusion. Ternary complex and molecular details of Ankrd24’s membrane association (insertion of an amphipathic helix N-terminal of Ankrd24’s ankyrin repeats into the cytosolic leaflet of the plasma membrane), syndapin I interaction (direct syndapin I SH3 domain/Ankrd24 KRAP motif association), and self-association (Ankrd24 coiled-coil region mediated) as well as of Ankrd24’s coupling to Cobl-like via syndapin I, which is membrane-bound by inserting wedges into the membrane and acts as dimers and therefore offers two SH3 domains for indirect Ankrd24/Cobl-like interconnection) are depicted in the box. Data, mean ± SEM shown as bar plot (H and I upper panel), line plot (I lower panel), or bar/dot plots overlaid with the individual data points in different colors representing different sources of primary cultures (E–G). GFP+scr. RNAi, *n* = 59; Ankrd24-GFP+scr. RNAi, *n* = 59; Ankrd24-GFP+Cobl-like RNAi, *n* = 58; GFP+Cobl-like RNAi, *n* = 53 neurons of 2–4 independent coverslips each per condition and assay and three independent neuronal preparations. GFP+scr. RNAi and Ankrd24-GFP+scr. RNAi correspond to Fig. 7, as conducted in joined multi-condition experiments. One-way ANOVA/Tukey’s (E and G), one-way ANOVA/Holm-Sidák’s (F), two-way ANOVA/Tukey's (H and I upper panel), and two-way ANOVA/Tukey’s (I lower panel), respectively. *p < 0.05; **p < 0.01; ***p < 0.001; ****p < 0.0001. For exact *p* values please see Supplementary Source data (Data S1) and/or figure; note that *p* values <0.0001 are not reported by Prism software). MIP, maximum intensity projection. Source data are available for this figure: SourceData F10. For numerical source data, see Data S1.

Our data have uncovered a functional cooperation of Ankrd24 and syndapin I that plays a critical role for dendritic arborization of developing neurons. Yet, it remained hard to imagine that local membrane topology modulations alone may be sufficient to drive the extensive cell morphology changes during neuronal development. We hypothesized that these functions of Ankrd24 may require some functional cooperation with effectors of the actin cytoskeleton that promote the formation of actin filaments and thereby provide the physical forces required for local cell shape changes. Indeed, we observed distinct sites of F-actin and Ankrd24 colocalization along dendrites (Fig. 10 A). Furthermore, 3D time-lapse imaging explicitly identifying sites of dendritic protrusion induction showed that Ankrd24-GFP and F-actin transiently occurred at these sites (Fig. 10, B and C).

As direct interactions of Ankrd24 with actin cytoskeletal components promoting F-actin formation were unknown, we addressed the formation of putative ternary Ankrd24 complexes with such components held together by syndapin I, which has the ability to self-associate using its N-terminal F-BAR domain leaving the SH3 domains of such syndapin I dimers free for binding to further interaction partners (Kessels and Qualmann, 2006). Three F-actin formation–promoting components seem to be of importance for dendritic branching, the actin nucleator Cobl (Ahuja et al., 2007), JMY (Zuchero et al., 2009; Kühne et al., 2025), and Cobl-like (Izadi et al., 2018). We were able to demonstrate that GFP-Cobl-like^1-457^ and syndapin I coprecipitated with GST-Ankrd24^1-285^ (Fig. 10 D). Importantly, this Ankrd24/Cobl-like association did not occur when syndapin I was absent (Fig. 10 D). Control experiments with merely immobilized GST or with GFP being offered instead of GFP-Cobl-like^1-457^ demonstrated that the identified indirect association of Cobl-like with Ankrd24 was specific and reflected ternary complexes composed of Ankrd24, syndapin, and Cobl-like (Fig. 10 D).

Cobl-like is able to promote actin filament formation and has a role in dendritogenesis (Izadi et al., 2018). We thus next addressed whether this complex formation would also reflect a functional dependence of the role of the N-Ank protein Ankrd24 in neuromorphogenesis on the cytoskeletal component Cobl-like. We observed that Cobl-like RNAi completely suppressed the Ankrd24-mediated effects. As frequently observed, the severity of the Ankrd24 gain-of-function phenotype was hereby diminished in the required double-transfection experiments. While similar to the single-transfection (Fig. 4) dendritic branching, dendritic terminal points, and dendritic branch depth level 3 were significantly increased, dendritic length and Sholl intersection increases did not reach statistical significances. Quantitative analyses revealed that Cobl-like RNAi–induced suppressions were occurring in the dendritic branch points, dendritic terminal points, and dendritic branch levels (depth 2–5) analyses and were statistically significant when compared with scr. RNAi+Ankrd24-GFP (Fig. 10, E–I). For dendritic branching points, dendritic terminal points, and dendritic branch depth levels 2 and 3, the suppression even was statistically significant when compared with the lower control values (scr. RNAi+GFP) (Fig. 10, E–I). Importantly, the dendritic parameters of Ankrd24-GFP– and Cobl-like RNAi–coexpressing neurons hereby were so low that they were indistinguishable from those determined for Cobl-like RNAi alone (Fig. 10, E–I).

These experiments demonstrated a strict requirement of the actin cytoskeleton effector Cobl-like for Ankrd24-mediated dendritic arborization and thereby identified Ankrd24 as a critical part of a larger, coordinated effort in inducing new dendritic branches off from already existing dendritic arbor during neuronal development (Fig. 10 J).

## Discussion

The N-Ank superfamily is suggested to represent a very special group among the plethora of ankyrin repeat proteins (Wolf et al., 2019). If experimentally followed up and underscored, the N-Ank hypothesis has the potential to fruitfully advance the understanding of ankyrin repeats in general and to furthermore provide insights into the mechanisms and functions of a group of proteins that may represent powerful membrane shapers and thereby sculpt cellular organelles or even entire cells. Our data clearly prove that Ankrd24 indeed is an N-Ank protein. We identify critical functions of Ankrd24 in the development of neurons, dissect the molecular mechanisms of Ankrd24 required for this function and, with syndapin I and Cobl-like, identify two proteins that play a crucial role in the coordination and execution of the identified Ankrd24’s functions in the development of proper neuronal morphology (Fig. 10 J).

We show that Ankrd24 uses its ankyrin repeats in combination with an N-terminal amphipathic helix to effectively associate with membranes. The existence and the critical importance of an amphipathic helix next to an ankyrin repeat array, as established for ankycorbin, UACA, Ankrd20A1 (Wolf et al., 2019) and Ankrd26 (Englisch et al., 2024), was suggested as common feature for N-Ank proteins (Wolf et al., 2019). In line with the N-Ank hypothesis, the phylogenetically closest relative of Ankrd24, UACA, also binds to and shapes membranes in an amphipathic helix–dependent and ankyrin repeat–involving manner (Wolf et al., 2019). The same is true for the second-closest member of the N-Ank superfamily, ankycorbin (Wolf et al., 2019). According to available data base information (https://proteinatlas.org), UACA and ankycorbin as well as a variety of other N-Ank proteins show rather ubiquitous protein expression profiles, including the brain. Ankycorbin shows a developmentally strongly regulated profile in the brain and in neurons (Wolf et al., 2019), which could point to a more critical role in specifically earlier steps of neuromorphogenesis. Our cloning of Ankrd24 from brains of 8-weeks-old mice suggests that, in contrast to ankycorbin, Ankrd24 is also abundant in older mice and may thus play a broader role. The extent by which N-Ank protein functions are competitive, similar, redundant, or even cooperative is unknown. It will be attractive to explore these scenarios further to understand the physiological functions of the emerging N-Ank protein superfamily.

The properties of Ankrd24 revealed a powerful mechanism to locally change membrane topologies. The insertion of an amphipathic helix in only one of the membrane leaflets would act like a wedge and provide considerable force to laterally expand selectively one membrane leaflet—a situation that can only be accommodated for if the membrane locally bends (McMahon and Gallop, 2005; Qualmann et al., 2011). In line, we observed that association of Ankrd24’s N-Ank domain with membranes led to stronger curved membrane, i.e., to membrane structures with smaller diameter, and that deletion of the N-terminal amphipathic helix completely abolished this Ankrd24 function. With about 50–500 nm, the curvatures observed in our ultrastructural analyses were roughly comparable with those observed for other N-Ank proteins (Wolf et al., 2019; Englisch et al., 2024) and were in a physiologically relevant range.

N-Ank modules are particularly suitable for membrane sculpting, as ankyrin repeats show high array stability and rigidity by lateral stacking. The curvature and rotational twist from one ankyrin repeat to the next one is defined and remains the same throughout the array (Breeden and Nasmyth, 1987; Lux et al., 1990; Li et al., 2006).

It furthermore seems likely that a certain, yet to be determined minimal extension of the array needs to be kept to provide enough membrane surface coverage for curvature sensing. The few N-Ank proteins characterized for their putative membrane-shaping abilities up to today all seem to comprise five to seven clearly recognizable ankyrin repeats (Wolf et al., 2019; Englisch et al., 2024). Ankrd24 has at least five clearly recognizable ankyrin repeats.

Mechanistically, local membrane curvature induced by a single, membrane-docked N-Ank module will also provide directly neighbored membrane areas with similar or at least with residual convex surface topologies. Because of their already convex nature, these surfaces will be preferred by further crescent-shaped N-Ank modules. Together with Ankrd24’s coiled-coil domain–mediated self-association (Krey et al., 2022; this study), this will lead to spatial self-organization resulting in Ankrd24-enriched membrane domains. Indeed, we observed high densities of Ankrd24 N-Ank modules at strongly bend surfaces, i.e., at small liposomes, in our quantitative EM studies.

The reported localization of inner hair cell Ankrd24 to stereocilia insertion points (Krey et al., 2022; Kazemi et al., 2025; Belyantseva et al., 2025) may in part also relate to membrane curvature preferences and/or inductions. The cytoplasmic side of the plasma membrane at the base of cellular protrusions, such as dendritic branches but also stereocilia, is marked by sharply bent convex topologies (Wolf et al., 2019). Apart from this, however, Ankrd24 has also been localized along the rootlets of stereocilia, i.e., along cytoskeletal structures pointing into the cytoplasm (Krey et al., 2022), suggesting that, besides membrane surfaces, also protein-binding partners can significantly modulate Ankrd24 localizations. It furthermore seems possible that also the mechanistically uncharacterized role of Ankrd24 in autophagy processes in U251MG glioblastoma cells (Hu et al., 2023) may relate to the membrane-binding and membrane-shaping abilities of Ankrd24 we identified, as also autophagosomes are marked by convex membrane surfaces at their cytoplasmic face.

In developing neurons, our examinations revealed that Ankrd24 localizes to dendrites and Ankrd24 loss-of-function led to highly statistically significant defects in dendritic arborization. Dendritic branch formation processes are key for neuronal network establishment during development but also during brain maturation during emerging adulthood (González Delgado et al., 2025) and during repair processes, such as those after ischemic stroke (Ji et al., 2021). The identified phenotype was specific and did not only require Ankrd24’s N-Ank module but explicitly also the N-terminal amphipathic helix, as demonstrated by rescue experiments with either wild-type Ankrd24 or mutants thereof. Thus, Ankrd24’s membrane-binding and membrane-bending abilities seem to be critical for its function in dendritic arbor formation. In line, overexpression of Ankrd24 had the opposite effect and resulted in more dendritic branch points, more terminal points, increased length of the dendritic arbor, and increased complexity of the dendritic arbor. Specifically, our quantitatively evaluated time-lapse experiments demonstrated that Ankrd24 promoted an increase in the formation of new dendritic protrusions, a key cellular process for the development of proper dendritic arbors of neurons. In line with this function, Ankrd24 was detected at the base of forming dendritic protrusions.

Strikingly, our results revealed that Ankrd24 does not act alone. Instead, it interacts with syndapins. A similar observation was made for ankycorbin, which also plays a role in dendritic arbor formation (Wolf et al., 2019; Izadi et al., 2023). The Ankrd24 interaction with syndapin I clearly is of cellular relevance, as it was demonstrated by coimmunoprecipitations and by reconstitution in intact cells. The interaction is direct and mediated by the SH3 domain of syndapins. It also is dependent on a highly conserved +KRxAPxPP aa motif in Ankrd24. The identified Ankrd24 interface shows considerable similarity with other syndapin SH3 domain interaction motifs, such as those in Cobl, ProSAP1/Shank2, Cobl-like, ankycorbin, EHBP1, and rhotekin2 (Schwintzer et al., 2011; Schneider et al., 2014; Izadi et al., 2021; Izadi et al., 2023; Ji et al., 2024; Tröger et al., 2025). Systematic yeast-2-hybrid analyses also suggested an Ankrd24/syndapin I interaction (Bandyopadhyay et al., 2010). As none of the other members of the N-Ank superfamily contains a recognizable KRAP motif for syndapin binding, this feature distinguishes ankycorbin and Ankrd24 from all other N-Ank proteins.

Syndapin I belongs to the BAR domain superfamily (Itoh et al., 2005; Qualmann et al., 2011; Carman and Dominguez, 2018; Simunovic et al., 2019) and plays an important role in dendritic branching (Schwintzer et al., 2011; Koch et al., 2020). We have demonstrated that membrane-bound syndapin I can recruit Ankrd24 in intact cells and made related observations in *in vitro* reconstitutions with purified components. The Ankrd24 recruitment by membrane-bound syndapin I was based on the identified Ankrd24 +KRxAPxPP (KRAP) motif–based interaction of the two proteins. The observed coaccumulation of Ankrd24 and syndapin I at sites of high convex membrane curvature suggests cooperative functions. In line, syndapin I had been reported to accumulate at nascent dendritic branch sites (Hou et al., 2015; Izadi et al., 2021). Importantly, our functional analyses of dendritic arbor development in primary neurons firmly demonstrated that Ankrd24 functions critically relied on both syndapin I binding, as revealed by the fact that Ankrd24^ΔKRAP^ failed to rescue any of the Ankrd24 loss-of-function phenotypes in dendritic arbor formation, as well as on syndapin I presence, as shown by a full suppression of Ankrd24 functions by syndapin I deficiency.

Dendritic branch induction sites often show spatially and temporally restricted buildup of filamentous actin suggesting that cytoskeletal forces are employed to enable or support dendritic branching (Hou et al., 2015; Izadi et al., 2018). Ankrd24 was observed to be present at dendritic sites that also were marked by relatively high F-actin abundance. Strikingly, we identified the formation of ternary complexes comprising Ankrd24, syndapin I, and Cobl-like, and our functional analyses clearly demonstrated that Ankrd24-mediated dendritic branching was strictly dependent on Cobl-like. It thus seemed that the critical dependence of Ankrd24-mediated dendritic branching on syndapin I reflected a use of syndapin I as the bridging component within the identified ternary complexes (Fig. 10 J). It is to our knowledge currently unknown whether ankycorbin or any other N-Ank superfamily protein forms similar complexes with cellular machinery promoting local F-actin formation, such as Cobl-like.

A ternary subcomplex composed of the membrane-binding and -shaping N-Ank protein Ankrd24, syndapin I, and Cobl-like appears to power dendritic branching of primary hippocampal neurons in a coordinated manner (Fig. 10 J). Based on the identified molecular properties of Ankrd24, it is plausible that Ankrd24 is part of a critical membrane interface for this cellular process leading to proper neuronal network formation in the brain by its power to shape membranes into convex topologies using its N-Ank module.

## Materials and methods

### Ankrd24 cloning and DNA constructs

Brains of 8-wk-old mice were dissected, snap-frozen in liquid nitrogen, and homogenized under liquid nitrogen by grinding using a mortar and a pestle. The tissue powder obtained was then resuspended in TRIzol reagent (Invitrogen). Two µg of DNase-treated (RNase-free DNase kit, QIAGEN) total RNA were reversely transcribed using RevertAid H Minus Reverse Transcriptase (Fermentas) and oligo(dT)-primers. The RNA isolation and reverse transcription PCRs thereby followed procedures described previously (Haag et al., 2018). Full-length *M. musculus* Ankrd24 (NCBI Reference Sequence XM_006514096.5; XP_006514159.1 for the corresponding protein [aa 1–1041; isoform x5]) was cloned from the cDNA prepared and inserted into pEGFP-N3. Ankrd24^1-285^ was also cloned from the prepared cDNA and inserted into pGEX-6P1 and pEGFP-N3.

GST-Ankrd24^10-285^ was cloned via an internal AgeI site into pGEX-6P-1. Ankrd24^1-222^ and Ankrd24^10-222^ were amplified via PCR and inserted into pGEX-6P-1. For primers used see Table 1.

**Table 1.** Sequences of primers used

Purpose, aa representing annealing position, internal reference number and sequence of primer (*M. musculus* Ankrd24; GI: XP_006514159.1)	Restr. site
Forward primer: aa1-5 (BQ2893) 5′-ATAGAATTCGCCACCATGAAGACCCTCCGG-3′	EcoRI
Forward primer: aa1-13 (BQ2940) 5′-ATAGAATTCGCCACCATGAAGACCCTCCGGGCACGATTCAAGAAAACAGAGGGC-3′	EcoRI
Forward primer: aa10-17 (BQ3208) 5′-ATAGAATTCGCCACCATGAAAACAGAGGGCCAAGACTGG-3′	EcoRI
Reverse primer: aa285-281 (BQ2894) 5′-TATGTCGACTGTGCTGGCTGGAGGCTG-3′	SalI
Reverse primer: aa222-218 (BQ2895) 5′-TATGTCGACAGTGGCATCCTGGCCCAG-3′	SalI
Reverse primer: aa1041-1037 (BQ2907) 5′-TATGTCGACGCGACCCTGAGCCTG-3′	SalI
Forward primer: 478-497bp/139-145aa RNAi#1 (BQ3130) 5′-GATCCGCTCAGATGTGTCACACAGATTGATATCCGTCTGTGTGACACATCTGAGCTTTTTTA-3′	BamHI
Reverse primer: 478-497bp/139-145aa RNAi#1 (BQ3131) 5′-AGCTTAAAAAAGCTCAGATGTGTCACACAGACGGATATCAATCTGTGTGACACATCTGAGCG-3′	HindIII
Forward primer: 388-408 bp/130-136 aa RNAi#2 (BQ3136) 5′-GATCCGGTTGTTTGTCCTGCTCAAAGTTGATATCCGCTTTGAGCAGGACAAACAACCTTTTTTA-3′	BamHI
Reverse primer: 388-408 bp/130-136 aa RNAi#2 (BQ3137) 5′-AGCTTAAAAAAGGTTGTTTGTCCTGCTCAAAGCGGATATCAACTTTGAGCAGGACAAACAACCG-3′	HindIII
Forward primer: (RNAi#1 insensitive mutation) (BQ3312) 5′-CTCATCATAGCAGCACAAATGTGCCATACGGATCTGTGCCGCCTC-3′	​
Reverse primer: (RNAi#1 insensitive mutation) (BQ3313) 5′-GAGGCGGCACAGATCCGTATGGCACATTTGTGCTGCTATGATGAG-3′	​
Forward primer: (RNAi#2 insensitive mutation) (BQ3314) 5’-GCAGCAGCCGGTGGGTGCTTATCATGTTCGAAACTGCTCTGTTCC-3’	​
Reverse primer: (RNAi#2 insensitive mutation) (BQ3315) 5′-GGAACAGAGCAGTTTCGAACATGATAAGCACCCACCGGCTGCTGC-3′	​
Forward primer: aa252-272+287-292 (BQ3362) 5′-GAGGAGGACTCCGGAGAGGCCTCATCCCAGAACTCAGTGTCAAGCCATGAGAAACAAGGAGCCGTTCCTGACGATCGGGAT-3′	Kpn2I and PvuI internal sites
Reverse primer: aa252-272+287-292 (BQ3363) 5′-ATCCCGATCGTCAGGAACGGCTCCTTGTTTCTCATGGCTTGACACTGAGTTCTGGGATGAGGCCTCTCCGGAGTCCTCCTC-3′	Kpn2I and PvuI internal sites
Forward primer: 277-289 (BQ2999) 5′-ATAGAATTCGCCACCATGGGAAGGCACCCCAGCCTCCAGCCAGCACACCAGTTCCTGACG-3′	EcoRI
Reverse primer: Ankrd24 aa285-282 + linker (VDASVSHDAAARIDCAGW) (linker addition between Ankrd24^1-285^ and GFP in GST-Ankrd24^1-285^-GFP) (BQ4057) 5′-TTGTCGACGCGTCCGTCAGTCACGATGCGGCCGCTCGAATCGACTGTGCTGGCTGG-3′	​

Ankrd24^Δ273-286^-GFP (Ankrd24^ΔKRAP^-GFP) was cloned by PCR and using the Kpn2I and PvuI restriction sites and inserted into pEGFP-N3. Ankrd24^283-1041^-GFP was cloned by PCR and inserted into pEGFP-N3.

GST-Ankrd24^1-285^-GFP was generated by PCR and insertion (using EcoRI/SalI) into a pGEX-6P-1 derivative into which GFP had been inserted (Izadi et al., 2023). The resulting construct encodes for GST-Ankrd24^1-285^-GFP with the aa sequence VDASVSHDAAARIDCAGW serving as linker between Ankrd24^1-285^ and GFP. The fusion protein also contained a PreScision proteas cleavage site for proteolytic removal of GST from the N terminus of Ankrd24^1-285^. For primers used see Table 1.

Ankrd24 RNAi#1 (Ankrd24 RNAi) (target site encodes aa 160–166 of either *Rattus norvegicus* [XP_063119259.1; isoform x1] or *M. musculus* Ankrd24 [XP_006514159.1; isoform x5]) was generated by subjecting the forward primer 5′- GAT​CCG​CTC​AGA​TGT​GTC​ACA​CAG​ATT​GAT​ATC​CGT​CTG​TGT​GAC​ACA​TCT​GAG​CTT​TTT​TA-3′ and the reverse primer 5′- AGC​TTA​AAA​AAG​CTC​AGA​TGT​GTC​ACA​CAG​ACG​GAT​ATC​AAT​CTG​TGT​GAC​ACA​TCT​GAG​CG-3′ to primer annealing, phosphorylation, and subcloning into pRNAT-H1.1/Hygro-GFP (GenScript) using its BamHI and HindIII sites.

Ankrd24 RNAi#2 (target site encodes aa 130–136 of either *M. musculus* Ankrd24 or *R. norvegicus* Ankrd24 x1 isoform [except for 1 b]) was generated accordingly using the forward primer 5′- GAT​CCG​GTT​GTT​TGT​CCT​GCT​CAA​AGT​TGA​TAT​CCG​CTT​TGA​GCA​GGA​CAA​ACA​ACC​TTT​TTT​A-3′ and the reverse primer 5′- AGC​TTA​AAA​AAG​GTT​GTT​TGT​CCT​GCT​CAA​AGC​GGA​TAT​CAA​CTT​TGA​GCA​GGA​CAA​ACA​ACC​G-3′.

The pRNAT-H1.1/Hygro-GFP plasmid expressing a scr. RNAi sequence was as described (Wolf et al., 2019).

RNAi tools for validation of knockdown (Ankrd24 RNAi, Ankrd24 RNAi#2, and scr. RNAi for control) were complemented with DNA sequences expressing both Ankrd24-GFP (for knock-down validation) and mCherryF as reporter.

RNAi-insensitive Ankrd24 (Ankrd24*) was obtained by introducing silent mutations into the RNAi target site using 5′- CTC​ATC​ATA​GC**A**GC**A**CA**A**ATG​TG**C**CA**T**AC**G**GAT​CTG​TGC​CGC​CTC-3′ as forward primer and 5′- GAG​GCG​GCA​CAG​ATC**C**GT**A**TG**G**CAC​AT**T**TG**T**GC**T**GCT​ATG​ATG​AG-3′ as reverse primer (nucleotides with point mutation resulting in silent mutations are shown by bold letters) and insertion into pEGFP. Ankrd24 insensitive for Ankrd24 RNAi#2 was generated in a similar manner. Subsequently, the sequences encoding for the GFP-tagged, RNAi-resistant Ankrd24 mutants (Ankrd24*) were subcloned into Ankrd24 RNAi– and scr. RNAi–encoding plasmids, respectively, replacing the GFP sequence of pRNAT.

For additional rescue attempts, several corresponding Ankrd24* deletion mutants were constructed. These mutants included Ankrd24*^ΔKRAP^-GFP (Δaa 273–286; cloned by the use of internal XbaI and AfeI sites), Ankrd24*^cc^-GFP (Ankrd24*^283-1041^-GFP; by subcloning from Ankrd24^2^^77^^-1041^-GFP using an internal restriction site), Ankrd24*^Δhelix^-GFP (Δaa 1–9; use of the internal AgeI site as well as of internal XcmI and NarI sites, respectively), and Ankrd24*^1-285^-GFP (subcloned from pEGFP derivative).

Rat GST-SdpI^1-441^, GST-syndapin I^P434L^, GST-SdpII-l^1-488^, GST-SdpII-s^1-447^ (Qualmann and Kelly, 2000), and GST-SdpIII^1-424^ (Izadi et al., 2021) have been described previously. For easier GST-tag removal, syndapin I^P434L^ was additionally subcloned into pGEX-6P-1. Syndapin I in pGEX-6P-1 was subcloned into pGEX-6P-1, too (Izadi et al., 2021).

Flag-syndapin I (Qualmann and Kelly, 2000), mCherry-tagged and mitochondrially targeted syndapin I were as described (Mito-mCherry-SdpI; Schneider et al., 2014). GST-mCherry-syndapin I was built for visual analyses of in vitro reconstitutions by inserting mCherry (amplified by PCR) between GST and syndapin I in GST-syndapin I using the BamHI site of pGEX-6P-1 (Izadi et al., 2023).

Syndapin I RNAi (driven by pRNAT-H1.1 and reported by GFP) was validated for successful knockdown (Dharmalingam et al., 2009) and for specificity via rescue experiments in phenotypical analyses using silently mutated, RNAi-insensitive syndapin I (Schneider et al., 2014).

The vector expressing plasma membrane–targeted, mCherryF was originally provided by M. Korte (TU Braunschweig, Germany) and used as a reporter by coexpression before (Schneider et al., 2014). mCherryF specifically outlines the plasma membrane (Hou et al., 2015; Wolf et al., 2019).

GFP-Cobl-like^1–457^ was generated by PCR (Izadi et al., 2021). The Cobl-like RNAi plasmid (RNAi#1) coexpressing mCherryF as reporter was described and characterized by Izadi et al. (2021). It was generated by replacing the GFP reporter in pRNAT H1.1 by mCherryF. LifeAct-RFP was a gift from K. Murk (Charité Universitätsmedizin Berlin, Berlin, Germany).

Correct cloning by PCR was verified by sequencing in all cases. For primers used see Table 1.

### Antibodies

Polyclonal guinea pig and rabbit anti–syndapin I antibodies were raised against GST-tagged rat syndapin I^1-382^ and affinity purified with immobilized maltose-binding protein-tagged syndapin I^1-382^ (Qualmann et al., 1999). Rabbit anti-GST antibodies were affinity purified from anti–GST-syndapin I sera using immobilized GST (Qualmann et al., 1999). Guinea pig anti-GST antibodies were obtained analogously from sera of guinea pigs subjected to GST-fusion protein immunizations. Affinity purifications of serum collected after 100 days were done with immobilized, purified, recombinant TrxHis-tagged Cobl-like^537-1015^ (Izadi et al., 2018). Polyclonal rabbit anti-Lamin B1 (ab16048) and rabbit anti-mCherry (ab167453; RRID AB_2571870) antibodies were from Abcam. Chicken anti-GFP antibodies were from Aves (GFP1010). Monoclonal mouse anti-GFP antibodies (JL8; RRID AB_10013427) were from Clontech. Rabbit (F7425) and mouse anti-Flag antibodies (M2; F3165) as well as monoclonal mouse anti-MAP2 antibodies (HM-2; M4403) were from Sigma-Aldrich. Polyclonal guinea pig anti-MAP2 antibodies were from Synaptic Systems (188004; RRID AB_213818). Anti-tau antibodies used were mouse monoclonal anti-tau1 (Sigma-Aldrich; discontinued) and mouse monoclonal anti-tau5 (Abcam, 80579). Goat anti-Ankrd24 antibodies (sc-241811; RRID:AB_10851440) were from Santa Cruz. Rabbit control IgG was from Santa Cruz.

Secondary antibodies included Alexa Fluor 568–labeled goat anti-guinea pig antibodies (RRID AB_141954), goat anti-mouse AlexaFluor 680 (A21058), goat anti-rabbit AlexaFluor 680 (A32734; RRID AB_1965956), goat anti-rabbit DyLight 800 (SA5-35571; RRID AB_2556775), and goat anti-mouse DyLight 800 (SA5-35521; RRID AB_2556774) from Thermo Fisher Scientific. Furthermore, donkey anti-guinea pig IRDey680 (LI-COR 926–68077; RRID AB_10956079), donkey anti-guinea pig IRDey800 (LI-COR 926–32411; RRID AB_1850024), donkey anti-goat IRDye800CW (LI-COR 926–32214; RRID AB_621846), and donkey anti-chicken-IRDye800CW (926–32218) from LI-COR Bioscience were used. Donkey anti-goat AlexaFluor 488 (A-11055) and donkey anti-rabbit Alexa Fluor 568 (A10042) were from Thermo Fisher Scientific, whereas donkey anti-guinea pig AlexaFluor 647 antibodies (706-605-148; RRID AB_2340476) were from Jackson ImmunoResearch. EM grade 10 nm gold anti-mouse conjugates (EM.GAM10/2) were purchased from BBI Solutions.

### Purification of recombinant proteins and tag cleavage

GST fusion proteins were expressed in and purified from *Escherichia coli* (BL21-CodonPlus (DE3)-RIPL) using standard methods. Expression was at 37°C for 4 h. Lysis of *E. coli* was done with 1% (vol/vol) Triton X-100 in PBS containing EDTA-free Complete protease inhibitor cocktail (Roche), 10 mM MgCl_2_, and DNase I. In some experiments, 1 mM DTT was added. High-affinity glutathione resin (Antibodies Online) was used as matrix, and elutions of GST fusion proteins were carried out with elution buffer (20 mM reduced glutathione, 50 mM Tris/HCl, pH 8.0, and 120 mM NaCl).

Cleavages of pGEX-6P-1–encoded GST fusion proteins were essentially performed as described (Wolf et al., 2019). In brief, PreScission Protease (GE) cleavage was performed overnight (4°C) during dialysis against HN-buffer (20 mM HEPES, pH 7.4, 150 mM NaCl, and 2.5 mM DTT). GST and remaining GST-fusion proteins were removed with glutathione resin (Antibodies Online). Protein concentrations were determined by Bradford assay. Successful cleavage and protein integrity were verified by SDS-PAGE and Coomassie staining.

### Culturing of cell lines, transfection, and imaging

HEK293 cells (RRID: CVCL_0045), HeLa cells (RRID: CVCL_0030), COS-7 cells (RRID: CVCL_0224), and L929 cells (RRID: CVCL_L929) were cultured under standard conditions (37°C with 90% humidity and 5% CO_2_) and transfected using TurboFect (Thermo Fisher Scientific) according to instructions of the manufacturer. The cell lines are regularly tested for mycoplasma and were mycoplasma-negative.

HeLa cells were seeded on poly-D-lysine–coated coverslips (diameter, 12 mm) in 24-well plates and transfected with TurboFect following the instructions of the manufacturer. After 12 h, HeLa cells were fixed with 4% (wt/vol) paraformaldehyde in PBS for 3 min. The cells were subsequently incubated in 25 mM glycine in PBS at RT for 20 min, washed with PBS and H_2_O, and mounted on coverslips with Mowiol. COS-7 cells were fixed 24 h after transfection.

Images were recorded as z-stacks using a Zeiss Axio-Observer.Z1 microscope (Zeiss) equipped with an ApoTome, Plan-Apochromat 100×/1.4, 63×/1.4, 40×/1.3, and 20×/0.5 objectives, and an AxioCam MRm CCD camera (Zeiss). Digital images were recorded by ZEN2012. Image processing was done by Adobe Photoshop. Line scans calculations were done by ImageJ.

### Coprecipitation assays

HEK293 and L929 cells, respectively, were transfected with plasmids encoding GFP-tagged proteins and lyzed using 10 mM HEPES, pH 7.4, 1 mM EGTA, 0.1 mM MgCl_2_, 1% [vol/vol] Triton X-100 (lysis buffer) containing 150 mM NaCl, and Complete protease inhibitor cocktail. Coprecipitation analyses with immobilized, purified, recombinant GST fusion proteins were essentially carried out as described (Kessels and Qualmann, 2002). In detail, extracts from HEK293 and L929 cells, respectively, expressing different GFP-fusion proteins were incubated 2 h at 4°C with purified GST-fusion proteins immobilized on glutathione resin. GST-fusion protein binding, lysate incubation, and washing were performed in lysis buffer with 150 mM NaCl. Bound protein complexes were eluted by incubating the samples in SDS sample buffer and urea at 95°C for 5 min. The eluted proteins were then analyzed by immunoblottings using anti-GFP and anti-GST antibodies and a LI-COR Odyssey System for fluorescence detection (LI-COR Biosciences).

### In vitro reconstitution of direct protein interactions

Direct protein interactions were demonstrated by coprecipitation assay with purified recombinant proteins. Used were immobilized GST and GST-Ankrd24^1-285^ in combination with recombinant untagged syndapin I according to procedures resembling those described before (Izadi et al., 2021). In detail, recombinant, untagged syndapin I was obtained by GST-syndapin I expression, purification, and subsequent GST tag removal via PreScission Protease cleavage as described above. Syndapin I was incubated with GST and GST-Ankrd^1-285^ immobilized on a glutathione resin in lysis buffer supplemented with 150 mM NaCl and EDTA-free Complete protease inhibitor cocktail for 2 h at 4°C. After washing with lysis buffer containing 150 mM NaCl, bound protein complexes were eluted with elution buffer and analyzed by anti-SdpI and anti-GST immunoblotting.

### Heterologous coimmunoprecipitations

Heterologous coimmunoprecipitations were essentially conducted as described (Hou et al., 2018; Izadi et al., 2021). In brief, L929 cells cotransfected with Flag-tagged syndapin I and GFP-tagged Ankrd24 proteins were lyzed by addition of lysis buffer containing 50 mM NaCl and Complete protease inhibitor cocktail, and the lysates were incubated at 4°C for 2 h with 5 µg mouse monoclonal anti-Flag antibodies (M2) and with 5 µg unrelated murine IgG (Santa Cruz), respectively. Subsequently, 15 μl of a suspension (1:2 vol:vol in lysis buffer) of protein A/G-agarose (Santa Cruz) was added to each sample, incubated for another 2 h, and then isolated by centrifugation. Protein/antibody complexes bound to the agarose were then washed three times with lysis buffer containing 150 mM NaCl, eluted in 2× SDS sample buffer (5 min, 95°C), and then analyzed by immunoblotting using guinea pig anti–syndapin I antibodies for detection of immunoprecipitated proteins and chicken anti-GFP antibodies for detection of coimmunoprecipitated GFP-fusion proteins.

### Animals

Mice (BL6) and rats (Wistar) of either sex were used to obtain biological material. The animals were bred by the animal facility of the Jena University Hospital in strict compliance with the EU guidelines for animal experiments. The procedures were approved by the Thüringer Landesamt für Verbraucherschutz (Bad Langensalza; Germany; allowance for primary hippocampal culture preparations from rat embryos, UKJ-24-005).

### Coprecipitation of endogenous syndapin I from rat brain lysates

For coprecipitation of endogenous syndapin I, brain lysates were prepared from about 15-wk-old rats (Wistar) sacrificed by cervical dislocation as described (Schwintzer et al., 2011; Izadi et al., 2021), i.e., extracts were prepared in lysis buffer containing EDTA-free Complete protease inhibitor cocktail and 150 mM NaCl using a potter (12 strokes). After 30,000 × *g* centrifugation for 20 min, the supernatants were used for coprecipitation analyses of endogenous proteins. GST served as a specificity control. The proteins were immobilized on glutathione sepharose and incubated with the prepared rat brain lysates for 2 h at 4°C. After washing, bound proteins were eluted with elution buffer, subjected to SDS-PAGE, and analyzed by anti–syndapin I and anti-GST immunoblotting.

### Preparation of membrane-enriched subcellular fractions of HEK293 cells

HEK293 cells were transfected with plasmids encoding for GFP-tagged Ankrd24 proteins and GFP in combination with mCherryF (plasma membrane marker). 24 h after transfection, the cells were washed with PBS, harvested, collected by centrifugation (5 min, 100 × *g*, 4°C), and resuspended in homogenization buffer (0.32 M sucrose, 5 mM HEPES, pH 7.4, and 1 mM EDTA) containing Complete protease inhibitor cocktail. The suspended cells were then homogenized by multiple passages through a 0.12-mm syringe.

The resulting homogenates were fractionated as described (Wolf et al., 2019), i.e., the homogenates were first subjected to centrifugation at 4°C for 10 min at 1,000 × *g* yielding fractions S1 and P1. S1 was then centrifuged further (20 min, 11,700 × *g*). After removal of the supernatant S2, the resulting membrane-containing pellet P2 was resuspended in homogenization buffer und again centrifuged at 11,700 × *g* for 20 min. This resulted in fractions S2′ and P2′ with P2′ representing a washed membrane-containing fraction P2. All pellet fractions were resuspended in volumes equal to those of the corresponding supernatants. SDS-PAGE was performed with equal amounts of supernatant and pellet fractions, and the samples were analyzed by immunoblotting.

### Cross-link studies

Cross-link studies with lysates of HEK293 cells overexpressing different GFP fusion proteins were essentially done as established (Kessels and Qualmann, 2006; Wolf et al., 2019), i.e., the cell lysates were incubated with increasing amounts of the heterobifunctional cross-linker 1-ethyl-3-[dimethylaminopropyl]carbodiimide (Pierce) in 20 mM HEPES, pH 7.4, 0.2 mM MgCl_2_, and 2 mM EGTA for 20 min at RT. Subsequently, the cross-linking reaction was stopped by adding 4× SDS sample buffer and incubating the samples at 95°C for 5 min. The samples were analyzed by immunoblotting.

### Ternary complex examinations

Coprecipitations of three protein partners were essentially performed according to Izadi et al. (2021). In brief, the possibility of complex formations of Ankrd24, syndapin I, and Cobl-like in ternary complexes was experimentally addressed by using purified, immobilized GST-Ankrd24^1-285^ (control, GST), purified untagged syndapin I (putative bridging component; for preparation procedures, see above), and GFP-Cobl-like^1–457^ (syndapin-binding partner; offered in form of lysates of transfected HEK293 cells, see above; control, GFP). Ternary complex examinations were essentially performed as the coprecipitation studies described above (using a lysis buffer containing 150 mM NaCl) except that the lysates of the cells were not only incubated with immobilized GST-Ankrd24^1-285^ but also with tag-free syndapin I (0.1 mg/sample) for 2 h at 4°C.

After washing with lysis buffer containing 150 mM NaCl, bound proteins were eluted with elution buffer, separated by SDS-PAGE, and analyzed by anti–syndapin I, anti-GST, and anti-GFP immunoblotting.

### Liposome preparation

Liposomes were prepared from Folch fraction I lipids (Sigma-Aldrich). The lipids were dried and subsequently rehydrated by a steady stream of water-saturated nitrogen gas for 30 min. The lipids were then incubated in 30 ml 0.3 M sucrose in ddH_2_O at 42°C for 12 h. Liposomes were collected by centrifugation at 20,000 × *g* at 28°C for 1 h, and the pellet was resuspended in 500 μl 0.3 M sucrose in ddH_2_O to achieve a final lipid concentration of 8 mg/ml.

The size distribution of liposomes differs from preparation to preparation—with the mean diameter usually 350–550 nm. Therefore, control conditions were always included in all assays addressing liposome sizes.

### Freeze-fracturing of liposomes and quantitative TEM analyses of membrane curvature

Protein/liposome interactions were reconstituted with 100 μl of liposome suspension (2 µg/μl) in HN-buffer (20 mM HEPES, pH 7.4, 150 mM NaCl, and 2.5 mM DTT) and 1 µM of Ankrd24^1-285^ and Ankrd24^10-285^, respectively (15 min, at 800 rpm and 37°C).

In combinatory experiments, 0.05 µM Ankrd24^1-285^ were incubated with liposomes and with either 1 µM of syndapin I or syndapin I^P434L^ under the same conditions. In accordance with established procedures (Beetz et al., 2013), the samples were then treated with 0.15 µg/μl proteinase K at 45°C for 25 min to avoid liposomal aggregates and to allow for most accurate measurements.

Sample preparation and freeze-fracturing of liposomes were done as established (Wolf et al., 2019; Hofbrucker-MacKenzie et al., 2023; Izadi et al., 2023), i.e., 2 μl aliquots of concentrated liposome suspension obtained by centrifugation (200,000 × *g*, 28°C, 15 min) and suspension in 15 μl of the supernatant were placed between two copper profiles, and the sandwich was immediately plunge-frozen in liquid ethane cooled by liquid N_2_ (−180°C). The frozen samples were then put into a detachable cold table cooled by liquid N_2_. After transfer into the recipient of a BAF400T freeze-fracture unit (BAL-TEC), the samples were then freeze-fractured at 1 × 10^−6^ mbar and −140°C.

Platinum shadowing of liposomes was done as described (Hofbrucker-MacKenzie et al., 2023; Izadi et al., 2023). In brief, fractured liposome membranes were shadowed with 2-nm platinum/carbon at an angle of 35° in the BAF400T freeze-fracture unit. The replica were then stabilized by a 15–20-nm-thick carbon coat, extracted from the machine, thawed, incubated with 3% (vol/vol) sodium hypochlorite in ddH_2_O for 20 min, and then washed with ddH_2_O.

TEM analyses of replica from freeze-fractured liposomes were done as established (Seemann et al., 2017; Wolf et al., 2019; Izadi et al., 2023; Englisch et al., 2024). In brief, replicas of freeze-fractured liposomes were collected on uncoated copper grids (300 mesh) and viewed by a Zeiss EM900 electron microscope (Zeiss) run at 80 kV. Images were recorded by systematic grid explorations using either a 1 k FastScan CCD camera and EM-Menu 4 software or a wide-angle Dual Speed 2K CCD camera (Tröndle) and processed with Adobe Photoshop. Diameters of freeze-fractured liposomes were measured using ImageJ (ImageJ/FIJI 1.46r) and Excel.

### Immunogold labeling of freeze-fracture replica of liposomes and their quantitative analyses

Liposomes were incubated either with buffer or with purified recombinant GFP and Ankrd24^1-285^-GFP (1 µM), respectively, in HN buffer (20 mM HEPES, pH 7.4, 150 mM NaCl, and 2.5 mM DTT) in a total volume of 1 ml at 800 rpm for 15 min at 37°C and then collected by ultracentrifugation as described above. The supernatant was removed from the pellet, and the pellet was carefully resuspended in 10 μl of the supernatant. For subsequent immunolabeling, the resulting concentrated suspension was directly subjected to freeze-fracturing. While the freeze-fracturing was done as described above, shadowing of the fractured liposome membranes was done first with carbon (coat at 90° for physical support) and only then with platinum at 35° (2 nm-thick coat) (Hofbrucker-MacKenzie et al., 2023). Subsequently, the samples were removed from the freeze-fracture machine, thawed, floated onto 2.5% (wt/vol) SDS, and incubated at very gentle shaking at RT overnight.

After three washes with PBS and blocking putative unspecific antibody interactions with 1% (wt/vol) BSA, 0.5% (wt/vol) fish gelatine, and 0.005% (vol/vol) Tween 20 in PBS, pH 7.2 (LBB), samples were incubated with mouse anti-GFP primary antibodies (JL-8; 1:50 in LBB) overnight at 4°C. The replica were then washed three times with LBB, labeled with goat anti-mouse 10 nm gold secondary antibodies (EM.GAM10/2; BBI solutions; 1:100 in LBB), washed three times for 10 min in PBS, fixed with 0.25% (vol/vol) glutaraldehyde in PBS for 10 min, washed in ddH_2_O twice, mounted onto uncoated copper grids, and dried.

For quantitative analyses of liposome-labeling densities, the membrane areas of individual liposomes were determined (in 2D) using ImageJ, and the gold labels were counted. Dissections of labeling densities according to liposomes size were done by using the mean diameter of control liposomes rounded up to the nearest hundred as cutoff for the distinction of small versus large liposomes, i.e., liposomes with >500 nm diameters were considered as large and ≤500 nm as small. Zero profiles were included to fully cover the downward spread of data.

### Liposome (co)sedimentation

Untagged wild-type Ankrd24^1-285^, Ankrd24^1-222,^ Ankrd24^10-285 ^and Ankrd24^10-222^, and full-length syndapin I were either individually or in combination diluted into 25 μl HN-buffer (final concentration 3 µM protein each) and prespun for 5 min at 200,000 × *g* at 28°C. The supernatant was transferred to a fresh tube and incubated with 50 µg of Folch fraction I liposomes (2 mg/ml) for 30 min at RT. Samples without liposomes controlled for putative unspecific protein precipitation. The samples were then centrifuged (200,000 × *g*, 20 min, 28°C), and the supernatants were collected. The pellets were resuspended in volumes corresponding to the removed supernatants. The pellet and supernatant samples were then analyzed by SDS-PAGE. Coomassie-stained gels were quantified with a LI-COR Odyssey imager and software (LI-COR Biosciences).

Binding to liposomes was calculated in percent after subtracting putative non-liposome–associated precipitation using the equation

(P/(S+P ))*100 in liposome samples −(P/(S+P ))*100 control no−liposome samples

### Time-lapse imaging of fluorescent LUVs and proteins

Fluorescent LUVs and GUVs for time-lapse imaging were prepared by a gentle swelling procedure as described (Wolf et al., 2019). In brief, solutions of Folch fraction I lipids were supplemented with Cy5-PE (Avanti Polar Lipids; 5% [vol/vol] final), and the solvents were removed under a N_2_ current. The lipids were then rehydrated, and GUVs and LUVs were formed through gentle swelling in 0.3 M sucrose overnight at 42°C. 5 µg of the liposomes were then incubated with 2 µM of (each) purified fluorescent (fusion) protein in 400 μl cytosol buffer (25 mM HEPES, pH 7.2, 25 mM KCl, 2.5 mM Mg-acetate, and 100 mM K-glutamate), mixed, and transferred into an open coverslip holder placed in a temperature-controlled incubator built around a spinning disc microscope based on a motorized Axio Observer (Zeiss) equipped with a spinning disc unit CSU-X1A 5000, with a 488 nm/100 mW OPSL laser, with 545 nm/40 mW diode lasers, as well as with a QuantEM 512SC EMCCD camera (Zeiss).

The mixtures were imaged at 37°C and low laser powers (12% laser power; exposure times, 40 ms per channel; 63×/1.20W Korr M27 objective; Zeiss), with a frame rate of 1 multiple color Z-stack per 10 s for 10 min.

Image recording and processing were performed using ZEN2012 (PRID: SCR_013672), ImageJ (RRID: SCR_003070), and Adobe Photoshop (RRID: SCR_014199) software.

### Culturing, transfection, and immunostaining of primary rat neurons

Rat hippocampal neurons (rat strain, Wistar; sex, mixed) were prepared and cultured until day in vitro (DIV) 4 as described (Wolf et al., 2019; Izadi et al., 2021). The procedure was approved by the Thüringer Landesamt für Verbraucherschutz (Bad Langensalza; Germany; allowance for primary hippocampal culture preparations from rat embryos, UKJ-24-005). In brief, brains and hippocampi were isolated from a full litter of E18 rats (∼14 embryos of mixed sex). Neurons from the entire cohort of embryos were prepared from their hippocampi, pooled, and seeded into 24-well plates at densities of 60,000/well. The neurons were cultured in Neurobasal medium containing 2 mM L-glutamine, 1× B-27, and a mixture of 100 U/ml penicillin and 100 µg/ml streptomycin (Invitrogen) and maintained at 37°C with 90% humidity and 5% CO_2_. Transfections were done in antibiotic-free medium using 1 μl Lipofectamine 2000 (Thermo Fisher Scientific) and 1 µg DNA (each plasmid) per well. After 4 h, the transfection medium was replaced by a conditioned medium. After 40 h, neurons were fixed by 5–7-min incubation in 4% (wt/vol) paraformaldehyde.

After quenching with 25 mM glycine in PBS at RT for 20 min as well as permeabilization and blockage by blocking solution (10% [vol/vol] horse serum, 5% [wt/vol] BSA, and 0.2% [vol/vol] Triton X-100 in PBS pH 7.4) for 1 h at RT, the samples were immunostained with primary antibodies, washed three times with blocking solution, and then incubated with secondary antibodies and DAPI. After washing, the coverslips were mounted onto glass slides using Mowiol.

### Imaging and quantitative assessments of neuronal morphology

Using a Zeiss Axio Observer Z1 microscope/Apotome (objectives, Plan-Apochromat 100×/1.4, 63×/1.4 and 40×/1.3) and an AxioCam MRm CCD camera, transfected neurons were imaged by systematic sweeps across the coverslips. Digital images were recorded by ZEN2012 and processed by Adobe Photoshop.

Morphometric measurements were done for dendrites. The axons were excluded by a combination of their only low, merely proximal (axon initial segment) presence of the dendritic marker MAP2 and by morphological criteria according to Craig and Banker (1994) (i.e., [1] longest neurite extending far beyond the field of view focusing on the dendritic arbor, [2] caliber of the axon adjacent to the cell body being smaller than that of dendrites, [3] diameter of the dendrite decreasing at each branch point, whereas the axonal diameter remains rather constant, and [4] angle between primary neurite and branch being smaller in dendrites than in axons) and/or by counterstainings for the axon marker tau. About half of the assays had additional anti-tau immunostainings. These assays were used to also quantitatively compare axon exclusion by all six above criteria versus dendrite identifications based on the dendritic marker MAP2 and the four morphological criteria.

Dendritic neuronal morphologies were reconstructed in 3D using Apotome image stacks processed by Imaris 8.4.0 software. After axon exclusion, the 3D reconstructions of the dendritic arbor were based on anti-MAP2 immunosignals as an endogenous staining independent from the respective transfection and the different fluorescent reporters used. According to procedures described before (Ji et al., 2024), the following settings were used: thinnest diameter, 2 µm; gap length, 2 μm; minimum segment length, 10 μm. The number of dendritic branching points, terminal points, total dendritic tree length, and Sholl intersections was analyzed as described (Ji et al., 2021; Ji et al., 2024). Additionally, branch depth levels (with level 1 representing primary dendrites) were determined using Imaris 8.4.0.

### 3D time-lapse analyses of developing primary hippocampal neurons

For time-lapse imaging, the medium of primary hippocampal neurons was replaced by Neurobasal A medium (Gibco) supplemented with 0.5 mM L-alanyl-L-glutamine, 0.025 mM L-glutamate, 1×B-27 supplement (Gibco), and penicillin/streptomycin (1:100; Gibco) 1 h after cell suspension seeding. At DIV6, the neurons were transfected with Lipofectamine 2000. For analyses of initiations of dendritic protrusions upon Ankrd24-GFP expression versus GFP controls, 3D time-lapse imaging was conducted 24–28 h after transfection. The time-lapse imaging (frame rate of 10 s) was done in an open coverslip holder in a temperature-controlled incubator built around the spinning disc microscope. Images were recorded using a C-Apochromat objective (63×/1.20W Korr M27) and a QuantEM 512SC EMCCD camera. Depending on cellular morphology, images were taken as z-stacks of 5–10 images (z-intervals of 0.31 µm). Exposure times were limited to 50–200 ms, and laser power was kept at 3–10%. Images were processed using ZEN2012, Imaris, ImageJ, and Adobe Photoshop.

Quantitative evaluations of the frequency of dendritic protrusion formation were done in dendritic segments of ≥20 µm length and in periods of 15 min. Protrusion starts reaching at least 1 µm in length were counted. The frequency of dendritic protrusion starts was quantitatively expressed as starts per 20 µm dendrite length and min.

### Structural predictions and representations

Ankrd24 ankyrin repeats are schematically shown according to UniProt. Low-scoring ankyrin repeats were analyzed manually by alignment (Clustal Omega; https://www.ebi.ac.uk/jdispatcher/msa/clustalo?stype=protein) and comparison with established consensus sequences for ankyrin repeats (Mosavi et al., 2002; Mosavi et al., 2004; Sedgwick and Smerdon, 1999).

Structural predictions were done using AlphaFold3 (multiple runs each request; https://alphafoldserver.com).

Helical wheel analyses and representation were done with HELIQUEST (https://heliquest.ipmc.cnrs.fr/cgi-bin/ComputParamsV2.py).

Coiled-coil domain predictions to define the coiled-coil areas of Ankrd24 were done with COILS (RRID:SCR_008440; tool no longer available via Expasy).

### Statistics and reproducibility

No explicit power analyses were used to compute and predefine required sample sizes. Instead, all neuronal analyses were conducted by systematic sampling of transfected cells across coverslips to avoid any bias in cell imaging.

All quantitative morphological data sets were obtained from at least three independent neuronal preparations obtained from a full litter of mixed-sex embryos each (∼14 each cohort of embryos). As the pooled preparations were done E18, the litters were not only from independent breedings but also from independent mothers.

Each of these assays contained several independent coverslips for each condition of transfection and immunostaining. For each condition, a minimal *n* number of 10 individual neurons per assay was aimed for. Higher *n* numbers yielding from the systematic imaging were accepted. Outliers or strongly scattering data were considered as reflecting the biological variance and were thus not excluded from the analyses. The absolute data of all parameters from all assays were first evaluated for the variances between assays and for the cell biological variances between the different cells in the dissociated neuronal cultures. The results of the variance analyses considering all functional data sets included in this study were as follows: morphological data spread between cells, 206–1,033% depending on dendritic parameter; corresponding data spread between preparations of cells as source for the establishment of dissociated primary hippocampal cultures, which later were used for the different transfections: 5–50%. The variances between cells by far exceed the variances from assay to assay. Because of the rather minor variance between assays and the high range of biological variances between cells (visualized by color-coded dots for dendritic branch points, dendritic terminal points, and dendritic length), the individual cellular data was considered as biological variance (*n*). When space considerations permitted this, quantitative evaluations are shown as bar/dot plots to fully and transparently highlight the cellular variance. In alternative visualizations of functional data, so-called SuperPlots (Lord et al., 2020) for dendritic branch points, dendritic terminal points, and dendritic length were added. These describe the biological data scattering as assay-encoding colored dots together with a mean of the assay-to-assay variances (shown as larger, differently colored dots on top of the small puncta representing the cellular data; the assay mean is presented as a black line). Branch depth analyses are shown as bar plots. The analyses of the discontinuous Sholl circle arrangements (5 µm distance each) are shown as bar plots and additionally also as continuous line plots.

All work with liposomes was done as independent reconstitutions with at least three independent preparations of liposomes from Folch fraction I liposomes. The data is presented as bar/dot plots.

All *n* numbers of quantitative analyses are reported in the legends of the figures of the manuscript. As in more detail explained above, all *n* numbers either represent independent biological samples, i.e., transfected neurons (Fig. 4, Fig. 5, Fig. 6, Fig. 7, Fig. 10, Fig. S2, Fig. S3, and Fig. S5), dendritic segments analyzed for branch induction sites (Fig. 4 K), and liposomes in EM analyses of liposome diameters (Fig. 3; Fig. 9) or represent biochemical assays conducted, such as for the liposome binding assays (Fig. 2 N and Fig. 8, I–L) and the quantitative RNAi validation experiments (Fig. 5 A), if not indicated otherwise (SuperPlots show means of averages of different cell cultures used as source for the cells to be transfected). Technical replicates to minimize measurement errors were not useful because technical errors were small in relation to the biological/biochemical variances observed.

All additional data shown are representative examples (western blots, immunofluorescence pictures, and EM pictures). All nonquantitative experiments were reproduced at least two more times.

Quantitative data reported in the manuscript represent mean ± SEM throughout. All quantitative data sets are provided as bar and dot plot overlays, albeit especially in liposome shape analyses and in morphological analyses with their larger range, and the differences in the means are, in part, difficult to resolve. Sholl analyses and branch depth analyses with their many very narrow bars not being suitable for harboring individual data points are presented as multiple bar plots and additional line plots (Sholl analyses), respectively.

Normal data distribution and statistical significance were tested using GraphPad Prism 9 and 10 software (RRID:SCR_002798), respectively. The Kolmogorov–Smirnov, the D’Agostino and Pearson, as well as the Shapiro–Wilk tests were used to first check the data for normal distribution. If all data sets were distributed normally according to all applicable tests, an unpaired *t* test (2 conditions to compare) and a one-way ANOVA (≥3 conditions to compare), respectively, were used (parametric tests). For not normally distributed data, a Mann–Whitney test (2 conditions to compare) or a Kruskal–Wallis test (≥3 conditions to compare) were carried out as nonparametric tests. Two-way ANOVA examinations were carried out for variance analyses with two factors. Two-way ANOVA mixed-effects model (REML) was applied for additional statistical test of Sholl analyses. All multiple comparisons were followed by the respective posttests. The statistical tests employed are reported in the respective figure legends. All detailed numerical information as well as the results of the statistical tests applied are compiled in Data S1.

Statistical significances were marked by *p < 0.05, **p < 0.01, ***p < 0.001 and ****p < 0.0001 throughout. Note that *p *< 0.0001 (****) are too small to be reported by the software.

### Online supplemental material

Fig. S1 shows supplementary supernatant data related to the coprecipitation and coimmunoprecipitation experiments demonstrating the interaction of Ankrd24 with syndapin proteins shown in Fig. 1, B–E as well as individual immunofluorescence channels and line scans of the subcellular recruitment assays in COS-7 cells shown in Fig. 1, G–I and of the localization of Ankrd24 proteins in HeLa cells displayed in Fig. 2, I–K. Fig. S2 shows further validations of the Ankrd24 gain-of-function phenotypes promoting the formation of the dendritic arbor in developing neurons corresponding to Fig. 4, C–J by alternative analyses, including SuperPlot presentations and by displaying data subsets, for which axon exclusions were additionally validated by additional anti-tau counterstainings. Fig. S3 displays further validations of the identified Ankrd24 loss-of-function phenotypes on dendritic arborization corresponding to Fig. 5, B–H by alternative analyses, including SuperPlot presentations, by displaying data subsets, for which axon exclusions were additionally validated by additional anti-tau counterstainings, and furthermore by an employment of a second, independent RNAi. Fig. S4 shows immunoblots of cross-linking experiments with lysates of HEK293 cells overexpressing Ankrd24-GFP and mutants thereof, displays representative example images and 3D reconstructions of neurons corresponding to the quantitative analyses shown in Fig. 6 and presents immunoblots of coprecipitation analyses confirming that Ankrd24*^Δhelix^-GFP exhibits binding to the syndapin I SH3 domain comparable with Ankrd24*-GFP. Fig. S5 shows further validations and alternative visualizations of identified phenotypes by SuperPlots corresponding to Fig. 6 and demonstrating that Ankrd24 functions in dendritic arborization rely on Ankrd24’s syndapin-associating KRAP motif, on N-terminal N-Ank module functionality and on Ankrd24’s coiled-coil domain. Video 1 relates to the collection of MIPs of the 3D time-lapse imaging frames of liposomes decorated with purified, recombinant Ankrd24^1-285^-GFP shown in Fig. 3 A. Video 2 presents an example movie related to the quantitative evaluation of dendritic arbor development in primary hippocampal neurons transfected with GFP shown in Fig. 4 K. Video 3 presents an example movie related to the quantitative evaluation of dendritic arbor development in primary hippocampal neurons transfected with Ankrd24-GFP shown in Fig. 4 K. Source Data files show raw and complete Western blots and gels of all main and supplementary figures. Table 1 lists primers. Data S1 lists all numerical data and shows the results of the respective statistical test applied for all quantitative evaluations shown in the main and the supplementary figures.

## Supplementary Material

Review History

Data S1 lists all numerical data and shows the results of the respective statistical test applied for all quantitative evaluations shown in the main and the supplementary figures.

SourceData F1 is the source file for Fig. 1.

SourceData F2 is the source file for Fig. 2.

SourceData F4 is the source file for Fig. 4.

SourceData F8 is the source file for Fig. 8.

SourceData F10 is the source file for Fig. 10.

SourceData FS1 is the source file for Fig. S1.

SourceData FS4 is the source file for Fig. S4.

## Data Availability

All data generated or analyzed in this study are included in the manuscript and in the supporting supplementary files. Numerical source data are provided as supplementary information, too.
