# Supplementary material for: The N-Ank protein Ankrd24 shapes neurons and couples to syndapin and the F-actin promoter Cobl-like
Source: J Cell Biol. 2026 Oct 21;225(11):e202509074. doi: 10.1083/jcb.202509074 (PMC13712265; doi:10.1083/jcb.202509074)
Supplement: Supplementary file 1 [file jcb_202509074_review_history.pdf]

The N-Ank protein Ankrd24 shapes neurons and couples to syndapin and the F-actin promoter Cobl-like

Astha Jain, Maryam Izadi-Seitz, Yuanyuan Ji, Eric Seemann, Britta Qualmann, and Michael Kessels

Corresponding Author(s): Michael Kessels, Jena University Hospital - Friedrich Schiller University Jena

Review Timeline:

Submission Date:	2025-09-10
Editorial Decision:	2025-10-20
Revision Received:	2026-04-10
Editorial Decision:	2026-05-11
Revision Received:	2026-07-10
Editorial Decision:	2026-08-07
Revision Received:	2026-08-18

Monitoring Editor: Tobias Meyer

Scientific Editor: Gabriele Stephan

Transaction Report:

(Note: With the exception of the correction of typographical or spelling errors that could be a source of ambiguity, letters and reports are not edited. The original formatting of letters and referee reports may not be reflected in this compilation.)

DOI: https://doi.org/10.1083/jcb.202509074

October 20, 2025

Re: JCB manuscript #202509074

Michael Kessels
Jena University Hospital - Friedrich Schiller University Jena

Dear Dr. Kessels,

Thank you for submitting your manuscript entitled "The N-Ank protein Ankrd24 shapes neurons and couples to F-actin formation via syndapin and Cobl-like". Your manuscript has been assessed by expert reviewers, whose comments are appended below. Although the reviewers express potential interest in this work, significant concerns unfortunately preclude publication of the current version of the manuscript in JCB.

You will see that the reviewers are overall supportive, thinking that your manuscript provides high quality data and interesting findings towards the role of Ankrd24 in membrane shaping. However, they all voice several critiques. We think your manuscript could improve if you were able to address the reviewers' concerns, including those related to statistical analyses. The localization of endogenous Ankrd24 as well as more mechanistic insights underlying the identified protein-protein interactions should be elucidated as requested by reviewer #1. Particularly, all experimental or textual alterations suggested by rev#2 should be included in a revised manuscript. We further recommend addressing reviewer #3's comments, which might involve repeating a number of the liposome and neuronal culture experiments as well as co-labeling of your cultures to exclude axons from all your measurements.

Please let us know if you are able to address the major issues outlined above and wish to submit a revised manuscript to JCB. If so, please provide a revision plan, outlining how you plan to address the reviewers' concerns.

Note that a substantial amount of additional experimental data likely would be needed to satisfactorily address the concerns of the reviewers. The typical timeframe for revisions is three to four months. If you anticipate any difficulties in meeting this aforementioned revision time limit, please contact us and we can work with you to find an appropriate time frame for resubmission. Please note that papers are generally considered through only one revision cycle, so any revised manuscript will likely be either accepted or rejected.

If you choose to revise and resubmit your manuscript, please also attend to the following editorial points. Please direct any editorial questions to the journal office.

GENERAL GUIDELINES:
Text limits: Character count is < 40,000, not including spaces. Count includes title page, abstract, introduction, results, discussion, and acknowledgments. Count does not include materials and methods, figure legends, references, tables, or supplemental legends.

Figures: Your manuscript may have up to 10 main text figures. To avoid delays in production, figures must be prepared according to the policies outlined in our Instructions to Authors, under Data Presentation, https://jcb.rupress.org/site/misc/ifora.xhtml. All figures in accepted manuscripts will be screened prior to publication.

IMPORTANT: It is JCB policy that if requested, original data images must be made available. Failure to provide original images upon request will result in unavoidable delays in publication. Please ensure that you have access to all original microscopy and blot data images before submitting your revision.

Supplemental information: There are strict limits on the allowable amount of supplemental data. Your manuscript may have up to 5 supplemental figures. Up to 10 supplemental videos or flash animations are allowed. A summary of all supplemental material should appear at the end of the Materials and methods section.

Please note that JCB now requires authors to submit Source Data used to generate figures containing gels and Western blots with all revised manuscripts. This Source Data consists of fully uncropped and unprocessed images for each gel/blot displayed in the main and supplemental figures. For assays performed using capillary electrophoresis and/or immunoassay-based detection, authors should instead provide the electropherogram graph(s) for each experiment, plotting fluorescence/chemiluminescence intensity vs. molecular weight/size. Please be sure to provide one Source Data file for each figure gels, blots, and/or capillary electrophoresis assays along with your revised manuscript files. File names for Source Data figures should be alphanumeric without any spaces or special characters (i.e., SourceDataF#, where F# refers to the associated main figure number or SourceDataFS# for those associated with Supplementary figures). For traditional gels and blots, the lanes of the gels/blots should be labeled as they are in the associated figure, the place where cropping was applied should be marked (with a box), and molecular weight/size standards should be labeled wherever possible. For capillary electrophoresis

assays, each trace in the graph should be color-coded and labeled to indicate which protein, gene, or sample is being measured (please try to avoid red/green combinations to accommodate our color-blind readers).

Source Data files will be made available to reviewers during evaluation of revised manuscripts and, if your paper is eventually published in JCB, the files will be directly linked to specific figures in the published article.

Source Data Figures should be provided as individual PDF files (one file per figure). Authors should endeavor to retain a minimum resolution of 300 dpi or pixels per inch. Please review our instructions for export from Photoshop, Illustrator, and PowerPoint here: https://rupress.org/jcb/pages/submission-guidelines#revised

If you choose to resubmit, please include a cover letter addressing the reviewers' comments point by point. Please also highlight all changes in the text of the manuscript.

Regardless of how you choose to proceed, we hope that the comments below will prove constructive as your work progresses. We would be happy to discuss them further once you've had a chance to consider the points raised. You can contact the journal office with any questions at cellbio@rockefeller.edu.

Thank you for thinking of JCB as an appropriate place to publish your work.

Sincerely,

Tobias Meyer
Monitoring Editor
Journal of Cell Biology

Gabriele Stephan
Scientific Editor
Journal of Cell Biology

Reviewer #1:

Jain et al. investigate the functions of the protein Ankrd24 in neurons. They suggest that this protein regulates the formation of dendrite complexity and branching. This is extremely important as the complex morphology of neurons is central to their ability to assemble neural circuits. They first convincingly demonstrate that Ankrd24 is a member of the N-Ank superfamily. They then show using liposomes that it can induce the formation of small vesicles, suggesting its membrane bending abilities. They also convincingly demonstrate that overexpression (GOF) or reduction (LOF) of Ankrd24 results in a phenomenon of more or fewer dendrites and dendritic branches, respectively. This appears to work through syndapin and the actin modifier Cobl. The experiments are highly detailed and represent a great deal of molecular work. Nevertheless I have several comments/concerns that should be addressed before this paper is ready for the Journal of Cell Biology:

1. How do the authors propose that the specific phenomenon of increased dendritogenesis occurs when the overexpressed Ankrd24 is present everywhere in the neuron? The authors should show where endogenous Ankrd24, syndapin, and Cobl are found.
2. All experiments are in vitro. While the authors use primary neuronal culture, there was no effort to determine if these effects can also be seen in the brain.
3. While the authors have clearly established protein-protein interactions and necessary interactions for the phenomena, the mechanistic details remain vague.
4. I am also concerned that in some experiments the authors may have engaged in pseudoreplication to establish significance (not necessarily intentionally, this reviewer has noticed this often happens in some studies using cultured neurons) - effect sizes may be smaller than reported, and in vitro experiments such as these are notoriously difficult. It seems that the authors use individual neurons as independent biological replicates. This is incorrect - if I have misunderstood this, then the authors can correct me. See for example Figure 5, D-H. The authors should have separate cultures and culture dishes for each experiment. For example, two neurons in a given culture dish that overexpressed Ankrd24 would be considered technical replicates, not biological replicates. in some cases, the authors have only performed two independent neuronal preparations. While I commend the authors for clearly stating what they did, two independent neuronal preparations does not allow for statistical analysis. Similarly, results presented in Fig. 4 D-H - these are from 4 'independent coverslips' from 2 'independent' neuronal preparations. This means 2 coverslips from each culture. The two coverslips are technical replicates, while the cultures are biological replicates. I think the authors should carefully consider their statistical comparisons again.

Reviewer #2:

Manuscript by Jain et al. (2025), for consideration by the JCB

In their manuscript, Jain et al. explore the role of Ankrd24 in dendritic arborization of cultured primary hippocampal neurons and they characterize its membrane sculpting functions through in vitro approaches and its interactions with the F-BAR protein SdpI/PACSIN1 and Cobl-like. Ankrd24 is an ankyrin repeat protein belonging to the N-Ank family that was previously introduced by the same group (Wolf et al., 2019, NCB). The main findings of the current study include that Ankrd24 directly interacts with SdpI and through SdpI with Cobl-like. Cobl-like has been implicated in promoting F-actin formation and this ternary complex is shown to promote dendritic arborization of neurons in vitro. Furthermore, in reconstitution experiments, they find that the N-terminal portion of Ankrd24 is sufficient to drive membrane sculpting, however, Ankrd24 cooperates with SdpI and Cobl-like during dendritic arborization.

In a related study, the same group recently characterized Ankycorbin, another member of the N-Ank family of ankyrin repeat proteins (Izadi et al., 2023, JCB), which also promotes dendritic arborization. Ankycorbin and Ankrd24 share the same overall domain structure with an N-terminal N-Ank domain, a KRAP motif mediating interaction with the SH3 domain of SdpI, and a C-terminal coiled coil domain. Ankycorbin's function in neuronal arborization was also found to depend on Cobl-like and its interaction with SdpI. Thus, the concept of two curvature-sensing/inducing proteins cooperating in dendritic arborization as well as the invovlement of Cobl-like had been introduced previously.

Ankrd24 is known to play a structural role in stereocilia, through interaction with the actin binding and bundling protein TRIOBP (Krey et al., 2022, JCB). This study also characterized the membrane binding of Ankrd24, its ability to self-associate, and to form aggregates.

Thus, not all of the findings presented in the current manuscript are surprising or conceptually novel. However, this carefully conducted study potentially contributes significantly to the understanding of how both Ankrd24 and N-Ank proteins function in neurons, through a broad range of methodologies and using both in vitro and cell culture models. Below, I list several points that should be addressed during a revision.

1. The title "The N-Ank protein Ankrd24 shapes neurons and couples to F-actin formation via syndapin and Cobl-like" raises the expectation that a relationship between Ankrd24 and F-actin formation is directly demonstrated. However, Ankrd24-dependent F-actin formation is not studied in this manuscript.

2. Ankrd24(1-285) vs Ankrd(1-1041). Work with GUVs/LUVs and liposomes as well as the interaction studies of the ternary complex with SdpI and Cobl-like were done using the N-Ank module of Ankrd24, i.e. AA 1-285 of the 1041 AA protein. This N-terminal fragment is sufficient to bind to liposomes, to sculpt membranes, to bind to Sdp1 on liposomes, and to form a ternary complex with SdpI and Cobl-like. The conclusion then is that Ankrd24 functions through its interactions with SdpI and Cobl-like. If so, Ankrd24(1-285) should mediate gain-of-function in dendritic arborization and rescue si-Ankrd24. Of the many conditions tested, these are missing in the current manuscript. The result would be important because it would highlight a potential role of the coiled-coil region and its interaction with potential other binding partners.

3. Previous work on Ankycorbin and Ankrd24. Given the similarity of the findings for Ankrd24 in the current manuscript and for Ankycorbin in the recent study from the same group (Izadi et al., 2023, JCB), it was surprising that the parallel findings were not thoroughly discussed in the discussion section. What are their functional similarities/differences, is there functional redundancy, how do they differ in sequence/domain structure, what is known about their expression patterns during neurodevelopment? It was also surprising that the findings from Krey et al., 2022, JCB on Ankrd24, were not mentioned in the introduction, such as N-Ank domain-dependent membrane association, coiled-coil-dependent self-association and aggregation, as well as interaction with the actin binding and bundling protein TRIOBP ("Knowledge of Ankrd24 properties and functions are very limited").

4. Membrane sculpting by Ankrd24. From enrichment on highly curved surface regions on GUVs/LUVs in Fig. 3A and Fig. 8M,N, it is concluded that Ankrd24(1-285) alone or with SdpI can sculpt membranes. To come to this conclusion, it would be necessary to demonstrate that these proteins are truly enriched over a curvature-insensitive control membrane binding protein. It is possible that regions with smaller vesicular protrusions harbor more membrane, such that uniform membrane binding of Ankrd24(1-285) and SdpI could appear as an enrichment. Clarifications are furthermore needed for what is happening in Fig. 3A and Fig. 8M,N: Are small vesicles truly budding off larger vesicles, can coalescence over time be firmly excluded? Including movies could be helpful, and a description of a model for how the budding would occur. Ankrd24(1-285) should not be able to self-associate - what is the model for how Ankrd24 can overcom the energy barrier to create budding of a new vesicular structures? Do Ankrd24 and SdpI form patterns of enrichment along neurites and during branch initiation?

5. The authors state (lines 364-366) that a mutation in the SH3 domain of SdpI prevents the interaction between SdpI and Ankrd24 (used in Fig. 9G), however, results demonstrating this are not provided.

6. Statistics. The same control data appear to have been used in multiple figure panels across figures and included in statistical analysis. The patterns of the individual datapoints suggest this:

The same scrRNAi data may have been used in:
- Fig. 5D and S3C
- Fig 5E and S3D
- Fig. 5F and S3E
The same GFP+scrRNAi and Ankrd24-GFP+scrRNAi may have been used in:
- Fig. 7E and 10B
- Fig. 7F and 10C
- Fig. 7G and 10D
One potential issue with doing this is that it may violate assumptions of the one-way ANOVA/post hoc tests, i.e. independent sampling.

From data shown in Fig. 10, it is concluded that targeting Cobl-like by siRNA suppresses Ankr24-GFP-induced neuromorphogenesis. However, of the 5 parameters tested, Ankr24-GFP expression did not cause a gain of function/statistically significant increase in two of them, the total dendritic tree length (Fig. 10D) and the Sholl analysis (10F). It is unclear then what is suppressed if there is no effect to suppress. The description and the discussion of the results should reflect this (similar issue in Fig. 7G). The lack of effect of Ankr24-GFP overexpression on these parameters furthermore is inconsistent with similar experiments, although without scrRNA, shown in Fig. 4F, H.

Minor:
- Line 479 "time-lapse"
- Fig. 4I: a supplementary video could be shown to illustrate the effect.
- Line 490: "Consistently, 8 out of 201 protein sequences identified in in silico screens with the consensus K-[RAGS]-[RKQ]-A-P-[PLAS]-PP (Izadi et al., 2023; Ji et al., 2024; Tröger et al., 2025), encoded for Ankrd24 proteins." - "N-Ank" instead of "Ankrd24", and are the citations correct?
- In the main text, references to panels of Fig. 2 need to be updated to match the current figure layout. Fig. E should be F, F should be G, etc.

Reviewer #3:

Review of Jain et al.

This study focuses on the protein Ankrd24 and how it's membrane binding and bending abilities contribute to dendritic development of hippocampal neurons. Ankrd24 is a relatively understudied protein, making this study novel. The Kessels and Qualmann group are strong cell biologists and have published extensively on membrane and actin associated proteins. This study, combining several complementary techniques, provides an intriguing set of structure-function data outlining how this protein, acting in conjunction with syndapin and Cobl-like, sculps convex membrane and functions in dendrite outgrowth and branching. Unfortunately, there are two major issues that detract significantly from the data that is presented. First, the authors repeat all the neuronal experiments and most of the liposome experiments in the paper only once (i.e. 2 independent neuronal preparations). This reviewer believes that the MINIMIM times experiments should be repeated is twice, yielding three independent data sets. The fact that most of the experiments in the paper were only repeated once compromises the entire study. Second, all the data on dendritic outgrowth and branching (Figs. 4-7, 10 and S2/S3) is additionally compromised because the authors only use MAP2 labeling on DIV6 cultures to draw their conclusions about dendrite development after knockdown of Ankrd24 and overexpression of full-length protein and deletion mutants. At DIV6 MAP2 expression is not totally excluded from axons and the authors show on most of their dendritic tracings very long, thin processes that are clearly axons. Thus, at this age in culture the authors would have to label for MAP2, as they have done, and co-label with a Tau1 or neurofilament antibody, because these proteins are concentrated in axons. Only this type of labeling will allow them to be able to exclude axons from their measurements of dendrites. Together, these two major weaknesses would require repeating most of the liposome and neuronal culture experiments again and use a more thorough labeling of cultures to exclude axons from all their measurements. Additional comments below, in relative order of importance, would help strengthen a resubmitted manuscript as well.

- The authors do not show the subcellular distribution of Ankrd24 in neurons anywhere in the manuscript. Ankrd24-GFP is shown in Fig. 4C, but it just looks like GFP. If it is a membrane bending protein shouldn't it be associated with vesicles, tubules and/or the plasma membrane?

- The authors do not speculate or provide a model for how Ankrd24 could mechanistically enhance dendrite development. Are there Ankrd24 coated vesicles that are transported to growing dendrites?

- Each Scholl curve and dendrite segment graph should be shown as line graphs +/-SEM (instead of bar graphs) and the line (all the measurements from a single cell) should be compared to one another. Individual lengths and branch depths should not be compared to one another as the authors have done. The comparisons shown for each length (the large tables and lines above individual bars) are meaningless.

- With every deletion being unable to rescue dendrite growth one wonders if the authors are just producing proteins that are not folding properly or are being degraded. The data would be much more convincing if: 1) images of GFP are shown indicating similar levels to WT ankrd24-GFP, 2) they show that a point or multiple point mutant (rather than a deletion) also doesn't rescue and 3) they show one type of deletion that does rescue.

- There still looks like about 50% interaction with the delta-KRAP Ankrd24 in the first blot in Fig. 1C (and Fig. S1A), but no co-IP in the second blot. Please explain such an obvious discrepancy.

- Fig. S1B-D are the same cells in Fig. 1E-G, just showing separate channels and a line scan. Fig. 1E-G should be removed and replaced by Fig. S1B-D. Also, the full length Ankrd24 looks a lot less specific than the 1-285 fragment regarding recruitment to Mito.

- The authors indicate in Fig. 9G that the liposomes resulting from the Ankrd24(1-285)/syndapin I(SH3 mutant) is statistically indistinguishable from control liposomes. The images (9A and 9F) and graph provided (9G, first and last bars) are not consistent with that statement. There seems to be many smaller liposomes in 9F(arrows) compared to 9A and the means also seem quite different in the bar graph.

- The construct Ankrd24(10-285) is a good negative control and the data in Fig. 2K-M is convincing. Why wasn't Ankrd24 (10-285) used as a negative control in the differential centrifugation or expression in HEK cells?

- Line 246-48 - It is unclear why the shRNA resistant form of Ankrd24 (Ankrd24*) is only included in a supplemental figure and not a regular figure. This is an important piece of data.

- The authors use "clearly" 17 times throughout the manuscript. This modifier should be used sparingly as it is up to the reader to determine whether the authors showed something clearly. The authors also refer to the fact that when the Ankrd24(1-285) was analyzed it was "irritating" that the proposed amphipathic helix was lacking. Although I am sure it was, it does not seem to be appropriate to include this feeling in a scientific manuscript.

- Regarding Fig. 1D, does the full length Ankrd24 bind syndapin I directly or just the 1-285 fragment?

- Ankrd24 is never defined as ankyrin repeat domain-containing protein 24 in the manuscript.

- Line 159-67 - the panel letters of Fig. 2F-M are mislabeled in the text.

- Line 206 - Please add the age of the rat(s) from which the brain lysate was prepared.

Detailed revision plan / our point-to-point responses:

Reviewer #1:
Jain et al. investigate the functions of the protein Ankrd24 in neurons. They suggest that this protein regulates the formation of dendrite complexity and branching. This is extremely important as the complex morphology of neurons is central to their ability to assemble neural circuits. They first convincingly demonstrate that Ankrd24 is a member of the N-Ank superfamily. They then show using liposomes that it can induce the formation of small vesicles, suggesting its membrane bending abilities. They also convincingly demonstrate that overexpression (GOF) or reduction (LOF) of Ankrd24 results in a phenomenon of more or fewer dendrites and dendritic branches, respectively. This appears to work through syndapin and the actin modifier Cobl. The experiments are highly detailed and represent a great deal of molecular work. Nevertheless I have several comments/concerns that should be addressed before this paper is ready for the Journal of Cell Biology:

We thank the reviewer for his/her overall positive assessment of our work. After discussions with the editors and working out a respective revision plan, we are happy to in the following report that we have successfully completed all points of the revision plan and thereby have addressed the comments/concerns mentioned by the reviewer in detail.
We also added some experimentation beyond the revision plan discussed with the editors to substantiate the findings of our work even further. This predominantly concerns new data obtained from further 3D-time-lapse analyses of developing neurons and (quantitative) electron microscopic analyses of Ankrd24 N-Ank modules on membranes with different curvatures.

1. How do the authors propose that the specific phenomenon of increased dendritogenesis occurs when the overexpressed Ankrd24 is present everywhere in the neuron? The authors should show where endogenous Ankrd24, syndapin, and Cobl are found.

As discussed with the editors, we have added a model showing Ankrd24 and the ternary complex of Ankrd24, syndapin I and Cobl-like at dendritic branches (please see newly added Figure 10J) and added more explanation about the mechanisms of Ankrd24-mediated dendritogenesis, i.e. how to bring about discontinuity in membrane topology and coupling to F-actin formation to particular sites along dendrites and how to thereby promote dendritic branch induction to the revised manuscript.
The localizations of syndapin I and of Cobl-like included in these scenarios have been demonstrated before. Both proteins are clearly detectable in dendrites and at dendritic branch induction sites (Izadi et al., 2018; Izadi et al., 2023). **During our revision work we added experimentation showing that the endogenous Ankrd24 protein is clearly present in dendrites, too (please see newly added Figure 4A).**
Beyond the revision plan discussed with the editors, we have furthermore added 3D-time lapse imaging of developing neurons that revealed that also Ankrd24-GFP occurred at nascent dendritic branch sites (please see revised Figure 4L).
In line with Ankrd24's promotion of convex membrane bending (**Figure 3**), Ankrd24 did hereby accumulate at the base of a forming protrusion, which (in the direction of the forming branch) represents a convex membrane area (please also see model in **Figure 10J**). Both aspects furthermore are in line with the finding that Ankrd24 promoted an increase in the induction of protrusion starts, as observed in quantitative 3D live analyses of dendritic arbor formation (**Figure 4K**; **please also see newly added Supplementary videos 2 and 3 for illustration**).

2. All experiments are in vitro. While the authors use primary neuronal culture, there was no effort to determine if these effects can also be seen in the brain.

All experiments we show are in cultures of developing primary hippocampal neurons. For physiologists this maybe is considered as *in vitro*, whereas for biochemists and cell biologists, work in a living entity (= cell) is usually considered as *in vivo* and is distinguished from molecular work and reconstitution studies with purified components (considered as *in vitro*), while work at the tissue and/or whole animal level is then considered as *in situ*.
While this is mere semantics, the reviewer will be aware of the fact that the brain itself does not easily allow for acute loss- and-gain of function studies during relatively early development in combinations with several other loss-of-function paradigms and in combination with phenotype rescue analyses using different mutants, as conducted in our study. The fact that our work is done in primary hippocampal neurons will therefore of course indeed remain a limitation of our study.
We thank the editor for having left this point out from the revision demands, as it is virtually impossible to experimentally address the molecular/mechanistic details of Ankrd24 functions we revealed in our study in the whole brain.

3. While the authors have clearly established protein-protein interactions and necessary interactions for the phenomena, the mechanistic details remain vague.

It is correct that the manuscript focused on the Ankrd24 side of the Ankrd24/syndapin I interaction by clearly identifying the syndapin binding interface (Ankrd24's +KRxAPxPP motif) and by the generation and functional analyses of an Ankrd24 mutant deficient for syndapin I binding (Ankrd24$^{\Delta KRAP}$). We acknowledge that the initially submitted manuscript fell short on explicitly demonstrating which mechanism is used for the interaction on the syndapin I side, did not show Ankrd24 during dendritic branch induction and also did not visualize the molecular mechanisms underlying the identified role of Ankrd24 in dendritic branch induction by some model to make the insights obtained visually accessible to readers.
We have therefore conducted additional experiments to work out more clearly the mechanistic details underlying the Ankrd24/syndapin I interaction in the revised version of the manuscript (please see revised Figure 1). We were indeed able to conclusively demonstrate that the Ankrd24 interaction is mediated by the syndapin I SH3 domain by coprecipitation experiments using a variety of syndapin I mutants including the syndapin I P434L mutant leading to a PxxP-motif binding-deficient SH3 domain (**please see newly added Figures 1D and S1B)**.
Together with the experiments that already were included in the previous version of the manuscript, these data now firmly demonstrate that the molecular mechanism of the interaction is a SH3/+KRxAPxPP motif association and that mutating syndapin I's SH3 domain is sufficient to disrupt Ankrd24/syndapin I complex formation.

Beyond the revision plan agreed on by the editors, we also added further data highlighting the role of Ankrd24 during dendritic branch induction of developing neurons by 3D time-lapse imaging (please see revised Figure 4L and the additionally added supplementary videos accompanying the quantitative panels Figure 4K). The additionally added data shows that Ankrd24 is present and accumulates at nascent dendritic branch sites (**Figure 4L; Supplementary Videos 2 and 3**). This is in line with the identified membrane-shaping of Ankrd24's N-Ank module (**Figure 2 and 3**), which mechanistically supports the formation of convex membrane surfaces, as they can be found at the cytosolic face of the plasma membrane at the base of a forming dendritic branch.

Dendritic branch induction coincides with transient actin filament formation at the base of a forming branch (Hou et al. 2015). **New experimentation we added during the revision shows F-actin-enriched Ankrd24 sites (newly added Figure 10A).**
Additionally added 3D time lapse analyses furthermore specifically shows dendritic branch induction sites marked by both Ankrd24-GFP and F-actin (detected by LifeAct-RFP) (please see newly inserted Figure 10B and C).
Besides the improved discussion of the revised manuscript, a model inserted into Figure 10 now more clearly summarizes the mechanisms of Ankrd24-mediated dendritic branch induction (please see newly added Figure 10J). The model also visualizes the fact not only the identified direct Ankrd24 interaction partner syndapin I but also the indirect binding partner Cobl-like occur at such sites (Izadi et al., 2023). The model furthermore highlights the finding that Ankrd24, syndapin I and Cobl-like form ternary complexes held together by (dimerized) syndapin I and that the base of dendritic protrusions offers convex membrane areas that are p

4. I am also concerned that in some experiments the authors may have engaged in pseudoreplication to establish significance (not necessarily intentionally, this reviewer has noticed this often happens in some studies using cultured neurons) - effect sizes may be smaller than reported, and in vitro experiments such as these are notoriously difficult. It seems that the authors use individual neurons as independent biological replicates. This is incorrect - if I have misunderstood this, then the authors can correct me. See for example Figure 5, D-H. The authors should have separate cultures and culture dishes for each experiment. For example, two neurons in a given culture dish that overexpressed Ankrd24 would be considered technical replicates, not biological replicates. in some cases, the authors have only performed two independent neuronal preparations. While I commend the authors for clearly stating what they did, two independent neuronal preparations does not allow for statistical analysis. Similarly, results presented in Fig. 4 D-H - these are from 4 'independent coverslips' from 2 'independent' neuronal preparations. This means 2 coverslips from each culture. The two coverslips are technical replicates, while the cultures are biological replicates. I think the authors should carefully consider their statistical comparisons again.

This seems to be a misconception. It may have not been entirely clear that the two independent cell cohorts, which were used for our determinations of the distinct parameters of individual cells in the dissociated primary neuron cultures, each covered a significant range of individual donor embryos, concretely, a complete litter. This means, the n numbers of analyzed cells (23-47 cells per experimental condition upon initial submission) already covered the variance of two separate cohorts of donor embryos (~28 embryos). **In order to prevent such irritations, we now explain this in more detail in the revised manuscript.**
Furthermore, reviewer 3 for formal reasons demanded that our work shall be improved by an additional reproduction of all functional data by yet another – i.e. a third – independent cohort of cells serving as pool for our different experimental manipulations of developing neurons to study dendritic arborization at the cellular level. **We have therefore repeated all our functional data again in our revision work (please see increased numbers of neurons evaluated for the subcellular, dendritic parameters in the revised Figures 4, 5, 6, 7 and 10 as well as in the revised Supplementary Figure S3). These additional efforts shall also address the concerns of reviewer 1.**

On general note, also legal issues need to be considered in the design of experiments and statistics. With already ~28 embryos in total - now expanded to ~**42 embryos in the revised Figures 5, 7, 10, and S3 and to ~70 (!) embryos in the revised Figures 4 and 6** due to demands to include further conditions (also to be done 3times) - and with the already **obtained very high statistical**

significances in our cell biological examinations (all *** or **** for the major phenotypes in the different gain-of-function, loss-of-function, rescue attempts and suppression studies) additional repetitions in form of further independent cohorts of donor embryos for our to be analyzed cells bear the risk of being considered as unnecessary killings of experimental animals. This would strictly be prohibited by the European Animal Welfare Law. At least, it would represent a violation of the internationally applied 3R principle and thereby also be in direct conflict with international animal welfare laws.

Finally, a short note to technical replications and the criticism of pseudoreplication originating from these:
The reviewer is of course right that technical replications do not qualify as additional n numbers and including them into statistical analyses would indeed represent a pseudoreplication. A technical replication of a morphometric analysis would be, if the IMARIS-based assessment of all morphological details of the dendritic tree would be done several times for the same sample, i.e. cell. The resulting error would be a technical error (therefore *technical replicate*) but not represent biological variance (n). In cell biological research, the biological variances of individual cells analyzed are of course usually much higher than the technical variances of the measurements. **We therefore explicitly stated in the Statistics and Reproducibility section of the Material and Method section that we do not do any technical replicates.**

In order to follow up on the reviewer's suggestions, we have furthermore evaluated examplarily functional data in the alternative manner suggested by the reviewer (please see Reviewer Figure 1). As the reviewer will see, the gain-of-function phenotypes identified in Figure 4 are also valid even if evaluated by using the means of cells per entire assays as n and averaging these values instead of those of individual cells (n thereby only 3-5 assays instead of 48-70 individual neurons) **(please see Reviewer Figure 1)**.
Displaying the data as bar/dot plots allows for comparing the range of data between the two different types of evaluation. The comparison (**please compare Reviewer Figure 1 with Figure 4**) clearly highlights that the biological variance of individual cells is indeed much higher (about factor 5) than the variance between the means of assays.

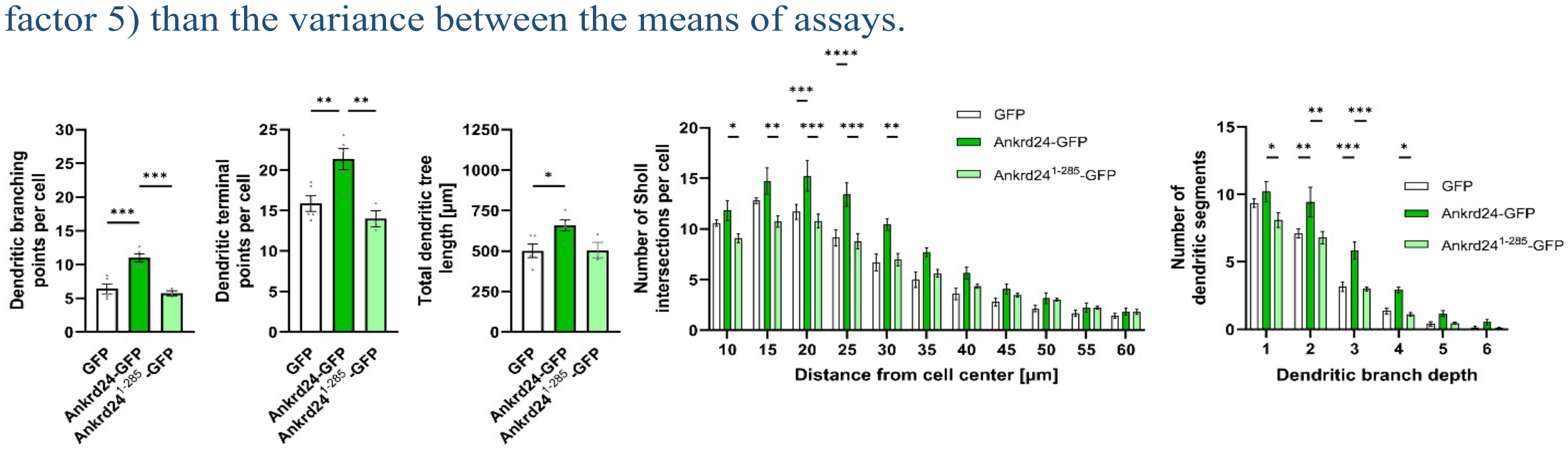

Reviewer Figure 1. Ankrd24 gain-of-function promotes the formation of the dendritic arbor in developing neurons.
Quantitatively evaluated dendritic parameters as in **Figure 4** of the revised manuscript but instead of focusing on individual cells as biological n for the cellular morphology analyses here depicted as mean of the means of the individual assays conducted (n = 3-5 assays). Note that the Ankrd24 gain-of-function phenotypes in dendritic branching are still valid also in this type of data evaluation.
Data, mean ± SEM shown as bar plots (two right panels) or bar/dot plots overlaid with individual data points (three left panels). Statistical significances, One-way ANOVA with Holm-Sidak posttest (three left panels) and Two-way ANOVA with Sidak posttest (two right panels). *$p < 0.05$; **$p < 0.01$; ***$p < 0.001$; ****$p < 0.0001$.

Reviewer #2:
Manuscript by Jain et al. (2025), for consideration by the JCB
In their manuscript, Jain et al. explore the role of Ankrd24 in dendritic arborization of cultured primary hippocampal neurons and they characterize its membrane sculpting functions through in vitro approaches and its interactions with the F-BAR protein SdpI/PACSIN1 and Cobl-like. Ankrd24 is an ankyrin repeat protein belonging to the N-Ank family that was previously introduced by the same group (Wolf et al., 2019, NCB). The main findings of the current study include that Ankrd24 directly interacts with SdpI and through SdpI with Cobl-like. Cobl-like has been implicated in promoting F-actin formation and this ternary complex is shown to promote dendritic arborization of neurons in vitro. Furthermore, in reconstitution experiments, they find that the N-terminal portion of Ankrd24 is sufficient to drive membrane sculpting, however, Ankrd24 cooperates with SdpI and Cobl-like during dendritic arborization.
In a related study, the same group recently characterized Ankycorbin, another member of the N-Ank family of ankyrin repeat proteins (Izadi et al., 2023, JCB), which also promotes dendritic arborization. Ankycorbin and Ankrd24 share the same overall domain structure with an N-terminal N-Ank domain, a KRAP motif mediating interaction with the SH3 domain of SdpI, and a C-terminal coiled coil domain. Ankycorbin's function in neuronal arborization was also found to depend on Cobl-like and its interaction with SdpI. Thus, the concept of two curvature-sensing/inducing proteins cooperating in dendritic arborization as well as the invovlement of Cobl-like had been introduced previously.

Ankycorbin indeed has been characterized as first, founding member of the N-Ank superfamily by Wolf et al., 2019 (*Nat. Cell Biol.*). Neither Wolf et al. (2019 *Nat. Cell Biol.*) nor Izadi et al. (2023 *J. Cell Biol.*) addressed whether and how ankycorbin as a syndapin binding partner may functionally and/or physically couple to Cobl-like - nor has any other ankycorbin study to our knowledge demonstrated this thus far.
A functional and physical coupling to the actin filament formation-promoting factor Cobl-like by ternary complex formation with an N-Ank protein, as Ankrd24, has solely been found and characterized by our study herewith submitted to *J. Cell Biol*. in revised form.

Ankrd24 is known to play a structural role in stereocilia, through interaction with the actin binding and bundling protein TRIOBP (Krey et al., 2022, JCB). This study also characterized the membrane binding of Ankrd24, its ability to self-associate, and to form aggregates. Thus, not all of the findings presented in the current manuscript are surprising or conceptually novel.
However, this carefully conducted study potentially contributes significantly to the understanding of how both Ankrd24 and N-Ank proteins function in neurons, through a broad range of methodologies and using both in vitro and cell culture models. Below, I list several points that should be addressed during a revision.
1. The title "The N-Ank protein Ankrd24 shapes neurons and couples to F-actin formation via syndapin and Cobl-like" raises the expectation that a relationship between Ankrd24 and F-actin formation is directly demonstrated. However, Ankrd24-dependent F-actin formation is not studied in this manuscript.

Cobl-like is a factor that strongly promotes the formation of actin filaments (Izadi et al., 2018) and clearly couples to Ankrd24 via syndapin.
In case the editors and all reviewers agree, *The N-Ank protein Ankrd24 shapes neurons and couples to syndapin and the F-actin promoter Cobl-like* (99 characters) could thus be used as alternative title of our work.

2. Ankrd24(1-285) vs Ankrd(1-1041). Work with GUVs/LUVs and liposomes as well as the interaction studies of the ternary complex with SdpI and Cobl-like were done using the N-Ank module of Ankrd24, i.e. AA 1-285 of the 1041 AA protein. This N-terminal fragment is sufficient to bind to liposomes, to sculpt membranes, to bind to Sdp1 on liposomes, and to form a ternary complex with SdpI and Cobl-like. The conclusion then is that Ankrd24 functions through its interactions with SdpI and Cobl-like. If so, Ankrd24(1-285) should mediate gain-of-function in dendritic arborization and rescue si-Ankrd24. Of the many conditions tested, these are missing in the current manuscript. The result would be important because it would highlight a potential role of the coiled-coil region and its interaction with potential other binding partners.

We agree with the reviewer that addressing the importance of the coiled coil domain was a major aspect missing in our work.
As the reviewer suggested, we therefore conducted additional functional work using Ankrd24$^{1-285}$. First, gain-of-function analyses clearly demonstrated that the coiled coil domain is important for Ankrd24-meidated dendritic arborization, as Ankrd24$^{1-285}$ did not lead to an statistically significant increase in any of the dendritic arborization parameters studied (please see revised Figure 4C-J). Rather it even had a minor, probably dominant-negative effect that became statistically significant for primary dendrite number **(please see branch depth analyses; revised Figure 4I)** and in the number of Sholl intersections for 10, 15 and 20 µm – findings that are most likely related to the primary dendrite reduction **(please see revised Figure 4J).**
Second, loss-of-function analyses with the respective rescue attempts by either wild-type protein and or different mutants – now also including Ankrd24$^{1-285}$, as the reviewer suggested – clearly unraveled that the coiled coil domain is critical for Ankrd24's role in dendritic arbor formation, as besides Ankrd24*$^{\Delta KRAP}$, Ankrd24*^{cc} and Ankrd24$^{\Delta helix}$ also Ankrd24$^{1-285}$ lacking the coiled coil domain was unable to rescue (please see revised Figure 6A-E).

3. Previous work on Ankycorbin and Ankrd24. Given the similarity of the findings for Ankrd24 in the current manuscript and for Ankycorbin in the recent study from the same group (Izadi et al., 2023, JCB), it was surprising that the parallel findings were not thoroughly discussed in the discussion section. What are their functional similarities/differences, is there functional redundancy, how do they differ in sequence/domain structure, what is known about their expression patterns during neurodevelopment? It was also surprising that the findings from Krey et al., 2022, JCB on Ankrd24, were not mentioned in the introduction, such as N-Ank domain-dependent membrane association, coiled-coil-dependent self-association and aggregation, as well as interaction with the actin binding and bundling protein TRIOBP ("Knowledge of Ankrd24 properties and functions are very limited").

Following the advice of the reviewer and within the length restrictions of JCB, the revised manuscript now discusses our work on ankycorbin in comparison to Ankrd24 in more detail.

The beautiful and insightful study done by Krey et al. on an Ankrd24 isoform in stereocilia of inner hair cells in the cochlea is covered in the introduction, in the results and in the discussion of our manuscript (Krey et al., 2022 *J. Cell Biol.*).
Krey et al. indeed showed that, in accordance to our N-Ank superfamily hypothesis (Wolf et al., 2019 *Nat. Cell Biol.*), Ankrd24 can self-associate via contributions of the coiled coil domain region. We confirmed the same by chemical crosslink studies for the longer variant of Ankrd24 in the brain and correctly referred to both our data (i.e. to **Figure S4**) and to Krey et al. (2022) in both the results section and also in the discussion of our manuscript.

A direct membrane association or a membrane insertion of Ankrd24 has not been demonstrated by Krey et al. (2022). Krey et al. showed a plasma membrane localization of Ankrd24 and a localization of Ankrd24 to stereocilia insertion points and to the F-actin-rich rootlets. Krey et al. (2022) also carefully and correctly described this as such and did not claim to have experimentally demonstrated the membrane binding, membrane insertion and membrane curvature induction properties unraveled in our study.
Since the reviewer specifically asked for additionally covering the TRIOBP/Tara interaction of Ankrd24 in stereocilia of inner hair cells of the cochlea (Krey et al., 2022), we added such information to the introduction of the revised manuscript and also inserted the respective literature references.
The number of Ankrd24 studies is indeed still very low and knowledge on its properties and functions are to our knowledge indeed limited to the analyses of a splice variant of Ankrd24 in inner hair cells in the inner ear (Krey et al., 2022; Kazemi et al., 2025). I**n the August issue of *J. Cell Biol.*, also Belyantseva et al., 2025 described the localization of Ankrd24 in inner hair cells. This new reference is now also included in our revised manuscript**) and to observations of some role in autophagy regulation in U251MG cells (Hu et al., 2023). Also reviewer 3 stated that Ankrd24 is understudied.
Since reviewer 2 thinks that in light of these few studies the "*very limited*" should rather be left out, **we now merely state in the revised manuscript that "*Knowledge of Ankrd24 functions is still sparse*". We hope that the reviewer will be more content with this wording.**

4. Membrane sculpting by Ankrd24. From enrichment on highly curved surface regions on GUVs/LUVs in Fig. 3A and Fig. 8M,N, it is concluded that Ankrd24(1-285) alone or with SdpI can sculpt membranes. To come to this conclusion, it would be necessary to demonstrate that these proteins are truly enriched over a curvature-insensitive control membrane binding protein. It is possible that regions with smaller vesicular protrusions harbor more membrane, such that uniform membrane binding of Ankrd24(1-285) and SdpI could appear as an enrichment.

Syndapin I is an F-BAR protein family member. In line with the BAR domain hypothesis (Peter et al., 2004 *Science*), syndapin I has been established as a membrane shaper (please e.g. see the reference Izadi et al. (2023) cited in the manuscript).
In general, we would like to note that the conclusion that Ankrd24 is able to sculpt membranes was not reached by the descriptive data shown in **Figure 3A** (or **Figure 8M,N)** but by the quantitative electron microscopy analyses shown in **Figure 3C-F** and **Figure 9. The revised manuscript should make this point clearer and should prevent any misunderstandings.** Our quantitative EM analyses of freeze-fractured liposomes incubated with Ankrd24$^{1-285}$ vs. controls clearly show that Ankrd24 promotes the formation of smaller, stronger curved membrane compartments.
New experimentation added to the manuscript furthermore now demonstrates that Ankrd24$^{1-285}$ can specifically be detected at liposome surfaces at ultrahigh resolution and quantitative analyses demonstrate that it prefers membranes with higher curvature. The Ankrd24$^{1-285}$-GFP densities at the membrane surfaces of smaller liposomes were determined to be more than 5times and highly statistically significantly ($p<0.0001$, ****) higher than at shallowly curved, large liposomes **(please see new Figure 3G-K).**
The analyses were done with freeze-fractured liposomes at the ultra-high resolution provided by TEM. The examined membrane areas were quantified. Putatively different amounts of membrane thus are not the concern in these analyses.

Clarifications are furthermore needed for what is happening in Fig. 3A and Fig. 8M,N: Are small vesicles truly budding off larger vesicles, can coalescence over time be firmly excluded? Including movies could be helpful, and a description of a model for how the budding would occur. Ankrd24(1-285) should not be able to self-associate - what is the model for how Ankrd24 can overcom the energy barrier to create budding of a new vesicular structures? Do Ankrd24 and SdpI form patterns of enrichment along neurites and during branch initiation?

As suggested by the reviewer, we included a Supplementary Movie comprising the frames shown in Figure 3A to the revised manuscript (**Supplementary Movie 1**). **Figure 8M,N** merely show the localizations of Ankrd24$^{1-285}$ and syndapin I and a colocalization of Ankrd24 and syndapin I, respectively, at liposomes at two different times of inspection. Light microscopy will not be able to resolve membrane sculpting processes when the curvatures induced by Ankrd24 reflect diameters of 300 nm in average and the majority to the resulting liposomes was determined to have diameters (far) below 100 nm (**Figure 3F**). For the identification and quantitation of membrane sculpting processes we would therefore like to refer the reviewer to our extensive work at the EM level described above (**please see Figure 3C-K and Figure 9 of the revised manuscript**).
As far as mechanistic considerations are concerned, the induction of local curvatures by Ankrd24 will lead to local self-organization process at such sites with better fitting membrane curvatures, **as not only alluded to in the discussion section but now also directly experimentally demonstrated** (**please see Figure 3G-K**). Sufficient amounts of Ankrd24$^{1-285}$ at liposome surfaces is sufficient to force liposomes into more strongly curved diameters, i.e. into structures with smaller diameters, as also experimentally demonstrated (please see our quantitative analyses of liposome diameters; **Figure 3, Figure 9**).
In developing WT neurons, Ankrd24 full-length is the effector. Full-length Ankrd24 is able to self-associate via its coiled coil domain (**Figure S4**) and furthermore interacts with syndapin I via its KRAP motif (**Figure 1**). Both molecular mechanisms can further increase local concentrations of Ankrd24. Syndapin I binding furthermore couples to local actin filament formation and thereby to the cytoskeletal forces created by actin filament formation processes (**please see discussion of the revised manuscript**).
Indeed, in new experimentation we added during the revision, we observed that Ankrd24$^{1-285}$ was neither able to elicit additional dendritic branches nor able to rescue any of the Ankrd24 loss-of-function phenotypes in dendritic arbor formation we identified (please see new data in Figure 4F-K and Figure 6A-E). Thus, besides the N-Ank domain and the syndapin I binding interface, also the more C terminal, self-association-competent coiled coil domain of Ankrd24 was critical for dendritic branch induction.
Enrichments of syndapin I along dendrites and even explicitly during the process of dendritic branch induction have been shown (Hou et al., 2015 *PLoS Biol*; Izadi et al., 2021 *J. Cell Biol*.). **We apologize that this was not described in sufficient detail in the previous manuscript. The revised manuscript was improved accordingly.**
As now explicitly demonstrated, also endogenous Ankrd24 is present in dendrites (Figure 4B). Similar to syndapin I distributions (e.g. Hou et al., 2015 *PLoS Biol*), **the staining was not continuous but showed varying signal intensities (Figure 4B).**
In newly added 3D-live analyses of developing neurons, we detected Ankrd24-GFP at forming dendritic branches. Ankrd24 hereby accumulated at the base of nascent dendritic branches during dendritic branch induction (please see newly inserted Figure 4L). Interestingly, Ankrd24 did not only coincide with transient F-actin accumulations at such sites but these sites also represent areas of convex membrane topology, i.e. the type of membrane topology Ankrd24 actively promotes (**please see newly inserted Figure 10A-C and also the added model Figure 10J**).

5. The authors state (lines 364-366) that a mutation in the SH3 domain of SdpI prevents the interaction between SdpI and Ankrd24 (used in Fig. 9G), however, results demonstrating this are not provided.

The reviewer is right, the previous version of the manuscript indeed fell short on explicitly experimentally demonstrating that the Ankrd24/syndapin I interaction is based on an SH3 domain interaction and that the syndapin I P434L mutation used indeed disrupts Ankrd24 association. **The revised manuscript now firmly demonstrates both of these facts (please see newly added Figures 1C and Figure S1B).**
The results clearly show that the SH3 domain is both sufficient (please note that syndapin I SH3 domain interacts) and required for the interaction (please note that syndapin I ΔSH3 domain does not interact). Furthermore, we now demonstrate that P434L mutation in both the SH3 domain context as well as in the context of the full-length protein disrupts the interaction with Ankrd24 completely **(Figure 1C; Figure S1B)**

6. Statistics. The same control data appear to have been used in multiple figure panels across figures and included in statistical analysis. The patterns of the individual datapoints suggest this:
The same scrRNAi data may have been used in:
- Fig. 5D and S3C
- Fig 5E and S3D
- Fig. 5F and S3E
The same GFP+scrRNAi and Ankrd24-GFP+scrRNAi may have been used in:
- Fig. 7E and 10B
- Fig. 7F and 10C
- Fig. 7G and 10D
One potential issue with doing this is that it may violate assumptions of the one-way ANOVA/post hoc tests, i.e. independent sampling.

The reviewer is right, **Figures 5** and **S3** use the same scr. RNAi data, as according to the 3R principle to reduce the use of experimental animals, these experiments have been conducted as one larger set of parallel analyses. The data sets were only split later in order to reduce the complexity of the main figure and to move the supporting data using RNAi#2 into the supplements. The data are thus fully biologically sound and of course also statistically sound.
We apologize for not having pointed out this fact in the legends of our initially submitted manuscript - normally we are doing this in all of our publications. We have added this information to the revised manuscript.
Likewise, the experiments shown in **Figures 7** and **10** (attempts to suppress the identified Ankrd24 gain-of-function phenotype in dendritic branching by either syndapin I RNAi (**Figure 7**) or by Cobl-like RNAi (**Figure 10**) were conducted as one extended set of data and only split into the syndapin I and the Cobl-like aspect subsequently for clarity of the manuscript. The control data of the extended data sets therefore had to be repeated in **Figure 10**. Also these data thus are fully biologically and statistically sound.
Also for these data sets, we have now explicitly mentioned that the corresponding control data of the assays are shown again for direct comparison.
Please note that the data sets in the revised manuscript are different from those shown in the initial version of the manuscript because the n numbers were further increased in the course of the revision work due to the demands of an additional replication and in part also because Ankrd24$^{1-285}$ was added as new condition in both the gain-of-function analyses (**Figure 4**) and in the loss-of-function/rescue experiments (**Figure 6**) and this required 3 new assays with all conditions tested

(**please see all functional analyses, i.e. Figure 4, Figure 5, Figure 6, Figure 7, Figure 10 and Figure S3**).

From data shown in Fig. 10, it is concluded that targeting Cobl-like by siRNA suppresses Ankr24-GFP-induced neuromorphogenesis. However, of the 5 parameters tested, Ankr24-GFP expression did not cause a gain of function/statistically significant increase in two of them, the total dendritic tree length (Fig. 10D) and the Sholl analysis (10F). It is unclear then what is suppressed if there is no effect to suppress. The description and the discussion of the results should reflect this (similar issue in Fig. 7G).
The lack of effect of Ankr24-GFP overexpression on these parameters furthermore is inconsistent with similar experiments, although without scrRNA, shown in Fig. 4F, H.

The reviewer is of course right. In case increases in dendritic branching do not reach statistical significance in combinatory experiments, claiming their suppression by Cobl-like RNAi would be inappropriate. Luckily, this was not the case for most of the dendritic parameters quantitatively analyzed in our analyses. The dendritic branch sites and the terminal points, i.e. the identified Ankrd24-mediated increase in dendritic branching (see former **Figure 4D,E; now Figure 4F,G**; both ****), are again clearly increased in the double-transfection setting required for Figure 7 and for Figure 10, in which phenotypes of course can be expected to be weaker **– a point that we explained in the revised manuscript, as the reviewer seemed irritated by this.** Importantly, both of these Ankrd24 gain-of-function phenotypes, increased dendritic branching and dendritic terminal points, were clearly suppressed by both Syndapin I RNAi (**Figure 7E,G**) and Cobl-like RNAi (**Figure 10E,F in the revised manuscript**).
The same is true for the dendritic branch depth analyses in former Figure 10D (**now Figure 10H**). Also for this dendritic evaluation, the Ankrd24 gain-of-function phenotype still was observed in branch depth level 3 (** $p>0.01$) and also for this parameter, the Ankrd24 effects were fully suppressed upon Cobl-like RNAi (**Figure 10H in the revised manuscript**).
The parameter *total dendritic tree length* in contrast, did indeed not reach statistical significance when addressed in a double-transfection paradigm (**Figure 7G; Figure 10D, now Figure 10F**). As stated by the reviewer, the observed reduction upon syndapin I and Cobl-like RNAi, respectively, coexpression therefore is indeed not meaningful in this particular case. The same is true for the trends of increases in Sholl intersections in the 20, 25, 30, 35 and 40 µm data sets that also failed to reach statistical significances in the coexpression paradigm of Figure 10 but just showed trends (**Figure 10I**). **We have reworded the revised manuscript accordingly to make sure that the changes to the dendritic arbor are not described too superficially but in sufficient detail for each informative individual dendritic parameter.**
In case the reviewer wants us to leave out the total dendritic length and Sholl analyses in Figure 10 because they are not showing all required statistical significances, we shall be happy to do so. Currently, we show these data sets for completeness.

Minor:
- Line 479 "time-lapse"

We thank the reviewer for noticing this typo. **We corrected it in the revised manuscript.**

- Fig. 4I: a supplementary video could be shown to illustrate the effect.

This refers to the protrusion start quantitation data (**now Figure 4K in the revised manuscript**). As obvious from the quantitative data, these events are of moderate frequency and require careful, close-up examinations of dendritic segments. **Following the advice of the reviewer, we nevertheless illustrated the quantitative analyses shown in Figure 4K by supplementary movies (Supplementary Movies 2 and 3 of the revised manuscript).**
The movies illustrate the quantitative data shown in **Figure 4K** quite well. The control shows 1 protrusion start/20 µm in 6 min (=0.167/min) versus Ankrd24 overexpression showing 2 starts (0.333/min) and thereby very well reflect the quantitative data in **Figure 4K**.

- Line 490: "Consistently, 8 out of 201 protein sequences identified in in silico screens with the consensus K-[RAGS]-[RKQ]-A-P-[PLAS]-PP (Izadi et al., 2023; Ji et al., 2024; Tröger et al., 2025), encoded for Ankrd24 proteins." - "N-Ank" instead of "Ankrd24", and are the citations correct?

We thank the reviewer for his/her note. This sentence seemed difficult to understand. **We improved the revised manuscript by simply leaving out this sentence, as it apparently was confusing and we had to shorten the text to meet the length restrictions of JCB.**

- In the main text, references to panels of Fig. 2 need to be updated to match the current figure layout. Fig. E should be F, F should be G, etc.

We sincerely apologize for this embarrassing mistake and very much thank the reviewer for noticing this. Upon insertion of scheme **Figure 2E** into **Figure 2**, apparently only the legend but not the main text had been adapted. Also the examination of the Ankrd24 delta helix mutation (Ankrd24$^{10\text{-}1041}$-GFP) in subcellular fractionations was not described and not referenced to (**Figure 10G in the revised manuscript**).
We added a description of Figure 2E and Figure 10G to the Results text and reported the following Figure 2 panel numbers correctly in the revised manuscript.

Reviewer #3:

Review of Jain et al.

This study focuses on the protein Ankrd24 and how it's membrane binding and bending abilities contribute to dendritic development of hippocampal neurons. Ankrd24 is a relatively understudied protein, making this study novel. The Kessels and Qualmann group are strong cell biologists and have published extensively on membrane and actin associated proteins. This study, combining several complementary techniques, provides an intriguing set of structure-function data outlining how this protein, acting in conjunction with syndapin and Cobl-like, sculps convex membrane and functions in dendrite outgrowth and branching. Unfortunately, there are two major issues that detract significantly from the data that is presented. First, the authors repeat all the neuronal experiments and most of the liposome experiments in the paper only once (i.e. 2 independent neuronal preparations). This reviewer believes that the MINIMIM times experiments should be repeated is twice, yielding three independent data sets. The fact that most of the experiments in the paper were only repeated once compromises the entire study.

We agree with the reviewer that all quantitative data in science should be verified by independent data sets and have of course done so. Apart from this, most of our data is quantitative in nature and is based on n numbers of biological/biochemical samples that in comparison to other studies in the literature are high or even very high.

A significant portion of the representative biochemical data we show in fact already did comply with the demand of the reviewer that all data does not need to be reproduced but needs to be double-reproduced by independent data or even independent sets of data (former Figure 1D, **4x**; Figure 2F-J, **3-4x**; Figure 3I, **3x**; Figure 8A,B,E,F,I,J, **3x**; Figure 10A, **3x**, Figure S4A-C, **3x**). **During the revision work, we conducted further replications of the representative data shown, where an n of 3 had not yet been reached but merely 2 independent experiments were conducted. All representative experimentation of the revised manuscript therefore now reflects at least 3 independent experiments.**

This also applies to the quantitative electron microscopic examinations of liposomes in Figure 3. **Yet another, a third independent set of in vitro-reconstitutions has been added during revision (Figure 3F of the revised manuscript**; now n=1261-1342 evaluated liposomes**).**
Likewise, in Figure 9, yet another set in vitro-reconstitutions has been added during our revision work (Figure 9G of revised manuscript; now n=629-847 evaluated liposomes**).**
Also, the quantitative evaluations of liposome binding (Figure 8A-L) are now all based on at least 3 different preparations of liposomes used for the individual reconstitutions. Previously, only the individual binding analyses shown in Figure 8I,J were based on 3 different liposome preparations as a source for the individual reconstitutions, precipitations and Western blot analyses, while the quantitative data sets shown in Figure 8K,L were based on 2 different liposome sources).

Our quantitative work included two of independent cohorts of animals used as sources for the primary cells required. These two cohorts of animals even came from two distinct origins (mothers). Yet, the reviewer asked for three such cohorts being used despite the fact that these two separate cell cohorts we studied each already covered a significant range of individual donor embryos, as each preparation of primary cells was from pooled hippocampi of a complete litter. This means, the n numbers of analyzed cells (previously 23-47 cells per experimental condition; now expanded further to 37-70 cells due to our revision efforts (see below)) already covered two separate donor animal cohorts of about 14 embryos each.

As it may not have been entirely clear that the cells quantitatively analyzed in total represented about 28 individual animals and not just one donor animal each (i.e. 2 in total, as the reviewer implied), we explicitly report this point in the Material and Method section of our revised manuscript.
Despite of this, the reviewer still demanded reproduction of all functional data by yet another cohort of cells/neuronal preparation and also yet another replication of all data shown in a representative manner the manuscript and wrote that such doubled-reproductions of all data is the major point of criticism, which precluded publication of our work and compromises our entire study. **We therefore increased the number of independent litters, from which the individual cellular n numbers for the dendritic morphology parameters come from, to the demanded number of at least three independent litters with about 14 embryos each (please see revised Figure 4, Figure 5, Figure 6, Figure 7, Figure 10 and Figure S3).**

We hope that in light of the above described already excellent and now even further expanded base of our data the reviewer will now be content with the data basis of our quantitative molecular/mechanistic and functional analyses.
We furthermore hope that in light of the already very high statistical significances already obtained in our cell biological examinations in the initially submitted manuscript (all major gain-of-function, loss-of-function, rescue and suppression phenotypes we identified already were $p< 0.001$, *** or even $p<0.0001$, **** and were therefore, not significantly improvable further by even higher n numbers) the demanded further repetitions in form of a third independent cohort of donor embryos for our to be analyzed cells our compliance during revision will not yet be considered as violation of the 3R principle or even of the animal welfare laws prohibiting unnecessary treatments and/or killings of experimental animals.

Second, all the data on dendritic outgrowth and branching (Figs. 4-7, 10 and S2/S3) is additionally compromised because the authors only use MAP2 labeling on DIV6 cultures to draw their conclusions about dendrite development after knockdown of Ankrd24 and overexpression of full-length protein and deletion mutants. At DIV6 MAP2 expression is not totally excluded from axons and the authors show on most of their dendritic tracings very long, thin processes that are clearly axons. Thus, at this age in culture the authors would have to label for MAP2, as they have done, and co-label with a Tau1 or neurofilament antibody, because these proteins are concentrated in axons. Only this type of labeling will allow them to be able to exclude axons from their measurements of dendrites.
Together, these two major weaknesses would require repeating most of the liposome and neuronal culture experiments again and use a more thorough labeling of cultures to exclude axons from all their measurements.

We thank the reviewer for his/her careful evaluations of our example images. MAP2 is a well-established marker for dendrites and tau is among the well-established markers for axons for both cultures of dissociated neurons and neurons in situ. We are interested in studying the dendritic compartment. Therefore, our analyses are based on the dendritic marker MAP2. Of course our standard protocols for dendritic analyses, which e.g. also include two recent *J. Cell Biol.* papers (Izadi et al., 2018; Izadi et al., 2023), therefore do exclude axons.
Due to the reviewer's comment, we carefully reevaluated each individual tracing done in the study by an experienced postdoc in the lab and made the required changes. We apologize for the in some cases incorrect execution of our analyses. The required corrections affected all experimental conditions in about the same manner and therefore did not significantly change any of our findings (please see results of new gain-of-function (Figure 4), loss-of-function (Figure 5

and Figure S3), rescue experiments with mutants (Figure 6) and dependencies from syndapin I (Figure 7) and Cobl-like (Figure 10), respectively.
We have replaced all exemplary images shown in the functional analyses by images from the new assays.
We have furthermore exemplarily checked the established literature procedure we are using by comparing our finding of an Ankrd24-mediated increase in dendritic arbor formation and expansion (first functional figure of our study; Figure 4) with the results of three new assays, which each used additional anti-tau stainings for marking the axons, as the reviewer suggested. The additional staining for the axonal marker tau was not affecting the dendritic analysis based on the dendritic marker MAP2. Also the anti-tau counterstained assays displayed the Ankrd24-mediated increase in dendritic branching, in dendritic terminal points, in dendritic length, increases in dendritic branch depth levels 2 and 3 and increases in Sholl intersections and showed the respective statistical significances for these phenotypes (Reviewer 3 Figure 1).

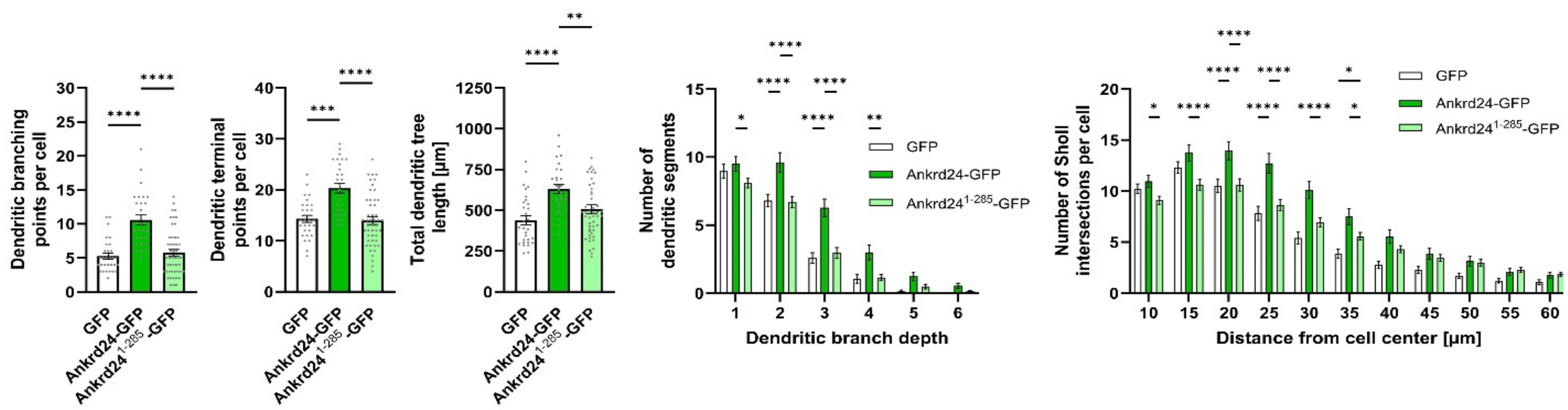

Reviewer 3 Figure 1. Ankrd24 gain-of-function promotes the formation of the dendritic arbor in developing neurons (related to Figure 4 of the revised manuscript).
Quantitative determinations of dendritic branching points, dendritic terminal points, total dendritic tree length, as well as dendritic branch depth and Sholl analyses related to data shown in **Figure 4** but solely based on anti-tau counterstained functional assays. Note that the Ankrd24-mediated effects in dendritic branching, i.e. the Ankrd24-mediated increase in dendritic branching, in dendritic terminal points, in dendritic length, increases in dendritic branch depth levels 2 and 3 and increases in Sholl intersections all are identified as in **Figure 4** in a statistically significant manner and therefore are valid also in this type of evaluation.
Data, mean ± SEM shown as bar plots (two right panels) or bar/dot plots overlaid with individual data points (three left panels). Statistical significances, Kruskal-Wallis test with Dunn's posttest (three left panels) and Two-way ANOVA with Sidak posttest (two right panels). $*p < 0.05$; $**p < 0.01$; $***p < 0.001$; $****p < 0.0001$.

Additional comments below, in relative order of importance, would help strengthen a resubmitted manuscript as well.

- The authors do not show the subcellular distribution of Ankrd24 in neurons anywhere in the manuscript. Ankrd24-GFP is shown in Fig. 4C, but it just looks like GFP. If it is a membrane bending protein shouldn't it be associated with vesicles, tubules and/or the plasma membrane?

During our revision work we added the information that the endogenous Ankrd24 protein is relatively abundant in dendrites (please see newly added Figure 4B).
The dendritic anti-Ankrd24 immunostaining is – in particular in more peripheral segments, which allow for better spatial resolution – a little bit discontinuous but we have no indications that this represents vesicles.
Considering the strong membrane association of Ankrd24 by amphipathic helix insertion (as e.g. demonstrated by subcellular fractionations; Figure 2), it can be assumed that a significant portion

of Ankrd24 is membrane associated. However, cytosolic versus plasma membrane-associated localizations in dendrites cannot be firmly distinguished by the resolution of light microscopy.
Newly added 3D-time-lapse analyses of Ankrd24 during dendritic branch induction showing transient accumulations of Ankrd24 at the base of a forming branch (**newly added Figure 4L in the revised manuscript**) would also be in line with these identified properties of Ankrd24, as such sites represent the convex membrane surfaces Ankrd24 is actively generating.

- The authors do not speculate or provide a model for how Ankrd24 could mechanistically enhance dendrite development. Are there Ankrd24 coated vesicles that are transported to growing dendrites?

We briefly outlined how with the properties and interactions we identified Ankrd24 may promote dendritic arborization (please see discussion). **We expanded and improved this discussion in the revised manuscript.**
We also thank the reviewer for the suggestion to include a model. **We provided a model that highlights the proposed mechanisms visually (please Figure 10J of the revised manuscript).**

Summarized, considering the basic, intrinsic properties of Ankrd24 we identified and Ankrd24's interactions with syndapin I and Cobl-like, we think that Ankrd24's plasma membrane association will promote local membrane topology changes by membrane curvature inductions. Such changes in membrane topology may then (by linkage to actin filament formation via the syndapin I interaction and by indirect coupling to the actin filament formation-promoting protein Cobl-like) be propagated further to form a dendritic branch.

- Each Scholl curve and dendrite segment graph should be shown as line graphs +/-SEM (instead of bar graphs) and the line (all the measurements from a single cell) should be compared to one another. Individual lengths and branch depths should not be compared to one another as the authors have done. The comparisons shown for each length (the large tables and lines above individual bars) are meaningless.

This probably is a misunderstanding. Instead of dendrite segment graphs, the reviewer probably means the dendritic branch depth analyses. This analysis yields distinct classes of dendritic arborization parameters and for example explicitly answers the question whether primary dendrites are affected (category branch depth 1) and this already contributes to defects in the dendritic arbor or whether the defects specifically occur at a certain branch level in the peripheral arbor and where (depth 2, 3, 4 and so on). Branch depth analyses thus provide biologically distinct categories, which of course therefore also need to be represented and statistically analyzed individually.

As far as Sholl analyses are concerned, this method provides spatial resolution in distinct areas of dendritic tree predefined by the Sholl circles set. The importance of an analysis at different distances from cell center is the reason why D.A. Sholl invented this type of quantitative analysis more than 60 years ago. Therefore, the situation at different distances of the dendritic tree is not meaningless. Instead, the method of Sholl highlights where exactly given phenotypes occur in the dendritic arbor.
Representations of Sholl analyses as bars and as lines can both be found in literature (e.g. see Monory et al., 2015; Li et al., 2018 for lines and e.g. Rinaldi et al., 2008; Bartelt-Kirbach et al., 2016; Macharadze et al., 2019 for bar representations). Per definition, however, Sholl analyses provide a row of data sets that are disconnected to each other, as the Sholl method used circles with predefined, larger distances to each other (often 5 or 10 µm). Because of this method-

immanent discontinuity, D.A. Sholl did not connect the data for each predefined distance (Sholl, 1963 *J. Anat.*). We usually prefer to do the same.
The type of data presentation chosen allows for clear side-by-side visualization of several conditions and their respective errors - even when the values are similar and would thus overlap in line representations (please e.g. compare **Figure 6**). Even detailed and multiple statistical significance analyses can be directly integrated into such graphs. Almost all of our analyses contain multiple conditions (please see **Figure 4, Figure 6, Figure 7, Figure 10, Figure S3 -** solely **Figure 5** is a comparison of merely two conditions), we therefore did not use line representations but preferred the side-by-side comparisons by bar plots. As we were furthermore interested in specifically in which parts of the dendritic arbor putative phenotypes may occur and are statistically significant, we furthermore preferred the more detailed statistical analyses shown in our figures.
In order to further improve the direct information content of the Sholl and the branch depth analyses, we integrated the statistical significance information directly into the diagrams in the revised manuscript. We hope that the reviewer finds these changes helpful and will acknowledge that our revised figures are informative.

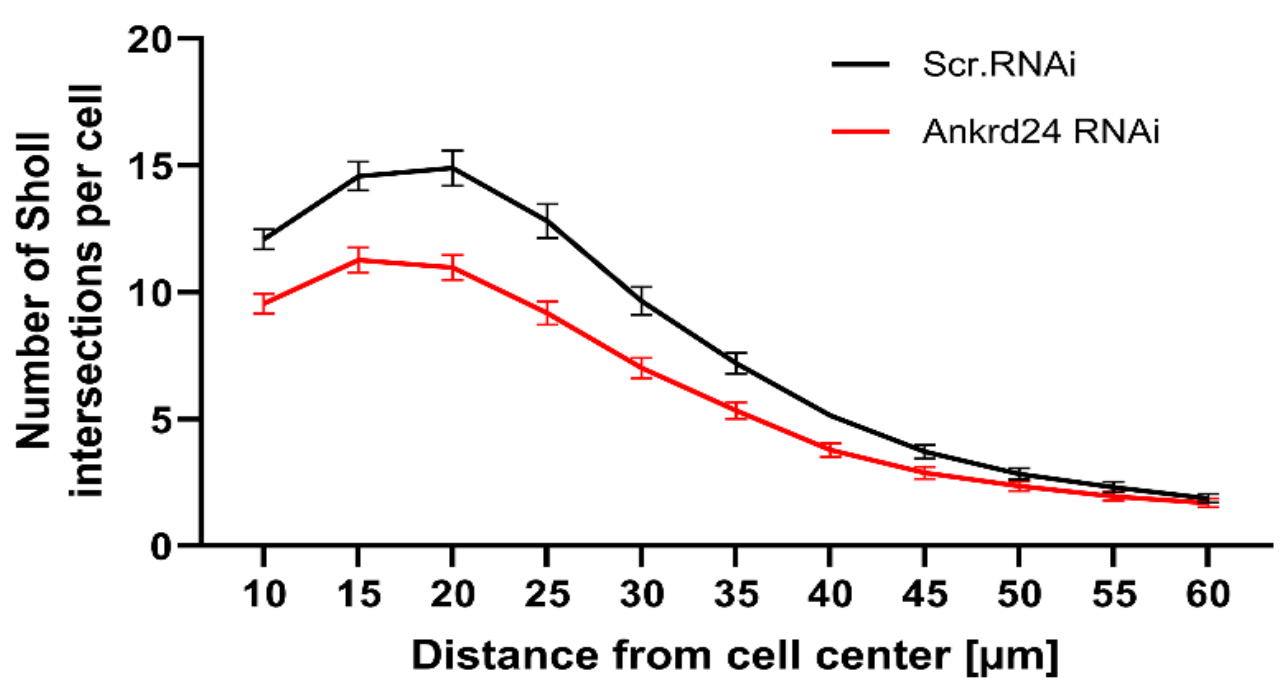

Two-way ANOVA	Ordinary				
Alpha	0.05				
Source of Variation	% of total variation	P value	P value summary	Significant?	
Interaction	1.596	<0.0001	****	Yes	
Row Factor	65.81	<0.0001	****	Yes	
Column Factor	3.445	<0.0001	****	Yes	
ANOVA table	SS	DF	MS	F (DFn, DFd)	P value
Interaction	469.6	10	46.96	F (10, 1078) = 5.902	P<0.0001
Row Factor	19370	10	1937	F (10, 1078) = 243.4	P<0.0001
Column Factor	1014	1	1014	F (1, 1078) = 127.4	P<0.0001
Residual	8577	1078	7.957		
Difference between column means					
Mean of Scr.RNAi	7.924				
Mean of Ankrd24 RNAi-1	6.004				
Difference between means	1.920				
SE of difference	0.1701				
95% CI of difference	1.586 to 2.254				
Data summary					
Number of columns (Column Factor)	2				
Number of rows (Row Factor)	11				
Number of values	1100				

Reviewer 3 Figure 2. Sholl analyses of Ankrd24 loss-of-function analyses (see Figure 5H) here shown as line representation of the data together with the statistical analysis of the two lines.
Note that, also in the line type of data presentation, the identified Ankrd24 loss-of-function phenotype in dendritic arbor formation (**Figure 5; Figure S3 of revised manuscript**) is fully valid and statistically highly significant.

- With every deletion being unable to rescue dendrite growth one wonders if the authors are just producing proteins that are not folding properly or are being degraded. The data would be much more convincing if:
1) images of GFP are shown indicating similar levels to WT ankrd24-GFP, 2) they show that a point or multiple point mutant (rather than a deletion) also doesn't rescue and 3) they show one type of deletion that does rescue.

The reviewer is referring to the rescue analyses with Ankrd24 mutants shown in Figure 6. The expression of the Ankrd24 mutant proteins tested in functional analyses is indeed a point worthwhile to consider carefully and we of course did that. GFP images were already included for **Figure 4**, **5** and **S3**. The mutant analysis in Figure 6 the reviewer criticizes contains no images due to space limitations. However, we would kindly like to refer the reviewer to the accompanying Figure S4, which shows the GFP images of all mutants tested in Figure 6. It can be seen that the expression levels are similar. Rescues by reexpression under the viral CMV promotor of pRNAT of course are clearly above the endogenous level, i.e. should in all cases be high enough for putative rescue of the knock-down of the endogenous Ankrd24 of the neurons, if the reexpressed protein was functional, as demonstrated for the WT protein.
It is of course also worthwhile to carefully think about dysfunctionality due to general misfolding instead of a specific dysfunction due to the domain taken out of the equation by deletion or mutation. **This, however, does not apply to our study, as we included multiple mechanistic work and different experimental readouts for all mutants. We made this point clearer in the revised manuscript.**
In detail:
Even the protein lacking aa2-285 ($Ankrd24^{cc}$) is both well-expressed (please see **Figure S4C** for Western blotting data) and is fully self-association-competent (**Figure S4C**).
For the Ankrd24 mutant that lacks the entire coiled coil region ($Ankrd24^{1-285}$) we have demonstrated that it is readily expressible, stable and binds to both the plasma membrane and syndapin (please see **Figure 1C,H** and **Figure 2K**).
In the case of the amphipathic helix deletion, only 8 amino acids (2-9) are missing and thus only the far N terminus of Ankrd24 is minimally shortened. The introduced minimal deletion does neither affect the putative N terminal cap structure composed of two alpha helices (aa21-41) nor does it affect the ankyrin repeats of the ankyrin repeat array, the first α-helix of which starts at aa56. **We highlighted this in the revised manuscript. During our revision work, we furthermore generated experimental evidence that demonstrated that also this Ankrd24 mutant is not only readily expressed (Figure 2G, Figure S4D,L) but also still is functional in its non-N-Ank properties, such as in self-association (Figure S4D) and syndapin I binding (Figure S4F).**
The ΔKRAP mutant merely lacks 14 amino acids (aa273-286). Also in this case, we made sure that these amino acids are located in a flexible, structurally not ordered part of Ankrd24 (please see **Figure 2B,D**). General protein misfolding that could potentially affect the more N terminal and structurally rigid ankyrin repeats (aa56 to 213) or the C terminal coiled coil region (aa283-1041) thus is highly unlikely. In line, $Ankrd24^{\Delta KRAP}$-GFP was readily expressible, stable and intact (please see **Figure 1C,E**). **We in addition demonstrated in our revision work that $Ankrd24^{\Delta KRAP}$-GFP is still self-association-competent (Figure S4B).**
The evidence provided already in the previous manuscript and the expanded analyses conducted during our revision work thus rule out that putative general protein misfolding may be the reason for the observed dysfunctionality of the Ankrd24 mutants tested in our functional work (**Figure 6**). **Instead, all mutants were observed to be specifically deficient for the function affected by the respective (and in part minimal) deletions.**

- There still looks like about 50% interaction with the delta-KRAP Ankrd24 in the first blot in Fig. 1C (and Fig. S1A), but no co-IP in the second blot. Please explain such an obvious discrepancy.

This obviously is a misunderstanding of the CoIP experiments shown in Figure 1 **(former Figure 1C; now Figure 1E in the revised manuscript).** We would kindly like to refer the reviewer to the labeling of the different blots shown in **Figure 1E and Figure S1D (former Figure S1A)** that can be found at right side of each of the blots.
The left panel of **Figure 1E** shows the input, i.e. the expression of GFP, Ankrd24-GFP (WT) and the Ankrd24$^{\Delta KRAP}$-GFP mutant (upper blot of left panel of **Figure 1E**) and the input of Flag-syndapin I (lower blot of left panel of **Figure 1E**) to show where each protein tested runs and that they are intact. Thus, it is correct that all three GFP proteins are detected on the blot on the left. The same is true for **Figure S1D**, which shows the supernatants of the CoIPs. Please note that (an excess of) GFP proteins is still readily detectable and remained intact after incubation, i.e. all proteins have successfully been offered for IP (Flag-syndapin I) and for CoIP (GFP fusion proteins).
The right blots of **Figure 1E** then show the CoIP results. All samples received the same of amount of antibody (anti-Flag or non-immune IgG), as demonstrated by the general IgG heavy chain detection (lower blot of right panel of **Figure 1E**). Flag-syndapin I was specifically IPed by anti-Flag antibodies but not by the non-immune IgGs (middle blot of right panel of **Figure 1E**). Ankrd24-GFP was specifically CoIPed (i.e. is only visible in the lane with samples containing syndapin I IP but not in the lane with non-immune IgG control). In contrast, neither Ankrd24$^{\Delta KRAP}$-GFP nor GFP alone showed any signals in either syndapin I CoIP samples or control samples (upper blot of right panel of **Figure 1E**).
Thus, the CoIP of Ankrd24 with syndapin I is specific and very clear. In contrast, Ankrd24$^{\Delta KRAP}$-GFP fails to associate with syndapin I. There is no discrepancy.

- Fig. S1B-D are the same cells in Fig. 1E-G, just showing separate channels and a line scan. Fig. 1E-G should be removed and replaced by Fig. S1B-D. Also, the full length Ankrd24 looks a lot less specific than the 1-285 fragment regarding recruitment to Mito.

The purpose of **Figure S1B-D (now Figure S1E-G in the revised manuscript)** indeed was to show an even more detailed colocalization analyses (additional line scans) and also all the single channels corresponding to the main figure panels, which showed the merges in high magnifications (**Figure 1G-I of the revised manuscript**).
We were unable to integrate **Figure S1E-G** into the main figure, as space did not permit this after we added the revision work the reviewers requested to **Figure 1**. **The more detailed analysis of the colocalizations by additional line scan analyses thus had to stay in the Supplementary Information (please see Figure S1 of the revised manuscript).**

- The authors indicate in Fig. 9G that the liposomes resulting from the Ankrd24(1-285)/syndapin I(SH3 mutant) is statistically indistinguishable from control liposomes. The images (9A and 9F) and graph provided (9G, first and last bars) are not consistent with that statement. There seems to be many smaller liposomes in 9F(arrows) compared to 9A and the means also seem quite different in the bar graph.

The quantitative data shown in **Figure 9G** is correctly described. The experimental condition Ankrd24$^{1\text{-}285}$ + SdpI^{P434L} is statistically indistinguishable from control liposomes (**Figure 9G**).

We thank the reviewer for his/her careful evaluation of our example images. A quantitative evaluation of the individual liposomes shown in the example image **Figure 9F** indeed showed some discrepancy to the mean of the quantitative analysis. The chosen example image thus indeed was not a good representative image. **We identified an alternative image that shows very exact quantitative matching of the quantitative data and replaced the example image in Figure 9F with this better suited image to illustrate the results of the quantitative analyses covering hundreds of liposomes.**

- The construct Ankrd24(10-285) is a good negative control and the data in Fig. 2K-M is convincing. Why wasn't Ankrd24 (10-285) used as a negative control in the differential centrifugation or expression in HEK cells?

As the reviewer suggested, we conducted additional experiments using an amphipathic helix deletion mutant of Ankrd24 in the fractionation experiments using HEK293 cells. **The new experimentation shows an absence of Ankrd24$^{10\text{-}1041}$-GFP in the membrane fractions P2 and P2', while the coexpressed plasma membrane marker mCherryF was strongly detected in these membrane fractions (please see newly inserted Figure 2G of the revised manuscript).**

- Line 246-48 - It is unclear why the shRNA resistant form of Ankrd24 (Ankrd24*) is only included in a supplemental figure and not a regular figure. This is an important piece of data.

It indeed is an important set of data to vigorously demonstrate the specificity of RNAi. However, as corresponding RNAi and rescue data is shown only one figure later, as part of **Figure 6**, in which the Ankrd24 mutants are analyzed together with the WT rescue, and as we cannot exceed the maximal number of main figures, we had to leave this additional data set in the Supplementary Information.
The identified Ankrd24 loss-of-function phenotype shown in **Figure 5** is well-underscored by the second data set obtained by the independent RNAi tool presented in **Figure S3** and its rescue (**Figure S3**). The rescue for the first RNAi tool is then shown as part of the mutational analyses in **Figure 6**.

- The authors use "clearly" 17 times throughout the manuscript. This modifier should be used sparingly as it is up to the reader to determine whether the authors showed something clearly. The authors also refer to the fact that when the Ankrd24(1-285) was analyzed it was "irritating" that the proposed amphipathic helix was lacking. Although I am sure it was, it does not seem to be appropriate to include this feeling in a scientific manuscript.

We thank the reviewer for his/her comments. **We reworded the sentence reporting the irritating inconsistency of the Alphafold3 predictions.**
We furthermore deleted all "clearly"s in the data descriptions in the revised manuscript.

- Regarding Fig. 1D, does the full length Ankrd24 bind syndapin I directly or just the 1-285 fragment?

Direct binding analyses, as those shown in the former **Figure 1D (now Figure 1F in the revised manuscript**), require the purification of both binding partners from a heterologous cell system. In case of syndapin I and Ankrd24, bacteria are a suitable system. Yet, while syndapin I and

Ankrd24$^{1-285}$ can be purified from this heterologous cell system, Ankrd24 unfortunately cannot be purified (most likely due to its large size and furthermore due to its extended coiled coil domain). Thus, formally a direct interaction can only be proven with Ankrd24$^{1-285}$ instead of also with the full-length. Our experiments clearly demonstrated that Ankrd24$^{1-285}$ is sufficient for direct interaction (**Figure 1F**).
Several other experiments included in our work clearly demonstrated the Ankrd24/syndapin I interaction using full-length proteins. Please see coprecipitations in **Figure 1B,C**, the coimmunoprecipitations in **Figure 1E** and the intracellular reconstitution of the interaction at mitochondrial surfaces of intact cells (**Figure 1G**) for demonstrations of full-length protein interactions.

- Ankrd24 is never defined as ankyrin repeat domain-containing protein 24 in the manuscript.

We thank the reviewer for his/her note. **We spelled out Ankrd24 in the revised manuscript.**

- Line 159-67 - the panel letters of Fig. 2F-M are mislabeled in the text.

We sincerely apologize for this embarrassing mistake and very much thank the reviewer for noticing this mistake, which probably occurred upon insertion of scheme **Figure 2E. We corrected this and made sure that all figure panels are cited in the revised manuscript.**

- Line 206 - Please add the age of the rat(s) from which the brain lysate was prepared.

We added this information to the revised manuscript (the lysates were from ~15 w old rats)**.**

May 11, 2026

Re: JCB manuscript #202509074R

Michael Kessels
Jena University Hospital - Friedrich Schiller University Jena

Dear Dr. Kessels,

Thank you for submitting your revised manuscript entitled "The N-Ank protein Ankrd24 shapes neurons and couples to syndapin and the F-actin promoter Cobl-like" to Journal of Cell Biology. The manuscript has been assessed by the original reviewers, whose reports are appended below.

You will see that, while the reviewers appreciate the work that has gone into the revisions, they still voice substantial critiques, such as those regarding adequate replicates, and note unresolved issues, particularly from reviewers #2 and 3.

JCB policy is that papers are considered through only one major revision cycle. Given the significant remaining reviewer concerns, and the overall low reviewer enthusiasm, we unfortunately cannot offer publication of the manuscript.

If you would like to transfer your reviewer comments to another journal for consideration elsewhere, please contact the journal office at cellbio@rockefeller.edu and we would be happy to arrange the transfer on your behalf.

We appreciate the effort that has gone into the revisions and regret that the outcome is not more positive.

Sincerely,

Tobias Meyer
Monitoring Editor
Journal of Cell Biology

Gabriele Stephan
Scientific Editor
Journal of Cell Biology

Reviewer #1:

In this revision, Jain et al. have mostly addressed my concerns. However, I cannot help but argue against a few statements:
1. In vitro = in the dish (also cells). Cells in a dish are never considered in vivo.
2. In vivo = in the animal.
While I understand the editors believe loss and gain of function experiments are outside the scope of the revision, I disagree with the authors' contention that it is "virtually impossible to experimentally address the molecular details of Ankrd24 functions in the whole brain." The GOF or LOF experiments can be done using AAV or other approaches. In future studies I encourage the authors to investigate approaches to demonstrate their interesting biological results in the context of the brain, in vivo. I only cited in vivo work as the in vitro work represents a limitation of the present study and in vivo work would only extend the significance of the work.

Second, preparing cultures from multiple embryos and mixing 6 embryos is a single experiment. N=1. Each cell analyzed on a coverslip is a technical replicate - you have no idea whether the neurons analyzed come from the same embryo or not, so the rigorous approach is to consider them technical replicates. The biological replicate is the culture preparation itself. If neurons from each embryo were plated separately, each embryo could be considered a biological replicate. Counting multiple measurements from the same biological unit (e.g. one cell, one culture dish, one animal) as independent experiments artificially inflates the statistical power. A technical replicate would be running the same samples on a different gel, or plating the same cohort of cells on different slides and then repeating the experiment. A biological replicate is analyzing different samples entirely, and different embryos. It is most appropriate to analyze many cells on a given slide, average results for that slide, then do a statistical comparison between the averages from biologically independent experiments (i.e. different cell culture experiments). My concerns match those of Reviewer #3.
In any case, it is a moot point because the authors have now repeated individual assays to satisfy my concern.

Reviewer #2:

Jain et al., revised, under consideration at JCB.
Several of the concerns that I had previously raised have been addressed, resulting in an overall stronger manuscript. However, one major and one minor concern remain, both of which can be addressed by revisions of the text:

In point 3 of my previous review, I had suggested including a discussion that compares the results of the current manuscript with the previously published results regarding ankycorbin (Izadi et al., 2023, JCB). My comment was:

"3. Previous work on Ankycorbin and Ankrd24. Given the similarity of the findings for Ankrd24 in the current manuscript and for Ankycorbin in the recent study from the same group (Izadi et al., 2023, JCB), it was surprising that the parallel findings were not thoroughly discussed in the discussion section. What are their functional similarities/differences, is there functional redundancy, how do they differ in sequence/domain structure, what is known about their expression patterns during neurodevelopment? ..."

I do not see how this point was addressed in the revised manuscript, and no response was provided in the response letter. The working models for Ankrd24 in the current manuscript (Fig. 10J) and the graphical abstract/model for ankycorbin in Izadi et al., 2023 (Fig. 10) are strikingly similar so that a comparative discussion of the mechanisms by which these two proteins function should be included. Both proteins have an N-terminal amphipathic helix, an ankyrin repeat motif, a KRAP motif with which they interact with syndapins, for coupling to Cobll1, for F-actin formation, and they promote neuronal branching

A minor comment is that clarity of the text could be further improved - some sections would benefit from a more concise writing style. Examples are the long sub-heading on P11 of the merged pdf, sentences 4 and 5 in the abstract, or the third sentence of the discussion. The title could also be further improved - I find that "F-actin promoter" invokes transcription. Overall, some of the new edits require checking for language/grammar inconsistencies.

Reviewer #3:

Re-Review of Jain et al.

Although the authors have included more data and fixed several concerns from this reviewer there are still significant deficiencies in the manuscript that are outlined below.

- Are the authors really trying to argue that combining a litter of prenatal embryos accounts for 28 individuals and try to argue that three experiments result in unnecessary killngs? Why not just do one experiment then? The neurons in the embryos are combined, making one experiment. If the authors wanted to separate every embryo and compare it to its siblings this might be considered multiple N's, but it is still from one mother, so all those data are not independent. Also, it doesn't matter that their data were highly significantly different after two experiments, a third experiment may have changed that significance. If they haven't already, the authors should read Superplots (PMID: 32346721), also published in JCB. They confuse technical replicates and biological replicates. Every cell or every vesicle are not biological replicates. Reviewer 1 also brought up this point as well. If all their previous work were done as two experiments (i.e. cultures), I would question that work as well, whether it has been published. The data provided in Reviewer 1/Figure 1 would be a more appropriate way to show biological replicates. This should be repeated for the entire paper.

- The additional data provided in Fig. 4L is not convincing. One frame showing a concentration of GFP-Ankrd24 does not necessarily indicate a concentration of Ankrd24 regularly at nascent branch points (as outlined in their model in Fig. 10J). The authors would have to ratio it against RFP (not just LifeAct-RFP) to control for an increase in volume and quantify multiple nascent dendritic branches. Also, the authors refer to it as the protrusions "...forming a dendrite branch" in the figure legend. The structure that they documented is a filopodium that may or may not form a branch. Imaging would have to be performed over much longer intervals to prove it becomes a branch. In the movie provided it is obvious that the protrusions are filopodia, several of which are very transient (<15 min. lifetime). Renaming them filopodia, which might give rise to a nascent dendritic branch, would be appropriate.

- Regarding the number of dendritic segments, I agree with the authors that they should not be connected by a line and that comparisons can be made among primary, secondary, tertiary, etc. dendritic segments (that was my fault). However, regarding my statement of the authors' Scholl analysis, I was not indicating that their data were meaningless. Rather it was their extensive statistical comparisons between the concentric circles, rather than their different manipulations (OE, KD, etc), was meaningless. The number of concentric circles is arbitrary; they can be separated by however many microns the researcher decides.

Moreover, the Scholl paper is a 1953 paper (not 1963 as they indicate in their rebuttal) and Sholl only showed dots representing the number of crossings of each concentric Sholl line. Sholl connected the dots with a regression line after he log transformed the data, showing it was a straight line for basal dendrites (Scholl, Fig. 13), but never compared differences between individual Scholl lines as the authors do in this paper. Put another way, the authors are trying to compare manipulations (OE, KD, mutant) and they indicate that their "N" represents neuron numbers. Thus, each manipulation in Fig. 4J, 5H, 6E, 10D should be represented by a single mean line with SEM. The authors should then do statistics comparing each line to one another (not each bar respresenting a Sholl circle). They could then conclude that one line (manipulation - OE, shRNA, mutant, etc.) is significantly different from another line. The reader could then more easily judge whether an overexpression, knockdown or mutant is changing the dendritic complexity. Comparisons between each individual Sholl line (a bar in their figures) is irrelevant. The authors indicate the lines would overlap for multiple conditions. Isn't that the point? Only where the lines don't overlap is each manipulation likely to be different. The graph in Reviewer 3, Figure 2 (in their rebuttal) is so much easier to extract information than the bar graphs in the paper and more accurate as well.

- Regarding labeling only with MAP2 and counting axons as dendrites the authors indicated they "...carefully reevaluated each individual tracing done in the study by and experienced postdoc in the lab and made required changes." This is not scientifically valid. It wouldn't matter how experienced the person quantifying the dendrites is, MAP2 is not only in dendrites at DIV6. They need to use another label to discount MAP2 in axons at this age.

- Is Fig. 3K really highly significantly different? Was that just a cut/paste error?

Our point-to-point responses to the Reviewer's comments

Reviewer #1:
In this revision, Jain et al. have mostly addressed my concerns. However, I cannot help but argue against a few statements:
1. In vitro = in the dish (also cells). Cells in a dish are never considered in vivo.
2. In vivo = in the animal.

While we already had avoided the term in vivo carefully in all of our descriptions of observations and data and also did not use it in any interpretations **we have now also reworded the only use of this term in the introduction of the rationale behind our additional coimmunoprecipitation efforts (Results section)**. The revised sentence now reads "*In order to further address the relevance of the identified syndapin I interaction with Ankrd24, we next conducted coimmunoprecipitations.*"

While I understand the editors believe loss and gain of function experiments are outside the scope of the revision, I disagree with the authors' contention that it is "virtually impossible to experimentally address the molecular details of Ankrd24 functions in the whole brain." The GOF or LOF experiments can be done using AAV or other approaches. In future studies I encourage the authors to investigate approaches to demonstrate their interesting biological results in the context of the brain, in vivo. I only cited in vivo work as the in vitro work represents a limitation of the present study and in vivo work would only extend the significance of the work.

Generating Ankrd24 KO mice and analyzing them for changes in brain functions would certainly represent a significant future advance in science. We appreciate the encouragement of the reviewer to embark on such efforts.

Second, preparing cultures from multiple embryos and mixing 6 embryos is a single experiment. N=1. Each cell analyzed on a coverslip is a technical replicate - you have no idea whether the neurons analyzed come from the same embryo or not, so the rigorous approach is to consider them technical replicates. The biological replicate is the culture preparation itself. If neurons from each embryo were plated separately, each embryo could be considered a biological replicate. Counting multiple measurements from the same biological unit (e.g. one cell, one culture dish, one animal) as independent experiments artificially inflates the statistical power. A technical replicate would be running the same samples on a different gel, or plating the same cohort of cells on different slides and then repeating the experiment. A biological replicate is analyzing different samples entirely, and different embryos. It is most appropriate to analyze many cells on a given slide, average results for that slide, then do a statistical comparison between the averages from biologically independent experiments (i.e. different cell culture experiments). My concerns match those of Reviewer #3. In any case, it is a moot point because the authors have now repeated individual assays to satisfy my concern.

We are happy to see that increasing the numbers of independent cell source preparations for the dissociated primary hippocampal cultures we later used for our cellular investigations to 3-5 during our revision work satisfied the reviewer.

In addition, we would like to refer the reviewer to the new representations of all dendritic branch point, all dendritic terminal point and all dendritic length analyses in the newly revised manuscript. The revised

figure panels now transparently show the consistence of the distribution and variance of the individual cells in each (color-coded) assay (please see **revised Figures 4, 5, 6, 7 and 10** as well as the **revised Supplementary Figures S2, S3 and S5**).
We also expanded the description that and how we evaluate the individual data, the consistence between different assays and the variances at the different levels in Material and Methods of the revised manuscript.

In addition, we added "SuperPlot" representations referred to by reviewer 3 (Lord et al., 2020). Also this change in data representation we did for the quantitative dendritic branch point, the dendritic terminal point and the dendritic length analyses for all of our main Ankrd24 phenotypes:

- gain-of-function
- loss-of-function as well as
- rescue experiments with WT Ankrd24*-GFP vs. the different Ankrd24-GFP* mutants

Please see **revised Figures S2F-H, S3F-H and S5F-H**. Besides the color-coded dots representing individual measurements (see also above), the newly added SuperPlots also show mean-of-the-mean calculations (black line and error bars) representing the low variance from culture to culture (marked by big differentially colored dots).
Also in these calculations of the data, the identified Ankrd24 gain-of-function phenotypes in dendritic branching points, dendritic terminal points and total dendritic tree length as well as the identified Ankrd24 loss-of-function phenotypes are fully valid and also statistically significant.

Reviewer #2:
Jain et al., revised, under consideration at JCB.
Several of the concerns that I had previously raised have been addressed, resulting in an overall stronger manuscript. However, one major and one minor concern remain, both of which can be addressed by revisions of the text:

In point 3 of my previous review, I had suggested including a discussion that compares the results of the current manuscript with the previously published results regarding ankycorbin (Izadi et al., 2023, JCB). My comment was:
"3. Previous work on Ankycorbin and Ankrd24. Given the similarity of the findings for Ankrd24 in the current manuscript and for Ankycorbin in the recent study from the same group (Izadi et al., 2023, JCB), it was surprising that the parallel findings were not thoroughly discussed in the discussion section. What are their functional similarities/differences, is there functional redundancy, how do they differ in sequence/domain structure, what is known about their expression patterns during neurodevelopment? ..."
I do not see how this point was addressed in the revised manuscript, and no response was provided in the response letter. The working models for Ankrd24 in the current manuscript (Fig. 10J) and the graphical abstract/model for ankycorbin in Izadi et al., 2023 (Fig. 10) are strikingly similar so that a comparative discussion of the mechanisms by which these two proteins function should be included. Both proteins have an N-terminal amphipathic helix, an ankyrin repeat motif, a KRAP motif with which they interact with syndapins, for coupling to Cobll1, for F-actin formation, and they promote neuronal branching.

We apologize for not having specifically responded to this point of the reviewer. The different aspects of the Ankrd24 and ankycorbin comparison being scattered over the introduction (N-Ank superfamily; self-association; syndapin I interaction; ankycorbin, UACA, Ankrd24, Ankrd35 subfamily), the results section (N-Ank nature (Fig. 1); KRAP motif presence (Fig. 1); syndapin I interaction (Fig. 4)) and the discussion (N-Ank; SH3/KRAP interaction interface) might alone indeed not be clear enough, albeit the respective reference for comparisons with ankycorbin (Izadi et al., 2023 *JCB* and Wolf et al., 2019 *Nat Cell Biol* were cited 11times and 22 times, respectively. **We therefore gave the remaining topics the reviewer mentioned – phylogenetic similarity, expression patterns during neurodevelopment, putative functional redundancy (page 19,20 in the tracked revised manuscript) and an apparently similar role of ankycorbin in dendritic arbor formation (page 22 in the tracked revised manuscript) - additional room in the discussion of the newly revised manuscript.**
We also mentioned that it is to our knowledge currently unknown whether ankycorbin or any other N-Ank superfamily protein forms similar complexes with cellular machinery promoting local F-actin formation, such as Cobl-like (page 23 in the tracked revised manuscript). Also this sentence added to the discussion of the revised manuscript now contributes to the multiple comparisons of ankycorbin and Ankrd24 throughout the revised manuscript.
We hope that the reviewer will agree with us that, in the revised manuscript, our work on Ankrd24 now also contains sufficient information about the other, in part related N-Ank superfamily members, in particular ankycorbin.

A minor comment is that clarity of the text could be further improved - some sections would benefit from a more concise writing style. Examples are the long sub-heading on P11 of the merged pdf, sentences 4 and 5 in the abstract, or the third sentence of the discussion. The title could also be further improved - I find that "F-actin promoter" invokes transcription. Overall, some of the new edits require checking for language/grammar inconsistencies.

We thank the reviewer for his/her careful evaluation of the revised manuscript text. **We hope that the reviewer will be content with the changes made to improve the consistency and readability of the newly revised manuscript.**

The long subheading containing the listing of the different insights obtained in the functional analyses using Ankrd24 deficiency in combination with different Ankrd24 mutants in rescue experiments has been shortened by omitting specific information. Both sentence 4 and sentence 5 in the abstract have been split into two sentences in the newly revised manuscript. Also the third sentence of the discussion was split into two.

The title of the manuscript has been discussed again. It had been changed due to reviewer demands in the previous revision. The editors did not suggest to change it again. We are aware of the dual use of the word promoter. Yet, we hope that the reviewer agrees that the term “F-actin promoter” should be sufficiently distinct from the process of transcription of DNA. Unfortunately, we cannot name it “F-actin-promoting protein Cobl-like”, which would be scientifically correct and preferred by all authors but would unfortunately violate JCB’s title length restrictions. The currently available data for Cobl-like also does not justify the use of the term “actin nucleator” for Cobl-like as an alternative. Cobl-like promotes the formation of actin filaments in a complex process that could not be reconstituted for technical reasons and therefore is mechanistically unproven. Also the term NPF (actin nucleation promoting factor) is not appropriate because, in the actin field, this term is referring to promoters of Arp2/3 complex activity (leading to actin nucleation by the Arp2/3 complex) and Cobl-like has no Arp2/3 complex docking motif.

Reviewer #3:
Although the authors have included more data and fixed several concerns from this reviewer there are still significant deficiencies in the manuscript that are outlined below.

- Are the authors really trying to argue that combining a litter of prenatal embryos accounts for 28 individuals and try to argue that three experiments result in unnecessary killngs? Why not just do one experiment then? The neurons in the embryos are combined, making one experiment. If the authors wanted to separate every embryo and compare it to its siblings this might be considered multiple N's, but it is still from one mother, so all those data are not independent.
Also, it doesn't matter that their data were highly significantly different after two experiments, a third experiment may have changed that significance. If they haven't already, the authors should read Superplots (PMID: 32346721), also published in JCB. They confuse technical replicates and biological replicates. Every cell or every vesicle are not biological replicates. Reviewer 1 also brought up this point as well. If all their previous work were done as two experiments (i.e. cultures), I would question that work as well, whether it has been published. The data provided in Reviewer 1/Figure 1 would be a more appropriate way to show biological replicates. This should be repeated for the entire paper.

We would kindly like to refer the reviewer to the fact that we have increased the numbers of independent cell source preparations for the dissociated primary hippocampal cultures we used for our cellular manipulations to 3-5 during our revision work.
The different cohorts of animals used for each culture of primary cells later transfected with different plasmids were of course derived from different mothers. As reported in the Material and Method section, not only the E18 animals but also the pregnant mother is killed. The cohorts of littermates used as a source for primary cells are thus from independent mothers. The animal numbers for the analyses added during revision were reported correctly in our first reviewer response.

In addition, we would like to thank the reviewer for pointing out the Lord et al., 2020 paper on how to color dots from individual assays in larger cellular examinations to visualize the data distribution of individual cells in different assays used. We have replaced all quantitative panels showing dendritic branch point, dendritic terminal point and dendritic length analyses with color-coded ones. **The revised figure panels now transparently show the consistence of the data distribution of individual cells in each (color-coded) assay and the high variance of the individual cells in these examinations of dissociated neurons (please see revised Figures 4, 5, 6, 7, 10, S2, S3 and S5).**

We also expanded the description that and how we evaluate the individual data, the consistence between different assays and the variances at the different levels in Material and Methods of the revised manuscript.

In addition, we added the suggested "SuperPlot" representations. Also this change in the data representation we did for the quantitative dendritic branch point, the dendritic terminal point and the dendritic length analyses for all of our main Ankrd24 phenotypes:

- **gain-of-function**
- **loss-of-function as well as**
- **rescue experiments with WT Ankrd24*-GFP vs. the different Ankrd24-GFP* mutants**

Please see revised Figures S2F-H, S3F-H and S5F-H. Besides the color-coded dots representing individual measurements (see also above), the newly added SuperPlots also show mean-of-the-mean calculations (black line and error bars) representing the low variance from culture to culture (marked by big differentially colored dots).

Also in these calculations of the data, the identified Ankrd24 gain-of-function phenotypes in dendritic branching points, dendritic terminal points and total dendritic tree length as well as the identified Ankrd24 loss-of-function phenotypes are fully valid and also statistically significant.

- The additional data provided in Fig. 4L is not convincing. One frame showing a concentration of GFP-Ankrd24 does not necessarily indicate a concentration of Ankrd24 regularly at nascent branch points (as outlined in their model in Fig. 10J). The authors would have to ratio it against RFP (not just LifeAct-RFP) to control for an increase in volume and quantify multiple nascent dendritic branches. Also, the authors refer to it as the protrusions "...forming a dendrite branch" in the figure legend. The structure that they documented is a filopodium that may or may not form a branch. Imaging would have to be performed over much longer intervals to prove it becomes a branch. In the movie provided it is obvious that the protrusions are filopodia, several of which are very transient (<15 min. lifetime). Renaming them filopodia, which might give rise to a nascent dendritic branch, would be appropriate.

We thank the reviewer for his/her comment referring to the newly added experimentation going beyond the revision plan. **We have changed the text of the revised manuscript in the results, discussion and legends.** As you will see in the tracked revised manuscript, we carefully avoided any wording that could be viewed as an overinterpretation and now either merely report that both Ankrd24-GFP and F-actin tracked by LifeAct are present at nascent dendritic protrusion induction sites in 3D-time lapse analyses (Fig. 4L).
As the ultrastructure of the observed dendritic protrusions critically relying on Ankrd24 is unknown (filopodia would per definition be made up by elongated, bundled F-actin filaments with their barbed ends all facing the tip), we agree with the reviewer that it currently seems scientifically more precise to merely describe what one directly sees in the live analyses: protrusions forming from dendrites. **We hope that the reviewer will agree with us that the newly revised manuscript now describes this in a better way.**
The model simply brings together the membrane topologies at these sites with the molecular properties of Ankrd24 we identified.

- Regarding the number of dendritic segments, I agree with the authors that they should not be connected by a line and that comparisons can be made among primary, secondary, tertiary, etc. dendritic segments (that was my fault). However, regarding my statement of the authors' Scholl analysis, I was not indicating that their data were meaningless. Rather it was their extensive statistical comparisons between the concentric circles, rather than their different manipulations (OE, KD, etc), was meaningless. The number of concentric circles is arbitrary; they can be separated by however many microns the researcher decides. Moreover, the Scholl paper is a 1953 paper (not 1963 as they indicate in their rebuttal) and Sholl only showed dots representing the number of crossings of each concentric Sholl line. Sholl connected the dots with a regression line after he log transformed the data, showing it was a straight line for basal dendrites (Scholl, Fig. 13), but never compared differences between individual Scholl lines as the authors do in this paper. Put another way, the authors are trying to compare manipulations (OE, KD, mutant) and they indicate that their "N" represents neuron numbers. Thus, each manipulation in Fig. 4J, 5H, 6E, 10D should be represented by a single mean line with SEM. The authors should then do statistics comparing each line to one another (not each bar respresenting a Sholl circle). They could then conclude that one line (manipulation - OE, shRNA, mutant, etc.) is significantly different from another line. The reader could then more easily judge whether an overexpression, knockdown or mutant is changing the dendritic complexity. Comparisons between each individual Sholl line (a bar in their figures) is irrelevant. The authors indicate the lines would overlap for multiple conditions. Isn't that the point? Only where the lines

don't overlap is each manipulation likely to be different. The graph in Reviewer 3, Figure 2 (in their rebuttal) is so much easier to extract information than the bar graphs in the paper and more accurate as well.

We **have added line representations of the Sholl data to the revised manuscript (please see Figure 4J, Figure 5H, Figure 6E, Figure 10I, Figure S2E, Figure S3E,O and Figure S5E of the revised manuscript). We also added the respective statistical significance information** to the revised figures and revised legends, respectively. For Figure 6E, Figure 10I and Figure S5E (multiple lines), the data of the multiple comparisons are reported in detail in the source data file due to space restrictions.

- Regarding labeling only with MAP2 and counting axons as dendrites the authors indicated they "...carefully reevaluated each individual tracing done in the study by and experienced postdoc in the lab and made required changes." This is not scientifically valid. It wouldn't matter how experienced the person quantifying the dendrites is, MAP2 is not only in dendrites at DIV6. They need to use another label to discount MAP2 in axons at this age.

We are not quite sure whether this simply is a misunderstanding. We solely mentioned the dendritic marker MAP2 as basis for our analyses to make sure that readers understand that the quantitative analyses were not done using the GFP channel but by tracking of an endogenous, dendritic marker, that was independent from any transfections. The axon of the fully polarized cells (the time of analysis (DIV6) is ~48 h prior to the start of synapse and functional network formation in our cultures) of course has always been excluded from the evaluations of the dendritic arbor. **We have therefore improved our description that and how the axon was excluded in the revised manuscript**. The axon has been excluded by a combination of its low MAP2 abundance and the usual morphological parameters (the list and a respective reference as been added to the Material and Method section of the newly revised manuscript). **Additionally we would like to refer the reviewer to the fact that we explicitly did double-check the axon exclusion by the anti-tau counterstains the reviewer requested.**

- **Following the suggestion of the editors, we now included the tau-counterstained gain-of-function data set (previously merely shown it as Reviewer Figure) into the Supplements (please see Figure S2A-E of the revised manuscript).**
- Beyond this editorial suggestion, **the Ankrd24 loss-of-function phenotype in dendritic arbor formation we identified (Fig. 5) is now complemented with a corresponding anti-MAP2/anti-tau colabeled data set in the revised version of the manuscript (please see Figure S3A-E of the revised manuscript)**. It of course also shows the Ankrd24 phenotype in dendritic arbor formation.
- Fusing the former Figure S1 and S2 to the new Figure S1, we even made space for being able to **also report the anti-tau counterstained data subset of the Ankrd24 loss-of-function/rescue vs. rescue with mutants data (corresponding to Fig. 6) in the Supplements.** Also in these data sets, the data for all rescue attempts with the different mutants still resemble the levels of Ankrd24 RNAi (**please see Figure S5A-E of the revised manuscript**).

The newly added anti-tau counterstain data nicely confirm that the combinations of using morphological criteria, the low abundance of the dendritic marker MAP2 and/or presence of the axon marker tau (as in the new sets of experimentation) for the axon exclusions are yielding valid and consistent data.

- Is Fig. 3K really highly significantly different? Was that just a cut/paste error?

We thank the reviewer for his/her comment. We double-checked this again. The data reflected by the newly added Fig. 3K (a quantitative analysis of EM images analyzed for the anti-GFP immunogold labeling

density of freeze-fractured large vs. small liposomes incubated with purified GFP as control) **is displayed correctly and also described correctly in the text.**

Furthermore, we would like to refer the reviewer to the numerical source data for Fig. 3K. It allows all readers to freely interact with our data and to double-check e.g. data distribution and/or statistical significances.

August 7, 2026

RE: JCB Manuscript #202509074RR-A

Michael Kessels
Jena University Hospital - Friedrich Schiller University Jena

Dear Dr. Kessels:

Thank you for submitting your revised manuscript entitled "The N-Ank protein Ankrd24 shapes neurons and couples to syndapin and the F-actin promoter Cobl-like". We have editorially assessed your revised manuscript, and we would be happy to publish your paper in JCB pending final revisions necessary to meet our formatting guidelines (see details below).

To avoid unnecessary delays in the acceptance and publication of your paper, please read the following information carefully.

A. MANUSCRIPT ORGANIZATION AND FORMATTING:

Full guidelines are available on our Instructions for Authors page, http://jcb.rupress.org/submission-guidelines#revised.
Submission of a paper that does not conform to JCB guidelines will delay the acceptance of your manuscript.

1) Text limits: Character count for Articles is < 40,000, not including spaces. Count includes abstract, introduction, results, discussion, and acknowledgments. Count does not include title page, figure legends, materials and methods, references, tables, or supplemental legends.

2) Figures limits: Articles may have up to 10 main text figures.

3) Figure formatting: Scale bars must be present on all microscopy images, including inset magnifications. Molecular weight or nucleic acid size markers must be included on all gel electrophoresis. Aspect ratios of images may not be altered.

*In order to accommodate readers with red-green color blindness, we encourage you to change the red/green color scheme in some of your figures.

4) Statistical analysis: Error bars on graphic representations of numerical data must be clearly described in the figure legend. The number of independent data points (n) represented in a graph must be indicated in the legend. Statistical methods should be explained in full in the materials and methods. For figures presenting pooled data the statistical measure should be defined in the figure legends. Please also be sure to indicate the statistical tests used in each of your experiments (either in the figure legend itself or in a separate methods section) as well as the parameters of the test (for example, if you ran a t-test, please indicate if it was one- or two-sided, etc.). Also, if you used parametric tests, please indicate if the data distribution was tested for normality (and if so, how). If not, you must state something to the effect that "Data distribution was assumed to be normal but this was not formally tested."

5) Abstract and title: The abstract should be no longer than 160 words and should communicate the significance of the paper for a general audience. The title should be less than 100 characters including spaces. Make the title concise but accessible to a general readership.

**6) Materials and methods: Should be comprehensive and not simply reference a previous publication for details on how an experiment was performed. Please provide full descriptions in the text for readers who may not have access to referenced manuscripts. For example, in the "Antibodies" paragraph of your methods section, you state: " Affinity-purified, polyclonal guinea pig and rabbit anti-syndapin I antibodies as well as guinea pig and rabbit anti-GST antibodies as well as rabbit anti-Cobl-like antibodies were raised and prepared as described (Qualmann et al., 1999; Izadi et al., 2018)." You must remove this and write out the full methodology. The same goes for all other places in the methods where you refer to methods "...as previously described."

7) All antibodies, cell lines, animals, and tools used in the manuscript should be described in full, including accession numbers for materials available in a public repository such as the Resource Identification Portal. Please be sure to provide the sequences for all of your primers/oligos and RNAi constructs in the materials and methods. You must also indicate in the methods the source, species, and catalog numbers (where appropriate) for all of your antibodies. Please also indicate the acquisition and quantification methods for immunoblotting/western blots.

8) Microscope image acquisition: The following information must be provided about the acquisition and processing of images:
a. Make and model of microscope
b. Type, magnification, and numerical aperture of the objective lenses

c. Temperature
d. Imaging medium
e. Fluorochromes
f. Camera make and model
g. Acquisition software
h. Any software used for image processing subsequent to data acquisition. Please include details and types of operations involved (e.g., type of deconvolution, 3D reconstitutions, surface or volume rendering, gamma adjustments, etc.).

9) References: There is no limit to the number of references cited in a manuscript. References should be cited parenthetically in the text by author and year of publication. Abbreviate the names of journals according to PubMed.

10) Supplemental materials: There are strict limits on the allowable amount of supplemental data. Articles may have up to 5 supplemental figures. Please also note that tables, like figures, should be provided as individual, editable files. A summary of all supplemental material should appear at the end of the Materials and methods section.

11) eTOC summary: A ~40-50-word summary that describes the context and significance of the findings for a general readership should be included on the title page. The statement should be written in the present tense and refer to the work in the third person.

12) Conflict of interest statement: JCB requires inclusion of a statement in the acknowledgements regarding competing financial interests. If no competing financial interests exist, please include the following statement: "The authors declare no competing financial interests." If competing interests are declared, please follow your statement of these competing interests with the following statement: "The authors declare no further competing financial interests."

**13) ORCID IDs: ORCID IDs are unique identifiers allowing researchers to create a record of their various scholarly contributions in a single place. Please note that ORCID IDs are now *required* for all authors. At resubmission of your final files, please be sure to provide your ORCID ID and those of all co-authors.

14) A separate author contribution section following the Acknowledgments. All authors should be mentioned and designated by their full names. We encourage use of the CRediT nomenclature.

Please note that JCB now requires authors to submit Source Data used to generate figures containing gels and Western blots with all revised manuscripts. This Source Data consists of fully uncropped and unprocessed images for each gel/blot displayed in the main and supplemental figures. For assays performed using capillary electrophoresis and/or immunoassay-based detection, authors should instead provide the electropherogram graph(s) for each experiment, plotting fluorescence/chemiluminescence intensity vs. molecular weight/size. Please be sure to provide one Source Data file for each figure gels, blots, and/or capillary electrophoresis assays along with your revised manuscript files. File names for Source Data figures should be alphanumeric without any spaces or special characters (i.e., SourceDataF#, where F# refers to the associated main figure number or SourceDataFS# for those associated with Supplementary figures). For traditional gels and blots, the lanes of the gels/blots should be labeled as they are in the associated figure, the place where cropping was applied should be marked (with a box), and molecular weight/size standards should be labeled wherever possible. For capillary electrophoresis assays, each trace in the graph should be color-coded and labeled to indicate which protein, gene, or sample is being measured (please try to avoid red/green combinations to accommodate our color-blind readers).

Source Data files will be made available to reviewers during evaluation of revised manuscripts and, if your paper is eventually published in JCB, the files will be directly linked to specific figures in the published article.

Source Data Figures should be provided as individual PDF files (one file per figure). Authors should endeavor to retain a minimum resolution of 300 dpi or pixels per inch. Please review our instructions for export from Photoshop, Illustrator, and PowerPoint here: https://rupress.org/jcb/pages/submission-guidelines#revised

Journal of Cell Biology now requires a data availability statement for all research article submissions. These statements will be published in the article directly above the Acknowledgments. The statement should address all data underlying the research presented in the manuscript. Please visit the JCB instructions for authors for guidelines and examples of statements at (https://rupress.org/jcb/pages/editorial-policies#data-availability-statement).

B. FINAL FILES:

Please upload the following materials to our online submission system. These items are required prior to acceptance. If you have any questions, contact JCB's Managing Editor, Lindsey Hollander (lhollander@rockefeller.edu).

-- An editable version of the final text (.DOC or .DOCX) is needed for copyediting (no PDFs).

-- High-resolution figure and MP4 video files: See our detailed guidelines for preparing your production-ready images, https://jcb.rupress.org/fig-vid-guidelines.

-- Cover images: If you have any striking images related to this story, we would be happy to consider them for inclusion on the journal cover. Submitted images may also be chosen for highlighting on the journal table of contents or JCB homepage carousel. Images should be uploaded as TIFF or EPS files and must be at least 300 dpi resolution.

It is JCB policy that if requested, original data images must be made available to the editors. Failure to provide original images upon request will result in unavoidable delays in publication. Please ensure that you have access to all original data images prior to final submission.

The license to publish form must be signed before your manuscript can be sent to production. A link to the license to publish form will be sent to the corresponding author only. Please take a moment to check your funder requirements before choosing the appropriate license.

Additionally, JCB encourages authors to submit a short video summary of their work. These videos are intended to convey the main messages of the study to a non-specialist, scientific audience. Think of them as an extended version of your abstract, or a short poster presentation. We encourage first authors to present the results to increase their visibility. The videos will be shared on social media to promote your work. For more detailed guidelines and tips on preparing your video, please visit https://rupress.org/jcb/pages/submission-guidelines#videoSummaries.

Thank you for your attention to these final processing requirements. Please revise and format the manuscript and upload materials within 14 days. If you need an extension for whatever reason, please let us know and we can work with you to determine a suitable revision period.

Please contact the journal office with any questions at cellbio@rockefeller.edu.

Thank you for this interesting contribution, we look forward to publishing your paper in Journal of Cell Biology.

Sincerely,

Tobias Meyer
Monitoring Editor
Journal of Cell Biology

Gabriele Stephan
Scientific Editor
Journal of Cell Biology

Our point-to-point response to editorial points

RE: JCB Manuscript #202509074RR-A

A. MANUSCRIPT ORGANIZATION AND FORMATTING:
1) Text limits: Character count for Articles is < 40,000, not including spaces. Count includes abstract, introduction, results, discussion, and acknowledgments. Count does not include title page, figure legends, materials and methods, references, tables, or supplemental legends.

OK. Checked: abstract, introduction, results, discussion and acknowledgments together are 39878 characters. The length of the manuscript thus is < 40 000.

2) Figures limits: Articles may have up to 10 main text figures.

OK. The manuscript includes 10 main text figures.

3) Figure formatting: Scale bars must be present on all microscopy images, including inset magnifications. Molecular weight or nucleic acid size markers must be included on all gel electrophoresis. Aspect ratios of images may not be altered.

All figures have been double-checked and comply with your guidelines.

*In order to accommodate readers with red-green color blindness, we encourage you to change the red/green color scheme in some of your figures.

Scientifically, we prefer a true-color representation and separate assignments of fluorescence to individual and not mixed color channels. We also prefer consistent use of colors in both double and triple colocalization evaluations, which is not possible in e.g. magenta/turquois. Finally, we think it is important to clearly distinguish true-color and basic-color fluorescence data from false-color representations, which are shown in gem (purple, magenta, orange, white). We acknowledge that this may require some measures to make sure that the data also is accessible for color blinds. **We made sure that for all red and green colocalizations also the corresponding single channel data are accessible in the (revised) manuscript. This applies to**

- Merged images in Fig. 1G-I, the single channel images are shown in Fig. S1E-F.
- Merged images shown in Fig. 2I-K, the single channel images are shown in Fig. S1H-J.
- Merged images in Fig. 4B-E, single channel images directly shown in Fig. 4B-E.
- Merged images in Fig. 5B,C, single channel images directly shown in Fig. 5B,C.
- Merged images in Fig. 7A-D (three channels), single channel images directly shown in Fig. 7A-D.
- Merged images in Fig. 8N,N, **the size has been minimally reduced in the revised manuscript in order to now show the single channel images next to the merged ones in Fig. 8M,N.**
- Merged images in Fig. S4F-L, single channel images directly shown in Fig. S4F-L.
- In bar/dot plots using red and green bars or shades thereof, the conditions are clearly labelled by text.
- In addition, we furthermore made sure that red and green colors in the figures can be distinguished by simply printing the figures in black & white instead of in color (thereby, the red chosen results in a darker grey than the green used throughout, i.e. the two colors can be distinguished)

4) Statistical analysis: Error bars on graphic representations of numerical data must be clearly described in the figure legend. The number of independent data points (n) represented in a graph must be indicated in the legend. Statistical methods should be explained in full in the materials and methods. For figures presenting pooled data the statistical measure should be defined in the figure legends. Please also be sure to indicate the statistical tests used in each of your experiments (either in the figure legend itself or in a separate methods section) as well as the parameters of the test (for example, if you ran a t-test, please indicate if it was one- or two-sided, etc.). Also, if you used parametric tests, please indicate if the data distribution was tested for normality (and if so, how). If not, you must state something to the effect that "Data distribution was assumed to be normal but this was not formally tested."

The legends of the manuscript provide complete information on the statistical tests used. **We added the explicit information that unpaired, two-tailed t-test were used when t-tests were applied to the legends of the revised manuscript** (before, this information was only accessible in the numerical source data file provided for all quantitative data panels, which includes all individual numerical data and the statistics).
The Material and Method section of the revised manuscript has been expanded and now e.g. also describes the tests used in the normality distribution analyses in more detail.

5) Abstract and title: The abstract should be no longer than 160 words and should communicate the significance of the paper for a general audience. The title should be less than 100 characters including spaces. Make the title concise but accessible to a general readership.

OK. The abstract is 158 words. The title has 99 characters incl. spaces. A running title is included in the title page information.

**6) Materials and methods: Should be comprehensive and not simply reference a previous publication for details on how an experiment was performed. Please provide full descriptions in the text for readers who may not have access to referenced manuscripts. For example, in the "Antibodies" paragraph of your methods section, you state: " Affinity-purified, polyclonal guinea pig and rabbit anti-syndapin I antibodies as well as guinea pig and rabbit anti-GST antibodies as well as rabbit anti-Cobl-like antibodies were raised and prepared as described (Qualmann et al., 1999; Izadi et al., 2018)." You must remove this and write out the full methodology. The same goes for all other places in the methods where you refer to methods "...as previously described."

We have expanded some descriptions of methods or tools in the revised manuscript. All methods are more or less briefly described but provide the full information required. References given additionally merely refer to the fact that this method had been established in a peer-reviewed scientific study before and are not needed to reproduce the methods.

7) All antibodies, cell lines, animals, and tools used in the manuscript should be described in full, including accession numbers for materials available in a public repository such as the Resource Identification Portal. Please be sure to provide the sequences for all of your primers/oligos and RNAi constructs in the materials and methods. You must also indicate in the methods the source, species, and catalog numbers (where appropriate) for all of your antibodies. Please also indicate the acquisition and quantification methods for immunoblotting/western blots.

Antibodies are listed with species, company and catalog numbers in Material and Methods. Primers are listed in Table S1.

Cell lines, animals and further tools are described in full. Material and Methods also includes the details on immunoblottings and their detection.

8) Microscope image acquisition: The following information must be provided about the acquisition and processing of images:

a. Make and model of microscope

b. Type, magnification, and numerical aperture of the objective lenses

c. Temperature

d. Imaging medium

e. Fluorochromes

f. Camera make and model

g. Acquisition software

h. Any software used for image processing subsequent to data acquisition. Please include details and types of operations involved (e.g., type of deconvolution, 3D reconstitutions, surface or volume rendering, gamma adjustments, etc.).

The required information on the equipment, tools and conditions used for the different types of microscopy are provided in the Material and Methods section of the revised manuscript.

9) References: There is no limit to the number of references cited in a manuscript. References should be cited parenthetically in the text by author and year of publication. Abbreviate the names of journals according to PubMed.

We edited the references. **The revised manuscript now fully complies with the format of JCB.**

10) Supplemental materials: There are strict limits on the allowable amount of supplemental data. Articles may have up to 5 supplemental figures. Please also note that tables, like figures, should be provided as individual, editable files. A summary of all supplemental material should appear at the end of the Materials and methods section.

We did limit our supplemental figures to 5.
A summary of all supplemental material has been included in the revised manuscript at the end of the Material and Methods section.

11) eTOC summary: A ~40-50-word summary that describes the context and significance of the findings for a general readership should be included on the title page. The statement should be written in the present tense and refer to the work in the third person.

We added an eTOC to the title page of the revised manuscript.

12) Conflict of interest statement: JCB requires inclusion of a statement in the acknowledgements regarding competing financial interests. If no competing financial interests exist, please include the following statement: "The authors declare no competing financial interests." If competing interests are declared, please follow your statement of these competing interests with the following statement: "The authors declare no further competing financial interests."

As requested, we added the word „financial“ and moved the Conflict of Interest Statement to the acknowledgements in the revised manuscript.

**13) ORCID IDs: ORCID IDs are unique identifiers allowing researchers to create a record of their various scholarly contributions in a single place. Please note that ORCID IDs are now *required* for all authors. At resubmission of your final files, please be sure to provide your ORCID ID and those of all co-authors.

The ORCID-IDs of all authors are now included in the title page of the revised manuscript.

14) A separate author contribution section following the Acknowledgments. All authors should be mentioned and designated by their full names. We encourage use of the CRediT nomenclature.

All authors are now designated by their full names in the acknowledgments.
The text of the acknowledgements has been agreed to by all authors (after having been a sensitive issue) and therefore should better not be changed at this point.

Please note that JCB now requires authors to submit Source Data used to generate figures containing gels and Western blots with all revised manuscripts. This Source Data consists of fully uncropped and unprocessed images for each gel/blot displayed in the main and supplemental figures. For assays performed using capillary electrophoresis and/or immunoassay-based detection, authors should instead provide the electropherogram graph(s) for each experiment, plotting fluorescence/chemiluminescence intensity vs. molecular weight/size. Please be sure to provide one Source Data file for each figure gels, blots, and/or capillary electrophoresis assays along with your revised manuscript files. File names for Source Data figures should be alphanumeric without any spaces or special characters (i.e., SourceDataF#, where F# refers to the associated main figure number or SourceDataFS# for those associated with Supplementary figures). For traditional gels and blots, the lanes of the gels/blots should be labeled as they are in the associated figure, the place where cropping was applied should be marked (with a box), and molecular weight/size standards should be labeled wherever possible. For capillary electrophoresis assays, each trace in the graph should be color-coded and labeled to indicate which protein, gene, or sample is being measured (please try to avoid red/green combinations to accommodate our color-blind readers).
Source Data files will be made available to reviewers during evaluation of revised manuscripts and, if your paper is eventually published in JCB, the files will be directly linked to specific figures in the published article.
Source Data Figures should be provided as individual PDF files (one file per figure). Authors should endeavor to retain a minimum resolution of 300 dpi or pixels per inch. Please review our instructions for export from Photoshop, Illustrator, and PowerPoint here:
https://rupress.org/jcb/pages/submission-guidelines#revised

We have split the Source Data compilation, which we provided for peer-reviewing and which already showed all blots and gels and the cropping positions, into the respective single figures related to the main or supplementary figures and named them according to your guidelines. The source data file also are now cited individually in the legends of the respective figure. The numerical source data compilation is cited in the Statistics subchapter of the Material and Method section of the revised manuscript.
We have updated several blots/gels, which either only showed some lanes of a blot (whereas others were omitted by insertion of white lines) or which showed lanes in a different order (also blot pieces separated by white lines), with the respective full blots/gels in the Source Data. It is thereby always directly proven that the compiled data is indeed from the same gel/blot – which of course is a formal prerequisite for white line insertion.

Please note that white lines that still remain in the source data simply show molecular weight standards that are from the same blot but were recorded in a different fluorescence channel.

Journal of Cell Biology now requires a data availability statement for all research article submissions. These statements will be published in the article directly above the Acknowledgments. The statement should address all data underlying the research presented in the manuscript. Please visit the JCB instructions for authors for guidelines and examples of statements at (https://rupress.org/jcb/pages/editorial-policies#data-availability-statement).

The Data Availability Statement was already included in the manuscript. The positioning of it within the manuscript text also was according to the instructions.

B. FINAL FILES:
Please upload the following materials to our online submission system. These items are required prior to acceptance. If you have any questions, contact JCB's Managing Editor, Lindsey Hollander (lhollander@rockefeller.edu).
-- **An editable version of the final text (.DOC or .DOCX) is needed** for copyediting (no PDFs).
-- **High-resolution figure** and MP4 video files: See our detailed guidelines for preparing your production-ready images, https://jcb.rupress.org/fig-vid-guidelines.
-- **Cover images**: If you have any striking images related to this story, we would be happy to consider them for inclusion on the journal cover. Submitted images may also be chosen for highlighting on the journal table of contents or JCB homepage carousel. Images should be uploaded as TIFF or EPS files and must be at least 300 dpi resolution.
****It is JCB policy that if requested, original data images must be made available to the editors.** Failure to provide original images upon request will result in unavoidable delays in publication. Please ensure that you have access to all original data images prior to final submission.**
The **license to publish form must be signed before your manuscript can be sent to production. A link to the license to publish form will be sent to the corresponding author only. Please take a moment to check your funder requirements before choosing the appropriate license.**
Additionally, JCB encourages authors to submit a short video summary of their work. These videos are intended to convey the main messages of the study to a non-specialist, scientific audience. Think of them as an extended version of your abstract, or a short poster presentation. We encourage first authors to present the results to increase their visibility. The videos will be shared on social media to promote your work. For more detailed guidelines and tips on preparing your video, please visit https://rupress.org/jcb/pages/submission-guidelines#videoSummaries.
Thank you for your attention to these final processing requirements. **Please revise and format the manuscript and upload materials within 14 days.** If you need an extension for whatever reason, please let us know and we can work with you to determine a suitable revision period.
