# Supplementary material for: The N-Ank protein Ankrd24 shapes neurons and couples to syndapin and the F-actin promoter Cobl-like
Source: J Cell Biol. 2026 Oct 21;225(11):e202509074. doi: 10.1083/jcb.202509074 (PMC13712265; doi:10.1083/jcb.202509074)
Supplement: Supplementary file 2 [file jcb_202509074_sourcedataf1.pdf]

Figure 1

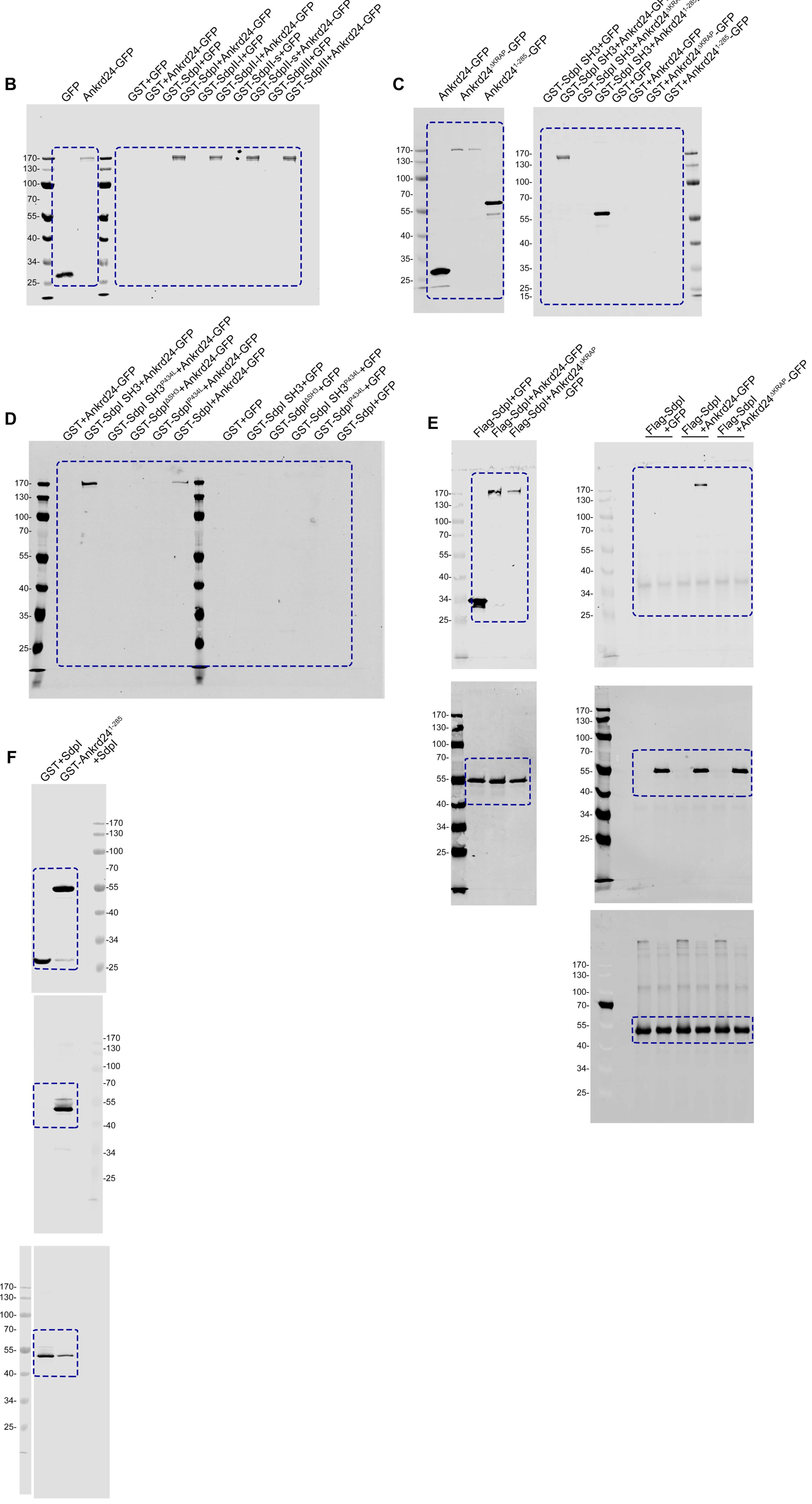
B
GFP
Ankrd24-GFP
GST+GFP
GST+Ankrd24-GFP
GST-SdpI+GFP
GST-SdpI+Ankrd24-GFP
GST-SdpII-l+GFP
GST-SdpII-l+Ankrd24-GFP
GST-SdpII-s+GFP
GST-SdpII-s+Ankrd24-GFP
GST-SdpIII+GFP
GST-SdpIII+Ankrd24-GFP
C
Ankrd24-GFP
Ankrd24ΔKRAP-GFP
Ankrd241-285-GFP
GST-SdpI SH3+GFP
GST-SdpI SH3+Ankrd24-GFP
GST-SdpI SH3+Ankrd24ΔKRAP-GFP
GST-SdpI SH3+Ankrd241-285-GFP
GST+GFP
GST+Ankrd24-GFP
GST+Ankrd24ΔKRAP-GFP
GST+Ankrd241-285-GFP
D
GST+Ankrd24-GFP
GST-SdpI SH3+Ankrd24-GFP
GST-SdpI SH3P434L+Ankrd24-GFP
GST-SdpIΔSH3+Ankrd24-GFP
GST-SdpIP434L+Ankrd24-GFP
GST-SdpI+Ankrd24-GFP
GST+GFP
GST-SdpI SH3+GFP
GST-SdpIΔSH3+GFP
GST-SdpI SH3P434L+GFP
GST-SdpIP434L+GFP
GST-SdpI+GFP
E
Flag-SdpI+GFP
Flag-SdpI+Ankrd24-GFP
Flag-SdpI+Ankrd24ΔKRAP-GFP
Flag-SdpI +GFP
Flag-SdpI +Ankrd24-GFP
Flag-SdpI +Ankrd24ΔKRAP-GFP
F
GST+SdpI
GST-Ankrd241-285 +SdpI
170-
130-
100-
70-
55-
40-
34-
35-
25-
15-
