# Supplementary material for: The N-Ank protein Ankrd24 shapes neurons and couples to syndapin and the F-actin promoter Cobl-like
Source: J Cell Biol. 2026 Oct 21;225(11):e202509074. doi: 10.1083/jcb.202509074 (PMC13712265; doi:10.1083/jcb.202509074)
Supplement: Supplementary file 3 [file jcb_202509074_sourcedataf10.pdf]

Figure 10

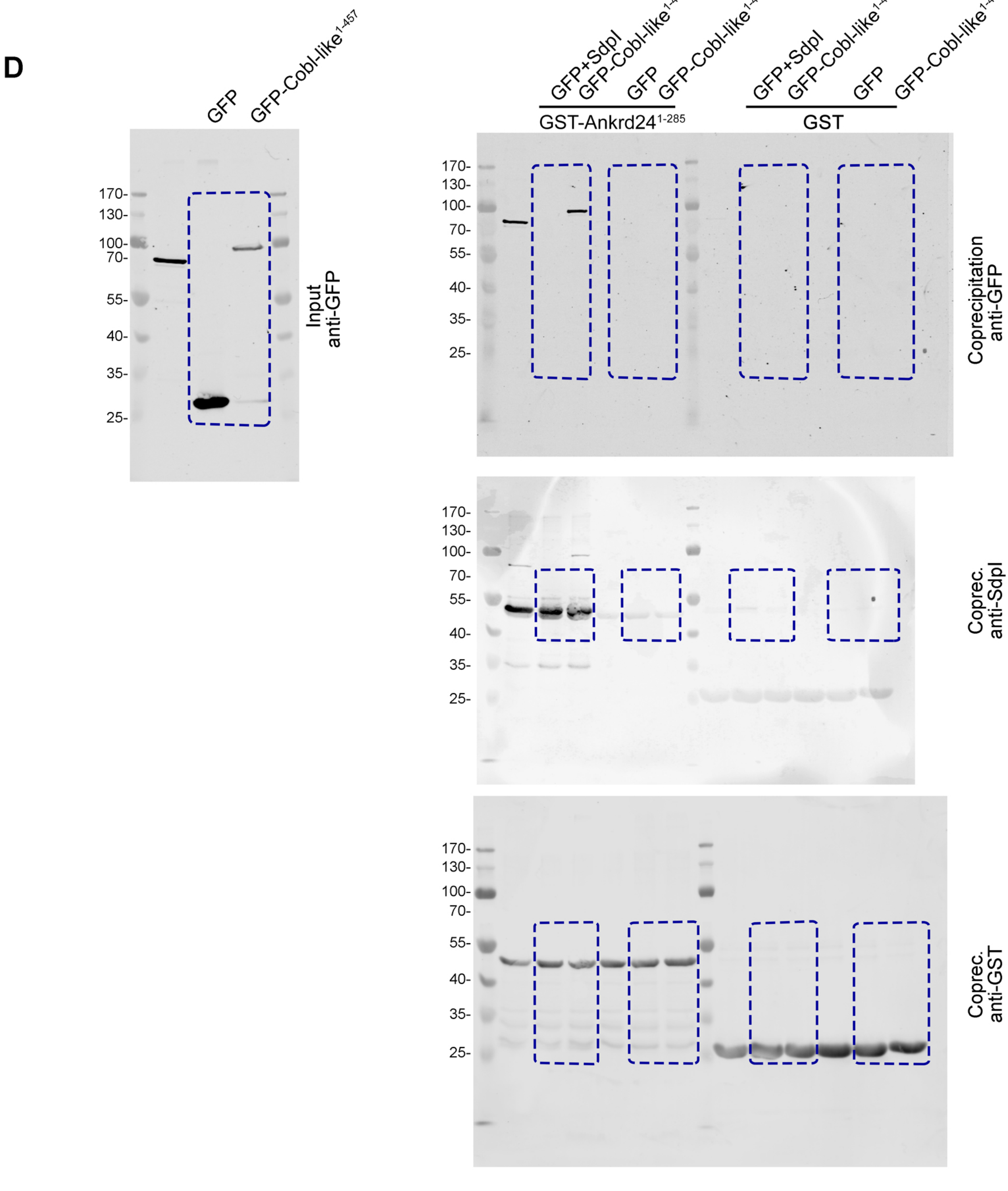
D
GFP
GFP-Cobl-like1-457
170-
130-
100-
70-
55-
40-
35-
25-
Input
anti-GFP
GFP+Sdpl
GFP-Cobl-like1-457+Sdpl
GFP
GFP-Cobl-like1-457
GST-Ankrd241-285
GST
Coprecipitation
anti-GFP
Coprec.
anti-Sdpl
Coprec.
anti-GST
