# Supplementary material for: The N-Ank protein Ankrd24 shapes neurons and couples to syndapin and the F-actin promoter Cobl-like
Source: J Cell Biol. 2026 Oct 21;225(11):e202509074. doi: 10.1083/jcb.202509074 (PMC13712265; doi:10.1083/jcb.202509074)
Supplement: Supplementary file 4 [file jcb_202509074_sourcedataf2.pdf]

Figure 2

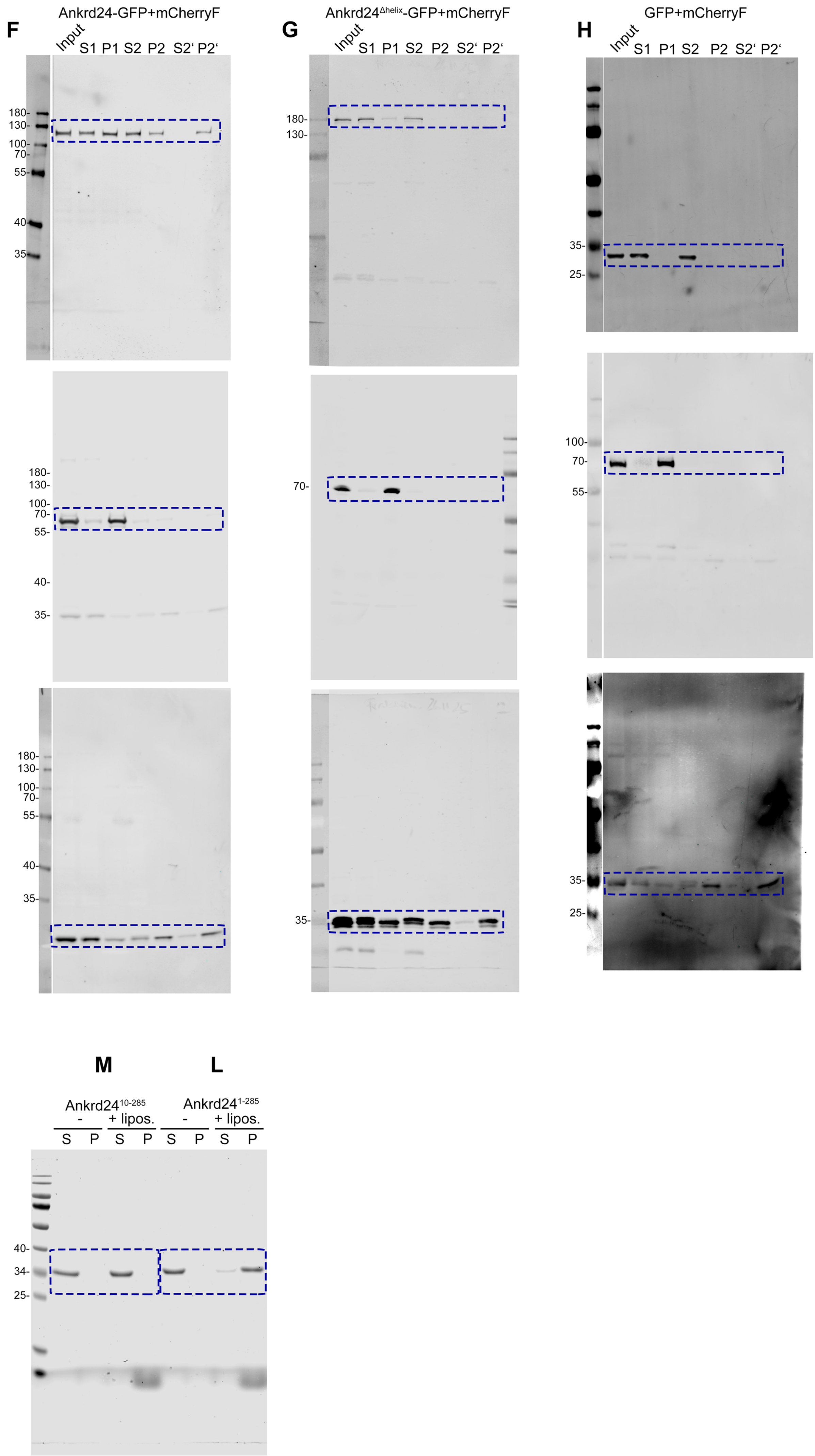
F
Ankrd24-GFP+mCherryF
Input S1 P1 S2 P2 S2‘ P2‘
180-
130-
100-
70-
55-
40-
35-
G
Ankrd24Δhelix-GFP+mCherryF
Input S1 P1 S2 P2 S2‘ P2‘
180-
130-
70-
35-
H
GFP+mCherryF
Input S1 P1 S2 P2 S2‘ P2‘
35-
25-
100-
70-
55-
M
L
Ankrd2410-285
Ankrd241-285
-
+ lipos.
S P S P S P S P
40-
34-
25-
