# Supplementary material for: The N-Ank protein Ankrd24 shapes neurons and couples to syndapin and the F-actin promoter Cobl-like
Source: J Cell Biol. 2026 Oct 21;225(11):e202509074. doi: 10.1083/jcb.202509074 (PMC13712265; doi:10.1083/jcb.202509074)
Supplement: Supplementary file 5 [file jcb_202509074_sourcedataf4.pdf]

Figure 4

A

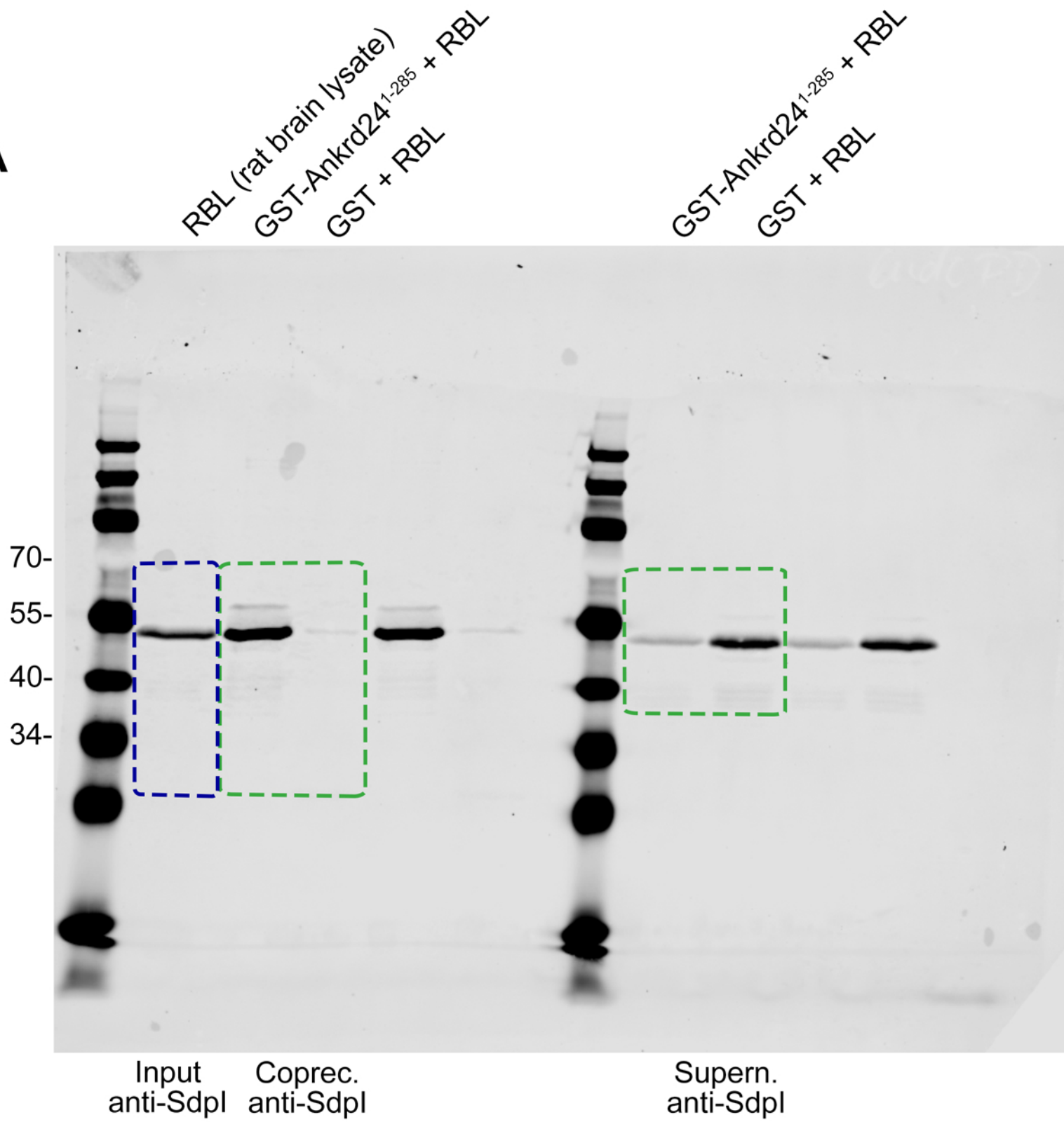

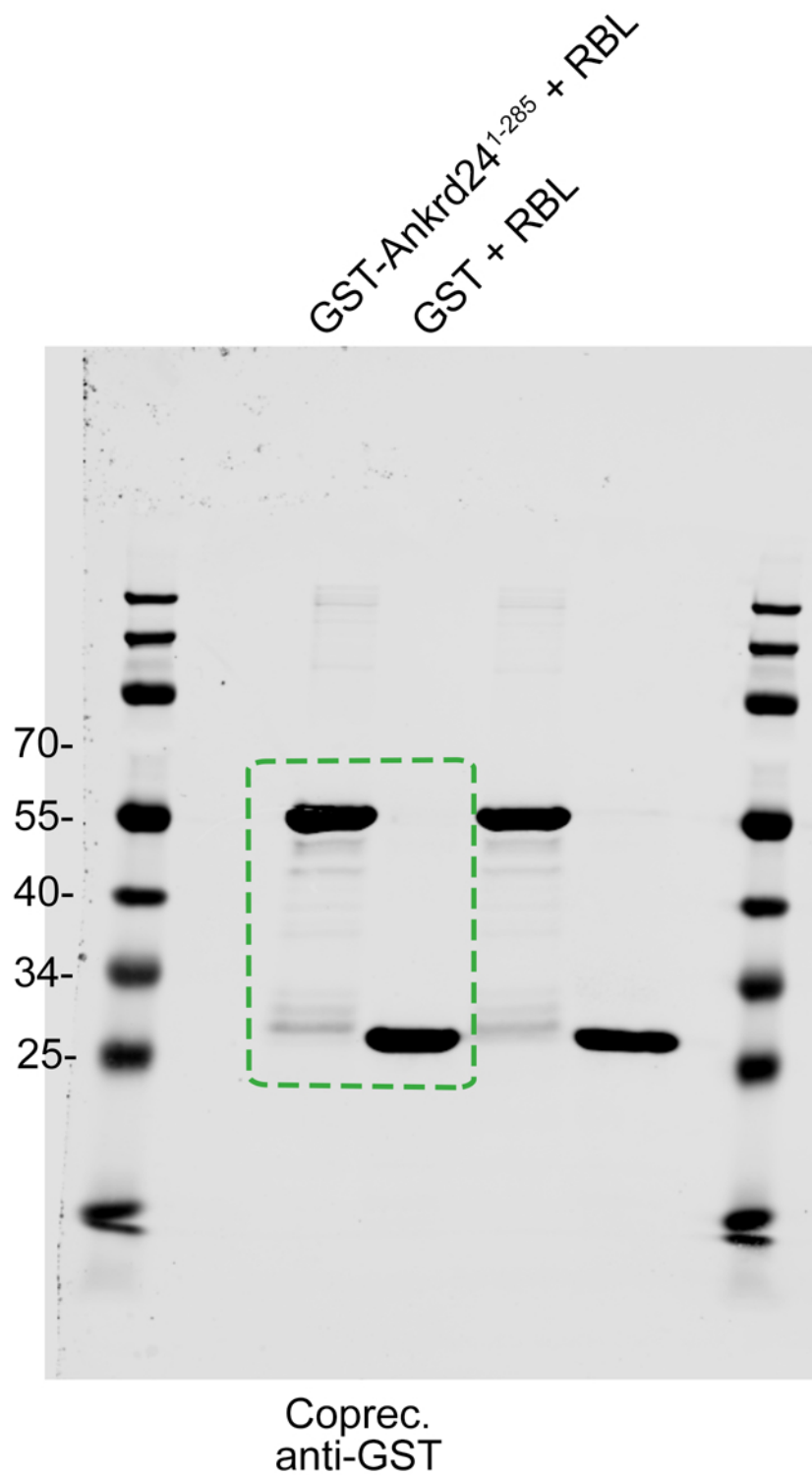

green boxes represent blot areas
shown with flipped lane order in Fig. 4
(therefore the white line insertion in Fig. 4)
