# Supplementary material for: The N-Ank protein Ankrd24 shapes neurons and couples to syndapin and the F-actin promoter Cobl-like
Source: J Cell Biol. 2026 Oct 21;225(11):e202509074. doi: 10.1083/jcb.202509074 (PMC13712265; doi:10.1083/jcb.202509074)
Supplement: Supplementary file 6 [file jcb_202509074_sourcedataf8.pdf]

Figure 8

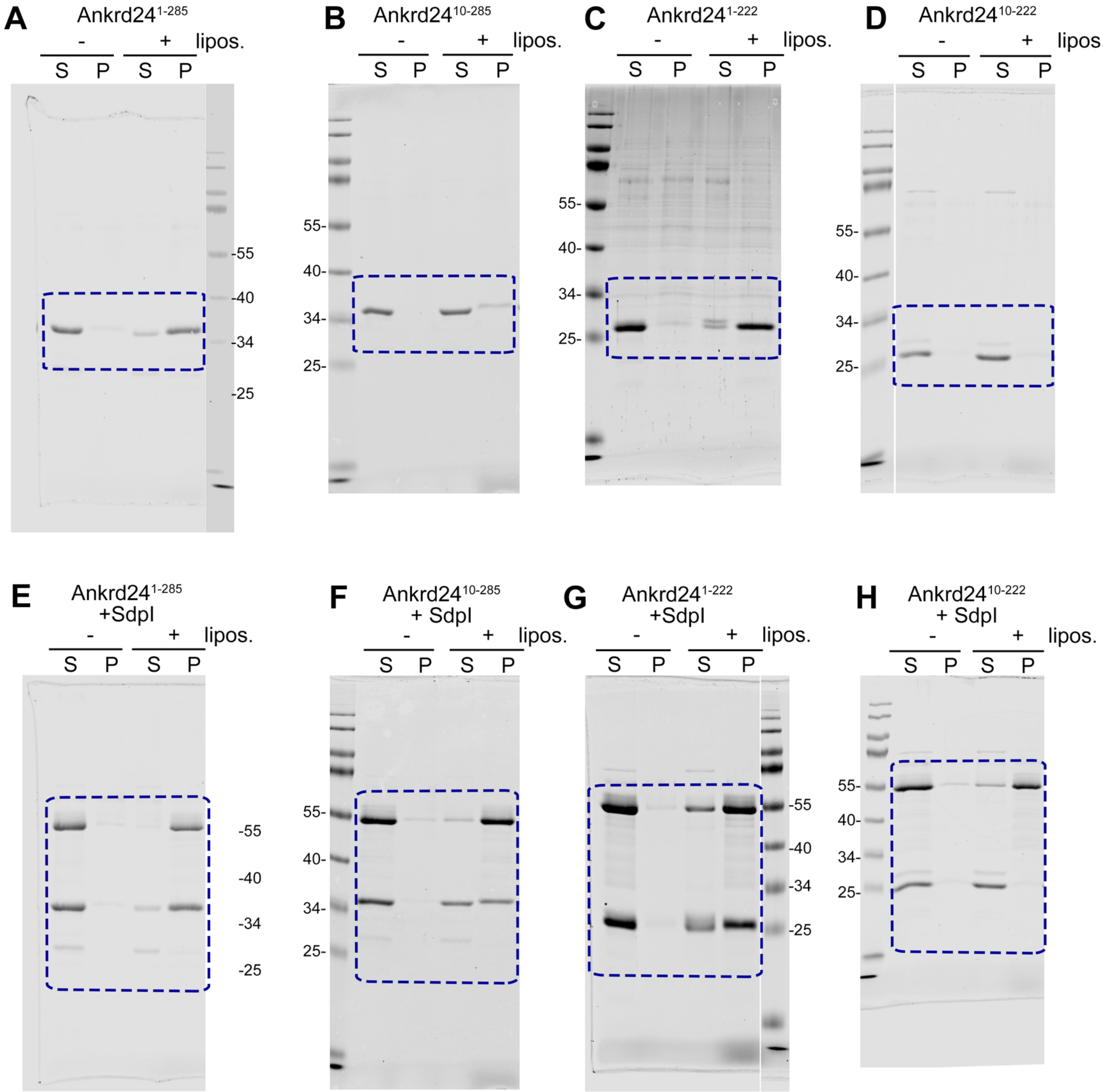

A
Ankrd24 1-285
- + lipos.
S P S P
-55
-40
-34
-25
B
Ankrd24 10-285
- + lipos.
S P S P
55-
40-
34-
25-
C
Ankrd24 1-222
- + lipos.
S P S P
55-
40-
34-
25-
D
Ankrd24 10-222
- + lipos.
S P S P
55-
40-
34-
25-
E
Ankrd24 1-285
+SdpI
- + lipos.
S P S P
-55
-40
-34
-25
F
Ankrd24 10-285
+ SdpI
- + lipos.
S P S P
55-
40-
34-
25-
G
Ankrd24 1-222
+SdpI
- + lipos.
S P S P
-55
-40
-34
-25
H
Ankrd24 10-222
+ SdpI
- + lipos.
S P S P
55-
40-
34-
25-
