# Supplementary material for: The N-Ank protein Ankrd24 shapes neurons and couples to syndapin and the F-actin promoter Cobl-like
Source: J Cell Biol. 2026 Oct 21;225(11):e202509074. doi: 10.1083/jcb.202509074 (PMC13712265; doi:10.1083/jcb.202509074)

Supplementary Figure S1

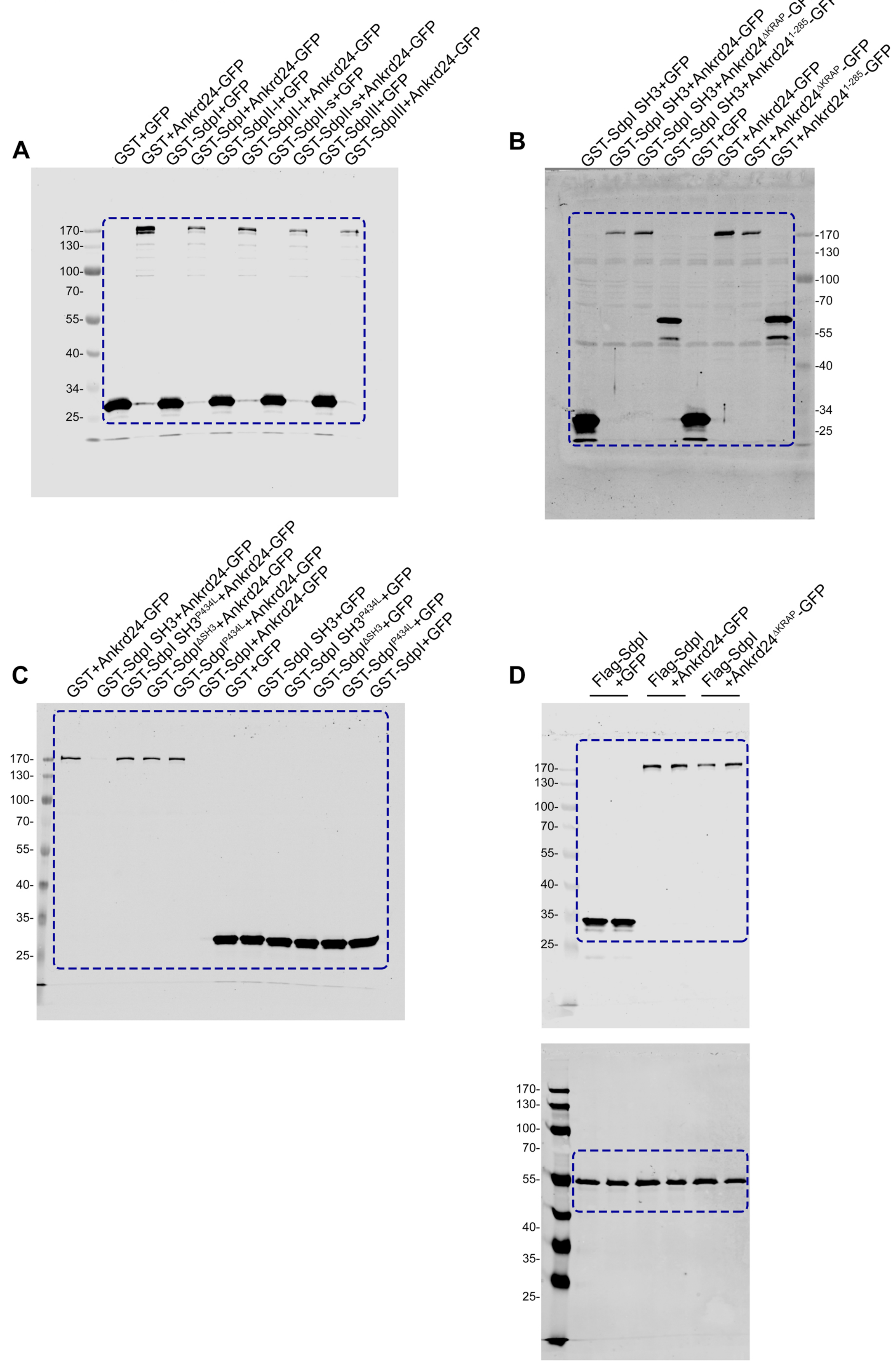

Supplement: Supplementary file 7 [file jcb_202509074_sourcedatafs1.pdf]
